# Diagnostic of students' misconceptions using the Biological Concepts Instrument (BCI): A method for conducting an educational needs assessment

**DOI:** 10.1371/journal.pone.0176906

**Published:** 2017-05-11

**Authors:** Annie Champagne Queloz, Michael W. Klymkowsky, Elsbeth Stern, Ernst Hafen, Katja Köhler

**Affiliations:** 1 Institute of Molecular Systems Biology, ETH Zürich, Zürich, Switzerland; 2 Molecular, Cellular, and Developmental Biology, University of Colorado, Boulder, Colorado, United States of America; 3 Institute for Educational Science, ETH Zürich, ETH Zürich, Zürich, Switzerland; Universidade de Brasilia, BRAZIL

## Abstract

Concept inventories, constructed based on an analysis of students’ thinking and their explanations of scientific situations, serve as diagnostics for identifying misconceptions and logical inconsistencies and provide data that can help direct curricular reforms. In the current project, we distributed the Biological Concepts Instrument (BCI) to 17-18-year-old students attending the highest track of the Swiss school system (Gymnasium). Students’ performances on many questions related to evolution, genetics, molecular properties and functions were diverse. Important common misunderstandings were identified in the areas of evolutionary processes, molecular properties and an appreciation of stochastic processes in biological systems. Our observations provide further evidence that the BCI is efficient in identifying specific areas where targeted instruction is required. Based on these observations we have initiated changes at several levels to reconsider how biological systems are presented to university biology studies with the goal of improving student’s foundational understanding.

## Introduction

Educational authorities are increasingly motivated to create and apply empirical approaches to uncover students’ misunderstandings and to initiate curricular reforms that integrate new technologies, educational approaches, and interdisciplinary insights. The literature on the conceptual understanding of students in biology has increased considerably since 2010, helping to develop new curricula based on what a student needs to understand in order to develop authentic conceptual understanding [1–3]. In this light, conceptual understanding requires the ability to create a network of knowledge, ideally in an interdisciplinary perspective, to transfer and apply such knowledge in diverse contexts [4].

Many research studies on conceptual understanding aim at diagnosing misconceptions. Misconceptions have been defined as naive or incomplete persistent explanations of scientific concepts shared by many students [5]. Misconceptions often go unrecognized and persist during the course of instruction if not addressed [6,7]. Cooper and Klymkowsky [8] refer to didaskalogenic misconceptions as those resulting from previous instruction, commonly generated through the use of short cuts and analogies (e.g., lock and key or ball and stick models) that oversimplify core ideas and their scientific basis.

Misconceptions are often not readily revealed by the usual summative assessments carried out in academic institutions, because the majority of such assessments are constructed to evaluate the retention of factual knowledge and thus measure memorization capacity and attentiveness [9]. Consequently, such assessments rarely address authentic conceptual understanding. According to Schneider and Stern [10], instructional strategies addressing misconceptions or prior knowledge are essential for optimal learning and to help students obtaining an understanding that is closer to expert thinking. For example, Klymkowsky et al. [11] have developed an undergraduate introductory biology course focused particularly on social evolutionary mechanisms, stochastic (evolutionary and molecular) processes and some core ideas (cellular continuity, evolutionary homology, molecular interactions, coupled chemical reactions and molecular machines). Previous studies indicate that many students hold important misconceptions concerning those concepts. Given the diversity of biological topics, multiple questionnaires are available to reveal misconceptions at the molecular, microscopic or macroscopic level (Table 1).

**Table 1:** Concept inventories covering biology or biochemistry knowledge.

Name	Type of questions	Number of questions	References
**Concept inventories on biological knowledge**			
Biological Concepts Instrument	MC	29	[20]
Biological Experimental Design Concept Inventory	MC	14	[22]
Central Dogma Concept Inventory	MC	23	[23]
Conceptual Inventory of Natural Selection	MC	20	[24]
Dominance Concept Inventory	MC-TF-2/3MC	16	[25]
Evolutionary Development Biology Concept Inventory	MC	11	[26]
Genetic Drift Inventory	TF	23	[27]
Genetics Concept Assessment	MC	25	[28]
Genetics Literacy Assessment Instrument	MC	31	[29]
Host-Pathogen Interactions Concept Inventory	MC	18	[30]
Introductory Molecular and Cell Assessment	MC	24	[31]
Lac Operon Concept Inventory	MC	12	[32]
Meiosis Concept Inventory	MC	17	[33]
Molecular Biology Capstone Assessment	TF	18	[34]
Open Response Instrument	OER	5	[35]
Photosynthesis: Diagnostic Question Clusters	MC-TF-OER	?	[36]
**Concept inventories on biochemical knowledge±**			
Chemical Concepts Inventory	MC	22	[37]
Diffusion and Osmosis Diagnostic Test	2/3MC	12	[38]
Dynamics Concept Inventory	MC	29	[13]
Enzyme-Substrate Interactions Concept Inventory	MC	15	[39]
Heat and Energy Concept Inventory	MC	36	[40]
Osmosis and Diffusion Conceptual Assessment	2/3MC	18	[41]
Redox Concept Inventory	MC	?	[42]
Thermal and Transport Science Concept Inventory	MC	12	[14]
Thermochemistry Concept Inventory	MC	10	[43]

MC: multiple-choice; TF: true/false; 2/3MC: two-tier multiple-choice; OER: Open-ended response.

Concept inventories are typically multiple-choice questionnaires based on students’ thinking, i.e., the distractors (the wrong answers) correspond to common and persistent misconceptions shared by many students. The development of concept inventories/tests is a challenging process where oral or written answers of large numbers of students to questions addressing different concepts in biology have to be collected to develop distractors that represent student thinking. However, the time and the costs of developing those questionnaires are important. Thus, some questionnaires are not based on words or thinking of students, but on experience on student misunderstanding by experts (often called Delphi approach [12]) (see the Dynamics Concept Inventory [13] and the Thermal and Transport Science Concept Inventory [14] in Table 1).

As suggested by Hestenes [15], who developed the Force Concept Inventory to evaluate the understanding of Newton’s laws, “the Inventory, therefore, is not a test of intelligence; it is a probe of belief systems”. The most critical point of those questionnaires is the attractiveness of the distractors, since students may simply ignore them if they do not address a misconception that the student actually holds. Consequently, they may select the correct answer by eliminating (or ignoring) the weak distractors rather than because they understand why the correct answer is correct [16]. The majority of questions/answers from the concept inventories cited in Table 1 were developed by interviewing students or by analysing student answers to open-ended questions. Interestingly, students’ understanding is often based on teleological (everything is aimed at a goal or a better situation) and anthropocentric (to refer to humans) thinking on how biological mechanisms should work. For example, “molecules know where to go (or need to go)” or “evolution produces organisms for conditions that are structurally and/or metabolically better suited” [17–19].

For the current project, the Biological Concepts Instrument (BCI) was selected because it covers a broad spectrum of concepts frequently taught in biology courses at the undergraduate level [20]. It is composed of 30 questions related to various topics including evolutionary mechanisms, structure and function of molecules, molecular interactions, stochastic processes, genetics, energetics and experimental design. The BCI content was validated with disciplinary experts. The BCI has been successfully used in the US to assess biological misconceptions in students and teachers [20]. The questionnaire is written in a “student language” style, making the questions and the distractors closer to students’ thinking. Additionally, the entire questionnaire can be completed in only 30 minutes and it is freely available online. The BCI can be used for an educational needs assessment, i.e. to evaluate the gap between a problematic (misunderstanding of students) and the expected (an authentic conceptual understanding) situation [21].

Using the BCI, we aimed to identify specific areas where targeted instruction might be useful to promote and improve a conceptual understanding. Therefore, we investigated the misconceptions in a group of students before their entrance into university, at the end of the Swiss Gymnasium level (obligatory educational path conducting to university studies in Switzerland). The information produced from this survey can be used to initiate reforms in the content addressed in introductory biology lectures at university to meet the educational needs of 1st-year undergraduates. Furthermore, the data is also informative in respect to the education of future biology teachers at Swiss universities.

## Materials and methods

### Participants

This study was conducted in central and eastern, German-speaking regions of Switzerland. The first cohort consisted of 211 Gymnasium students between November 2012 and June 2013 (12 schools) and a second cohort contained 264 Gymnasium students between January 2014 and May 2014 (9 schools). A total of 475 students were interviewed. Some teachers at four schools have participated twice with different cohorts of students. The students were in their last year of Gymnasium and were between 17 and 18 years old. For confidentiality reasons, none of the schools are identified here.

### Ethical considerations

Because the testing was anonymous (the surveys were not identified by the participants' names) and voluntary, and it took place in students' natural settings (biology lessons at school), the approval of the ETH Ethics Commission was not required for these studies. The purpose of the study (collecting and analyzing data for publication as a Ph.D. thesis) was explained in advance to the teachers and students, and students were presented with a survey on biology. The consent of the students was obtained by their participation in the survey, i.e., non-participation in the survey was considered as a non-consent. Students’ participation implied their agreement to the publication of the results.

### Procedure of translation of the BCI

The BCI was translated into German by a standardized translation/back-translation procedure (WHO. 2014. Home Page. http://www.who.int/substance_abuse/research_tools/translation/en/ (accessed February 27, 2017)). One independent native German-speaking expert did the translation, which was validated with high school teachers and didactic experts to adapt the vocabulary to the one used in schools. The validation of the back-translation (done by an native English-speaking expert) was done by thirteen graduate students who were fluent in English. Statistical comparisons of the students’ results were done using the R 3.0.2 statistical software package (R. Development Core Team 2013). The difference in BCI scores between the original and the back-translated versions were analyzed by McNemar’s Chi-Square Test. This test assesses the significance of the difference between the performances of both versions on individual questions [40,44]. There was no significant difference in performances between the original and the back-translated version on individual questions (McNemar test, χ^2^(df = 1) = 1.33, p = 0.248 ≤ p ≥ χ^2^(df = 1) = 0.00, p = 1.00), alpha < 0.05). In addition a paired-sample t-test was conducted to compare BCI scores of the original and the back-translated versions; no significant difference in scores between both versions was found (t(-0.49), df = 12, p-value = 0.6318) (Shapiro-Wilk normality test, W = 0.9453, p-value = 0.1797, alpha < 0.05). The German version is available in the Supporting Information S1 Text.

### Procedure of distribution

All participants were tested without any special preparation, i.e., there was no attempt made to alter instruction based on students’ apparent misconceptions. A short introduction to explain the idea of our project before the testing was sufficient to motivate the majority of them to participate seriously. It was explicitly explained to participants to select the "best answer", corresponding to the best statement explaining the question. Students were given 25 minutes to complete the adapted BCI, which consisted of 24 multiple-choice questions (the selection of questions is described in the Supporting Information S1 Text). According to the availability of Gymnasium teachers during the semester, the BCI was distributed at different times between November 2011 and June 2012 for the first cohort and between January and June 2013 for the second cohort.

### Analysis of course materials

To determine whether teachers addressed concepts found in the BCI, we examined lessons plans of biology courses and biology textbooks used at that level. The analysis of the lesson plan of each teacher who participated in this project and biology textbooks revealed only minor differences. We observed that basics in genetics (Mendel, inheritance, linkage, chromosomes, genes, DNA), evolution (Darwin, Lamarck, natural selection, genetic drift, mutations), cell biology (diffusion, osmosis, cell structure, mitosis (often taught in relationship to meiosis)), reproduction (meiosis/mitosis, organs, hormonal systems), energetics (photosynthesis, respiration, ATP synthesis, digestion, nutrition) were addressed during instruction. Randomness and thermodynamic properties were not found in the majority of the biology lesson plans.

### Statistical analysis

#### Comparisons of scores between each group of participants

Statistical comparisons of the students’ results were done using the R 3.0.2 statistical software package (R. Development Core Team 2013). Individual students’ scores were analyzed according to their rank order using a nonparametric Kruskal-Wallis test, which does not assume that the data sets possess a normal distribution. By applying the comparison “pairwise.wilcox.test” in R, the mean score of the participants’ groups were compared. Performance comparisons of the participants were carried out by comparing the degree of correctness (more frequently called Item difficulty) of each BCI item. The degree of correctness is the overall proportion of students choosing the correct answer to a particular question [45]; easier questions show a higher degree of correctness.

## Results

### Comparison of students’ overall performance in the two cohorts

There was no significant difference in the distribution of individual students’ scores between the two cohorts (Kruskal-Wallis, χ^2^(df = 1) = 0.153, p-value = 0.696, alpha < 0.05). The point in the semester when the BCI was administered did not affect students' BCI scores. More precisely, the performances of participants who have completed the BCI in June, after instruction, were not significantly higher than students who have completed the questionnaire earlier in the semester (essentially before instruction) (see Fig A in Supporting Information S1 Text and see S1 Dataset for all students’ performance data).

All participants' results are available in the Supporting Information S1 Text and S1 Dataset. Here, we show a sample of questions demonstrating diverse students' performances on specific biological concepts.

### Evolution (Q4, Q5, Q11)

**Fig 1:**
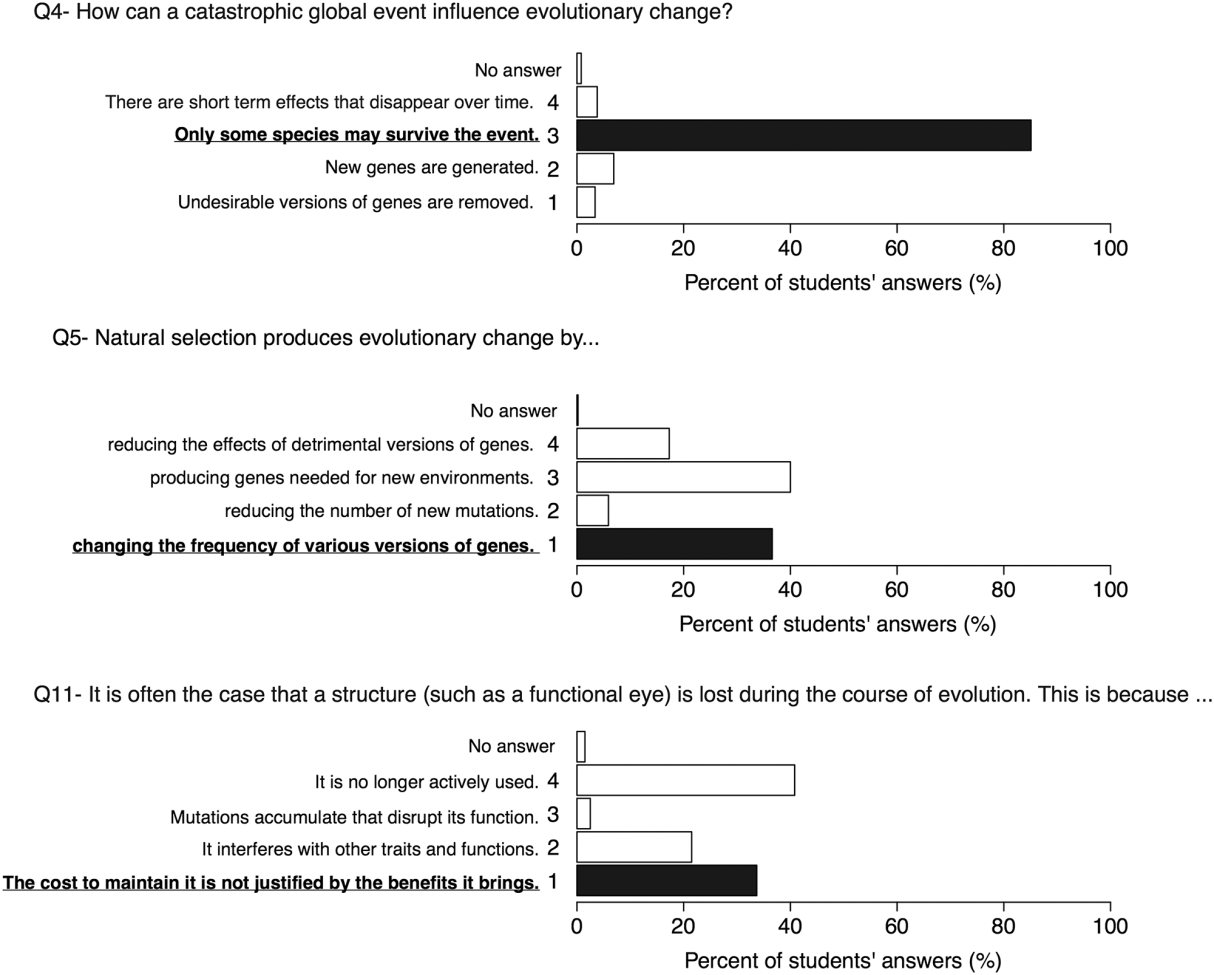
Results on questions 4, 5 and 11. On three out of four BCI questions related to evolutionary processes, many participants were attracted by distractors underlying misconceptions such as "needs as a rational for changes" or "use and disuse of an organ". The best answers are in bold.

Evolutionary processes influence all biological mechanisms and arise from the combination of mutations, non-adaptive events (genetic drift, founder and bottleneck effects, and gene linkage) and selective processes (natural, sexual, and social). Some BCI questions address students thinking about evolution. Question 4 asks: "How can a catastrophic global event influence evolutionary change?". Almost all participants have selected the best answer, "Only some species may survive the event". In contrast, on question 5, “Natural selection produces evolutionary change by…”, approximately 40% of Gymnasium students have selected the distractor, “producing genes needed for new environments” (an active, need-driven rather than selective process). In response to question 11 "It is often the case that a structure (such as a functional eye) is lost during the course of evolution. This is because…", roughly 34% of Gymnasium students were attracted by the answer: “It is no longer actively used” (Fig 1). The best answer, “The cost to maintain it is not justified by the benefits it brings” was selected by ~40% of the participants. These evolution-related questions reveal that many students share popular misconceptions such as "needs as a rational for change" and "use and disuse". Those purposeful (teleological) ideas can obstruct the development of an authentic understanding of evolutionary processes that are mainly based on random events [46–49].

### Molecular properties and functions (Q9, Q10, Q22)

**Fig 2:**
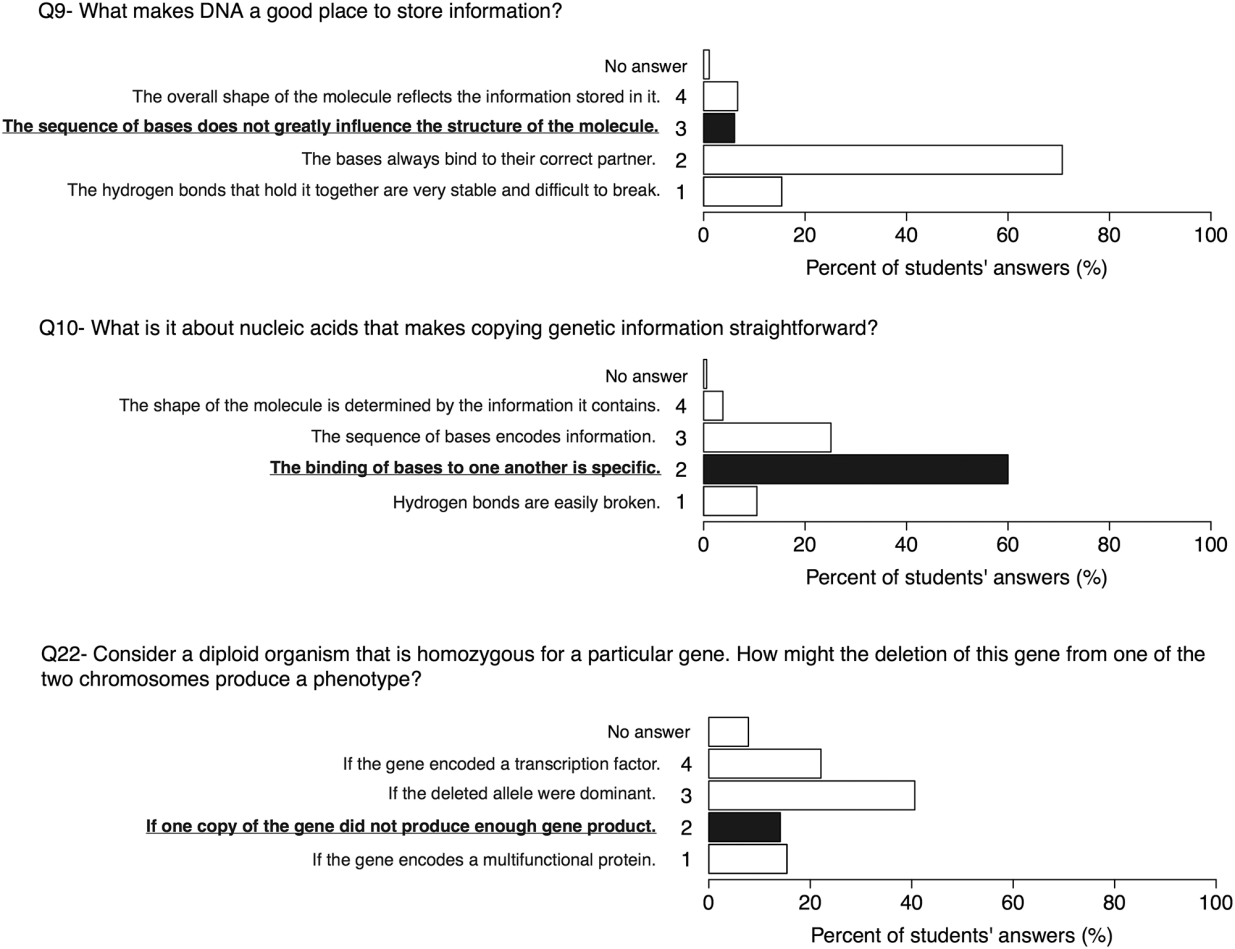
Results on questions 9, 10 and 22. Some misconceptions related to the information storage function of DNA and the interconnection between molecular properties and genetics are observed among many participants. The best answers are marked in bold.

The phenotypic effects of an allele must be explained by molecular mechanisms that include transcription and translation, polypeptide folding (and protein assembly) and molecular interaction, all of which are influenced by thermodynamic principles. Similarly, the function of DNA is closely related to its structure and molecular properties. A subset of BCI questions explore student thinking about structural and physical properties and biological functions of DNA. On question 9 (“What makes DNA a good place to store information?”), almost all participants selected the wrong answer: “The bases always bind to their correct partner”, a statement that correctly explains the basic mechanism of nuclear acid replication (addressed in question 10), but does directly relevant to the question of how information is stored in DNA. Few students selected the best answer: “The sequence of bases does not greatly influence the structure of the molecule”. The fact that the sequence of nucleotides in a DNA molecule does not greatly influence the molecule’s overall structure is central to its information storage role [50] and distinguishes DNA from both RNAs and proteins, the other molecules that carry information in a cell. Conversely, question 10 examines the properties of DNA that facilitate replication: "What is it about nucleic acids that makes copying genetic information straightforward?" The majority of participants selected the best answer ("The binding of bases to one another is specific"), suggesting that this aspect of DNA (nucleic acid) structure is well understood. Question 22 (“Consider a diploid organism that is homozygous for a particular gene. How might the deletion of this gene from one of the two chromosomes produce a phenotype?”) integrates concepts of genetics and molecular biology, i.e., how both act on the phenotype of an organism (Fig 2). Gymnasium students mostly selected the wrong response: “If the deleted allele were dominant” (~41%); what exactly students are thinking here is unclear. They may confuse the term “dominance” with a relative strength of an allele rather than considering it in terms of molecular mechanisms. Few students seemed to recognize that the number of genes influence the concentration of a gene product, which corresponds to the best answer: if one copy of the gene did not produce enough gene product" (~14%). Moreover, this question contains a number of common (in genetics and molecular biology) technical words, such as “homozygous”, “phenotype”, “gene product”, “allele”, or “transcription factor”; in their answers, many participants added question marks next to some terms. This question was also the highest no-answering question of the BCI. It is well recognized that students often struggle with genetic terminology [29,51]. It also indicates that students think in separate disciplines, e.g., genetics and molecular biology, and have difficulties to connect their knowledge in these fields.

That said, the answers to this question reveal that students interpret the term “dominance” to mean a struggle of alleles with each other—dominant alleles “winning” over recessive ones. As suggested by Klymkowsky et al. [20], dichotomous (black or white, or in this respect dominant/recessive) thinking cannot lead to an authentic comprehension of how alterations in molecular mechanisms (including gene expression and gene product functions) influence phenotypes. This misunderstanding appears to result from the common use of terms like dominant and recessive, without considering the molecular impact of mutations or allelic differences; the term “dominance” has been reported as an important misconception in teaching and understanding genetics [25,52]. In practice, the borders between dominant and recessive are not that sharp as one might deduce from the definition of these terms. For example, to explain phenomena such as incomplete dominance, incomplete penetrance, genetic background effects, and dominant-negative effects, one has to consider the underlying molecular events, such as gene expression and function or interaction of gene products [53].

### Genetics (Q13, Q14, Q19)

**Fig 3:**
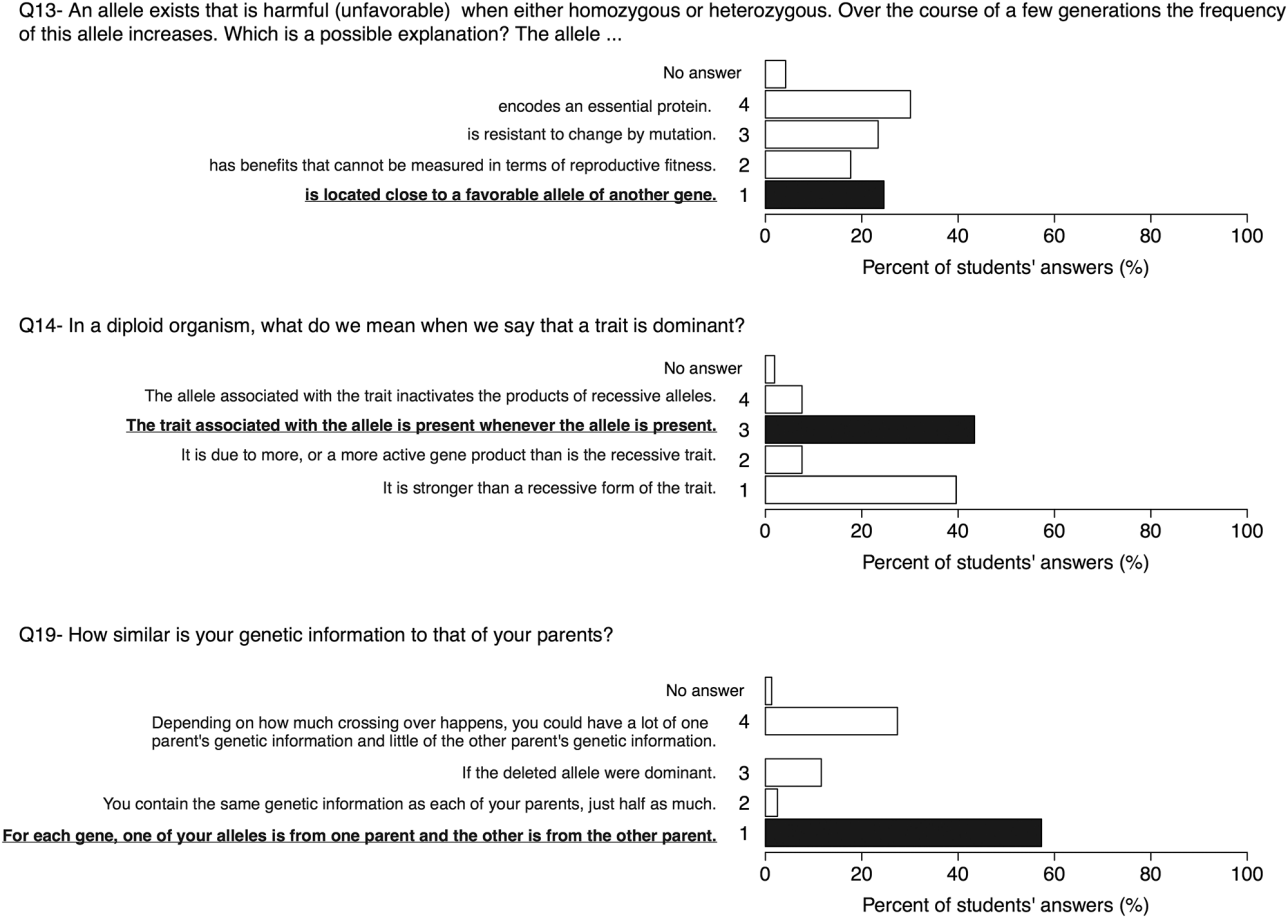
Results on questions 13, 14 and 19. On question 19, aspects of Mendel’s inheritance model seem to be understood. However, the restrictive dichotomous genetic thinking of "dominant" against "recessive" is prevalent among participants (as observed on question 22 (Fig 2)). Many participants are attracted by misconceptions not recognizing random mutations as an important factor in biological processes. The best answers are marked in bold.

Genetics (as distinct from molecular mechanisms) is a topic that is taught rather intensively at Swiss high schools. This is also represented in the respective BCI questions where many students were not attracted by common misconceptions on some genetic principles such as Mendel’s laws or the definition dominant or recessive traits. For question 14 (“In a diploid organism, what do we mean when we say that a trait is dominant?”), although most students (~43%) chose the correct definition for a dominant trait, almost as many students (~40%) prefer the answer “It is stronger than a recessive form of the trait” (Fig 3), conferring a powerful force to the dominant trait as also observed previously on question 22. On question 19 ("How similar is your genetic information to that of your parents?"), the majority of participants (between ~60%) selected the best answer, "For each gene, one of your alleles is from one parent and the other is from the other parent". The most popular distractor was: "Depending on how much crossing over happens, you could have a lot of one parent's genetic information and little of the other parent's genetic information", selected by ~25% of students. This shows a wrong—if at all present—concept of crossing over, a topic that is not taught extensively in high schools.

Question 13 is an interesting question because it tackles the concept of randomness, a process central to understand the effects of mutations and molecular behaviour. This question asks: “An allele exists that is harmful (unfavorable) when either homozygous or heterozygous. Over the course of a few generations the frequency of this allele increases. Which is a possible explanation? The allele…” (Fig 3). Students were mostly attracted by answers 3 “is resistant to change by mutation” (~23%) or 4 “encodes an essential protein” (~30%), believing that essential genes are resistant to selection and that alleles resistant to mutations are favored by selection, even if they are harmful. It may also point to students believing that mutations occur directional instead of randomly, a misconception shared by many students still at university level. Only the answer “is located close to a favorable allele of another gene” (selected by ~25% of students) provides a plausible mechanism for the increase in the frequency of the deleterious allele.

### Energetics and interactions (Q15, Q16, Q20)

**Fig 4:**
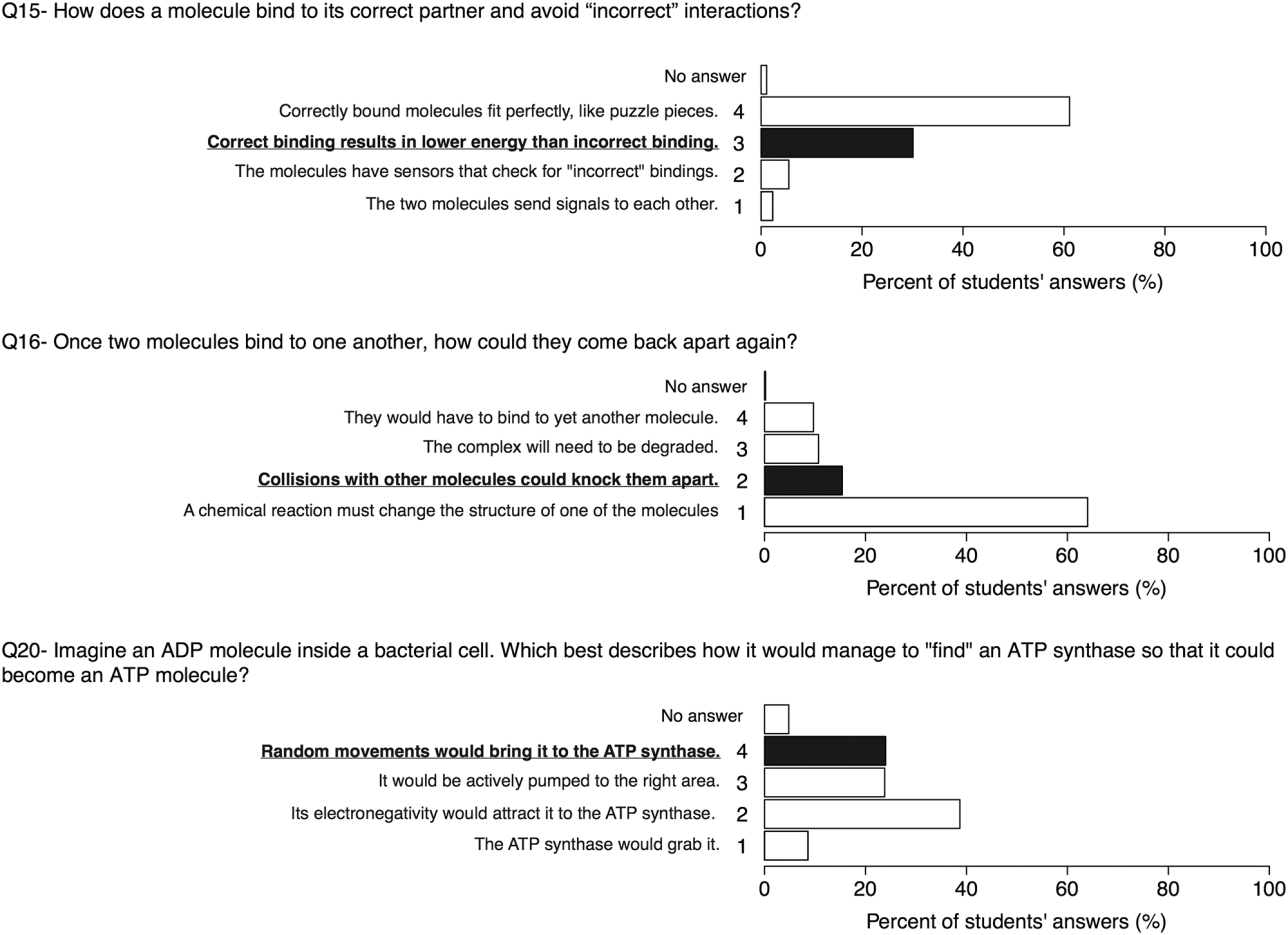
Results on questions 15, 16 and 20. Many participants were attracted by the oversimplifying analogy of puzzle pieces to explain molecular interactions and underestimated random diffusion and collisions as the main influence to spread and separate associated molecules.

To understand how molecules interact with each other is essential to consider their physiochemical properties. The BCI examines this topic by asking two questions related to the association (binding) and dissociation of molecules (Fig 4). Most students did not seem to have an accurate understanding of the energetics of molecular binding. The answers to question 15 (How does a molecule bind to its correct partner and avoid “incorrect” interactions?) revealed that many (~61%) Gymnasium students shared the misconception “Correctly bound molecules fit perfectly, like puzzle pieces”, a choice that is likely to be the result of teaching students through the use of a “key and lock” analogy, which makes evolutionary modification all but impossible [54] Interestingly, this answer is still chosen by 30% of students even after 2 years of biology studies at two Swiss universities [54], indicating that the physical and chemical foundations of molecular interactions are not well appreciated by students even after extended physics and chemistry instruction. In question 16, students were asked to explain the dissociation of molecules. The majority of participants (64%) were attracted by one of the answers involving an active (goal-directed) process (1-”A chemical reaction must change the structure of one of the molecules”). Only few participants (~15%) selected the best answer, “Collisions with other molecules could knock them apart”. Similar as in question 13, students neglect answers describing stochastic processes, indicating that randomness (stochasticity) is not considered to play a role in biological processes. Question 20 asks, “Imagine an ADP molecule inside a bacterial cell. Which describes how it would manage to “find” an ATP synthase so that it could become an ATP molecule?” (Fig 4). Sixty percent of the students chose answers describing active processes such “Its electronegativity would attract it to the ATP synthase” or “It would be actively pumped to the right area”, indicating that active processes such as pumping, electronegativity or grasping are more attractive answers than random movements based on thermodynamic effects to explain the displacement of molecules.

## Discussion

Since 2000, a growing number of assessments has become available to diagnose students’ biological understanding and to evaluate their misconceptions (see Table 1). In the current project, we aim at identifying specific areas where targeted instruction might be useful (or necessary) by using the Biological Concepts Instrument (BCI). The BCI was distributed to pre-university students (Gymnasium level) in different cantons of Switzerland to gain an overview of the instructional needs of these students. The results reveal several concepts that need to be deepened at the university level to promote biological literacy.

### What do the BCI results reveal?

The main advantage of the BCI is its large diversity of questions related to different biological concepts, which can be completed by students in only 30 minutes in class and thus provide a broad overview of their conceptual understanding in biology. Since the BCI was constructed on undergraduates' misconceptions, this study revealed important misunderstandings as enumerated below:

1. Many students, who underwent intensive instruction in Biology nonetheless, share popular misconceptions about evolution such as "needs as a rational for change" and "use and disuse".
2. The majority of students underestimated the role of randomness (stochasticity) in biological processes.
3. Students lack understanding of the physicochemical properties of molecules or have problems applying knowledge from physics and chemistry instruction to biological processes.

#### 1. Many students share popular misconceptions about evolution

Naive ideas such as "needs as a rational for change" and "use and disuse" are often reported as important misconceptions that harm to deeply understand evolution [46–49]. Bishop and Anderson [55] reported on how students’ thinking was different from the accepted biological theory by using multiple-choice questions and short-ended answers in a pre-post-test approach. One observation concerning the origin of new traits was that students failed to distinguish random changes in genetic material (by mutation or recombination) from natural selection (adaptation to environmental factors). Instead, students believe that environmental changes cause traits to be modified over time, i.e., the need for adaptation leads to direct genetic modifications in individuals that are inherited by the offspring. Using the Conceptual Inventory of Natural Selection (CINS), similar results were obtained by Anderson et al. [24]. For example, the CINS question 19 asks, "According to the theory of natural selection, where did the variations in body size in the three species of lizards most likely come from?", where only 33.7% of students have selected the correct answer: "Random genetic changes and sexual recombination both created new variations". The others were attracted by common misconceptions such as "the lizards need to change in order to survive (…)" or "the island environment caused genetic changes in the lizards". In their work using the Molecular Biology Capstone Assessment (MBCA), Couch et al. [34] found that 43% of students believe that organisms can induce specific mutations to intentionally avoid predation. These results are consistent with our finding that many students are attracted by common misconceptions such as "needs as a rational for change" and "use and disuse" to describe natural selection and other evolutionary processes. We know from the research on conceptual change that learners only fully adopt new scientific explanations if two conditions are fulfilled [56–58]. First, they must become dissatisfied with existing explanations. An authentic understanding of evolution requires consideration of stochastic events underlying evolutionary trajectories [59,60]. However, many students perceive evolution as a purposeful mechanism and consequently, as suggested by Kampourakis and Zogza [60], "instruction should focus on the role of chance in the evolutionary processes". Indeed, learners should be confronted with evidence for evolution they would not intuitively pay attention to. Instead of teaching evolution by discussing fossil studies and Darwin finches (which is how evolution theory is introduced in most text books and lectures), students should be confronted with examples that facilitate thinking of evolution on a population level, a prerequisite for an authentic understanding of evolution. For example, a session on evolution could be started by introducing evolution on bacteria, yeast or other “unfamiliar” organisms, which allows students to reconsider their teleologically-driven beliefs on how evolution works. Taking such an approach could also aid to fulfill the second condition required to adopt new scientific concepts, namely that the new concepts and explanations taught should be intelligible and plausible. We observe in this study that learner do not consider stochasticity in biological processes, because it interferes with their teleological beliefs and their concept of “exact science”. Examining evolution from a molecular angle would enable students to transfer their existing knowledge on stochastics and thermodynamics from chemistry, mathematics and physics courses to recognize the role of randomness in biological processes, such as mutation and molecular interactions.

#### 2. The majority of students underestimated randomness in biological processes

As considered above, most participants underestimated or dismiss the role of stochastic events as the driving motion of molecules and many other biological processes. Previous results showed that only ~49% of 2nd-year undergraduates studying biology at two distinguished Swiss universities have selected the best answer, “Random movements would bring it to the ATP synthase” in question 20 (Fig 4) [54]. Odom and Barrow [38] have revealed, through the use of the Diffusion and Osmosis Diagnostic Test (DODT), that many students are attracted by answers underlying an anthropomorphic view of matter such as "the molecules *want* to spread out" or "there are too many particles crowded into one area and therefor they move to an area with more room". In contrast, Couch et al. [34] found that ~74% of students correctly selected "true" for the statement 12 of the MBCA: "the ligand moves across this distance sometimes towards and sometimes away from the receptor" to describe how a signalling ligand can travel across a given distance to a receptor. However, of those students who selected "true" for the previous statement, the majority (78%) of them selected as true the contradictory statement: "the receptor senses the ligand and draws the ligand across this space". They appear to accept that molecular processes are controlled by directed processes rather than random collisions with other molecules. Metz [61] and Ziegler and Garfield [62] reported that the concept of stochasticity is hard to teach to students. According to Wilensky and Resnick [63], randomness is often referred to “as something that destroys order and interferes with goals”. Additionally, these authors suggested the term “deterministic mindset”, the teleological thinking that attempts to make sense of processes, i.e., by explaining diverse events based on outcomes (what they are “attempting” to achieve). According to the classification of misconceptions suggested by Chi [7], this type of misconception corresponds to a “missing scheme”, because the concept of stochasticity on a molecular level and emergent processes (such as the selection of appropriate aminoacyl-tRNAs by the mRNA-ribosome complex) are rarely explicitly taught. The BCI participants’ results demonstrated the teaching need to explicitly address this concept of stochasticity on all educational levels and in the context of a number of processes. This information can lead to elaborate further projects to deepen this under-estimation of randomness.

#### 3. Students lack understanding of the physicochemical properties of molecules or have problems applying knowledge from physics and chemistry instruction to biological processes

We observed some important misunderstandings of how DNA structure facilitates the storage of genetic information and how a failure to appreciate the dynamics of molecular interactions can be traced back to missing knowledge on the importance of physical and chemical properties of molecules. Previous results demonstrated that such misunderstanding is still found even after 2 years of undergraduate education at university [54]. Students’ understanding of how conformational and physicochemical properties of DNA and molecular movements are involved in molecules “finding” each other, interact and come apart (interaction specificities and stabilities / half-life) seems weak. Basic knowledge chunks, such as DNA structure, DNA replication, RNA synthesis (transcription), and polypeptide synthesis (RNA translation) are all based on stochastic interactions, which can only be understood based on the physicochemical properties of molecules and biological systems. These are ideas that are generally not clear for many students [20,64]. Often, students have weak abilities to apply cross-disciplinary thinking, mainly the result of the disciplinary silo teaching (not referring to processes and phenomena in other disciplines) [65,66]. Nagel and Lindsey [66] demonstrated that students do not automatically transfer knowledge from one discipline or domain to another, a process necessary to develop scientific literacy abilities. We can use such insights to reconsider course design. For example, Klymkowsky and colleagues [11] introduced changes into the design of an undergraduate introductory biology course to specifically addressing such misunderstandings.

### Initiatives for changes in a biology curriculum

An obvious approach to more efficient curriculum design is to first assess student needs by identifying areas of problematic understanding (known as “needs assessment” in management terminology) [21]. The results from this and other applications of the BCI [54] have stimulated an initiative to rethink the biology curriculum with respect to the educational needs students have when entering university. For example, the introductory biology course (“Grundlagen der Biologie 1A”) has been refocused to concentrate on key concepts of biological systems, from the chemical and physical properties of molecules and cells to genetics and gene expression. Evolutionary processes are taught in the second semester (“Grundlagen der Biologie 1B”). The introductory course is held partly as a “flipped classroom” in which students prepare part of the content at home, while the class hours are primarily spent with tutored exercises, group work, problem solving and discussions. This approach leaves the lecturer with more time to interact with students during instruction and get feedback about their actual understanding. To address the issue of stochasticity in biological processes, several exercises were designed that are discussed with students [67]. Further, the conceptual change of the students over the whole course of the semester is now evaluated in a pre/post-test approach. This allows lecturers to adapt their teaching accordingly and spend more time on issue that are less understood. Finally, to enhance the interdisciplinary thinking of students, the introductory physics course for biology students has been redesigned, focussing on the relevance of physical principles for biology. Both the lecture, the exercises and the supporting online material for this course integrate real examples on how physics laws impact on biology, with a focus on the macromolecular level (e.g., discussing the forces that operate during centrifugation of cells or the physical properties of molecular motors).

Recently, the Center for Active Learning (CAL) was created at the Department of Biology at ETH. A group of lecturers and educational experts work together to develop innovative learning and teaching activities in biology. For example, they generate online learning material and activities on which students receive timely information about their learning progress. The group is also involved in developing learning activities to promote discussions between students and lecturer in class. Here, they use the information revealed by the BCI to develop teaching and learning material that focus on molecular interactions, evolutionary principles and randomness throughout different lectures in the Biology curriculum. How these changes impact student understanding of biological processes is currently under study.

Finally, at ETH, a diploma for teaching at the Gymnasium level is offered. This programme can be completed after gaining a M.Sc. degree in sciences. The distribution of the BCI to Gymnasium students has provided us with important information about some important misconceptions held by students [54] and these insights are being incorporated into the teacher training program. In the ETH teaching diploma program, the course: “Specialized Biology Course with an Educational Focus: Teaching Diploma” emphasizes common misconceptions in biology and evolutionary principles. The students in this program are asked to create a teaching video in biology at the end of the course, in which they highlight the importance of evolution on a specific topic.

## Conclusions

Using the Biological Concepts Instrument, we show that many students were attracted by popular misconceptions on questions related to evolutionary processes, interactions and structures of molecules. In some cases, this appears to be due to failure to emphasize the relevance of knowledge from physics and chemistry instruction to biological contexts. The information gained from this survey helped and will further be used to adapt the current biology bachelor programme and the education of future Gymnasium biology teachers taking place at Swiss universities.

## Supporting information

S1 Text Supplemental methods and results. Description of the Biological Concepts Instrument (BCI). German version of the BCI. EVAMAR. Students’ performance comparisons. Fig A.: Percent of correct answers. Results on all BCI questions.
(PDF)

S1 Dataset The dataset contains all data of the present study. (PDF)
