# Supplementary material for: Diagnostic of students' misconceptions using the Biological Concepts Instrument (BCI): A method for conducting an educational needs assessment
Source: PLoS One. 2017 May 11;12(5):e0176906. doi: 10.1371/journal.pone.0176906 (PMC5426623; doi:10.1371/journal.pone.0176906)
Supplement: S1 Text — Description of the Biological Concepts Instrument (BCI). German version of the BCI. EVAMAR. Students’ performance comparisons. Fig A.: Percent of correct answers. Results on all BCI questions. (PDF) [file pone.0176906.s001.pdf]

Diagnostic of students' misconceptions using the Biological Concepts Instrument: A method for conducting an educational needs assessment

Annie Champagne Queloz[1]*, Michael W. Klymkowsky[2], Elsbeth Stern[3], Ernst Hafen[1], Katja Köhler[1]

1- Institute of Molecular Systems Biology, ETH Zürich

2- Molecular, Cellular, and Developmental Biology, University of Colorado Boulder

3- Institute for Educational Science, ETH Zürich

* Corresponding author

E-mail: champagne@imsb.biol.ethz.ch

Supporting Information

Materials and Methods

The Biological Concepts Instrument (BCI)

The Biological Concepts Instrument (BCI) was developed to reveal students' misconceptions in biology [1]. The original version is composed of 30 questions related to diverse concepts often taught at college and university level, topics that include evolutionary mechanisms, structure and function of molecules, molecular interactions, stochastic processes, genetics, energetics and experimental design. For the current project, 24 questions were selected to be completed in 25 minutes. Two biology didactic experts and one biology expert were consulted. They have recommended to modify or to remove questions 5, 14, 19, 23, 24, 28 of the original version of the BCI. The selection was mainly based on the presence of some ambiguities in the questions and the distractors and topics covered on those questions were not taught at the Gymnasium level.

Translated German version of the BCI

Fragen des BCI: Deutsch Version

Sie können nur eine Antwort pro Frage wählen.

1. Diffusion und Drift
Viele Zimmerpflanzen welken, wenn sie nicht genügend Wasser bekommen haben, richten sich aber nach dem Giessen schnell wieder auf. Diese Formveränderung kommt zustande, weil das Wasser....

A. mit den Zellwänden reagiert und diese versteift
B. als Energielieferant dient, um die Pflanze zu bewegen
C. die Salzkonzentration in der Pflanze verändert
D. in die Zellen eindringt und diese ausdehnt

2. Energetik und Interaktionen
Wie unterscheidet sich die Art der Energiegewinnung in Pflanzen und Tieren?

A. Tiere nutzen ATP, Pflanzen nicht
B. Pflanzen können Energie aus dem Sonnenlicht nutzen, Tiere nutzen chemische Energie
C. Pflanzen speichern Energie in Form von Zucker, Tiere nicht
D. Tiere können Zucker aus einfachen Molekülen (Bausteinen) herstellen, Pflanzen nicht

3. Energetik und Interaktionen
Wie unterscheidet sich die Art und Weise, wie Pflanzen und Tiere Energie nutzen?

A. Pflanzen brauchen Energie, um Moleküle herzustellen, Tiere können dies nicht
B. Tiere brauchen Energie, um Moleküle abzubauen, Pflanzen können dies nicht
C. Tiere benutzen Energie, um sich zu bewegen, Pflanzen können dies nicht
D. Pflanzen können Energie direkt verwenden, Tiere müssen sie erst freisetzen

4. Evolution
Wie kann eine weltweite Umweltkatastrophe die Evolution von Organismen beeinflussen?

A. Unerwünschte Varianten von Genen werden eliminiert
B. Neue Gene entstehen
C. Nur bestimmte Arten überleben den Vorfall
D. Es gibt nur kurzzeitige Effekte, die mit der Zeit wieder verschwinden

5. Evolution
Natürliche Selektion beeinflusst die Evolution von Organismen dadurch, dass...

A. sie die Häufigkeit von bestimmten Genvarianten verändert
B. sie die Zahl der neuen Mutationen verringert
C. sie Gene erzeugt, die die Anpassung an neue Umgebungen ermöglicht
D. sie den Einfluss von schädlichen Genvarianten verringert

6. Genetik
Beide Elternteile zeigen unterschiedliche Formen eines Merkmals. Ihre zahlreichen Nachkommen weisen alle die gleiche, neue Form des Merkmals auf. Die wahrscheinlichste Begründung hierfür wäre, dass...

A. beide Eltern mischerbig (heterozygot) für das Gen sind, das für das Merkmal verantwortlich ist
B. beide Eltern reinerbig (homozygot) für das Gen sind, das für das Merkmal verantwortlich ist
C. ein Elternteil mischerbig (heterozygot) und der andere reinerbig (homozygot) für das Gen ist, das für das Merkmal verantwortlich ist
D. Rekombination in einem oder beiden Elternteilen stattgefunden hat

7. Planung von Experimenten
In einem Experiment soll getestet werden, ob eine bestimmte Art der Akupunktur wirksam ist. Bei dieser Art der Akupunktur hat die Einstichstelle der Nadel einen Einfluss auf bestimmte Körperstellen. Als Teil des Experiments sollen einigen Versuchspersonen die Nadeln an der korrekten und anderen an der falschen Stelle eingestochen werden. Wozu dient das Einstechen in die falschen Stellen?

A. Es dient als negative Kontrolle
B. Es dient als positive Kontrolle
C. Es soll kontrolliert werden, ob die Person die Nadel spüren kann
D. Es soll kontrolliert werden, ob die Nadeln nötig sind

8. Planung von Experimenten
Damit dieses Experiment wissenschaftliche Aussagekraft hat, wäre es wichtig....

A. nur Personen zu testen, die an die Wirksamkeit von Akupunktur glauben
B. nur Personen zu testen, die keine vorgefertigte Meinung zur Wirksamkeit der Akupunktur haben
C. die Studie von Forschern entwickeln zu lassen, die an die Wirksamkeit dieser Form von Akupunktur glauben
D. zu untersuchen, ob das Einstechen der Nadeln an verschiedenen Stellen zu unterschiedlichen Ergebnissen führt

9. Struktur und Funktion von Molekülen
Die DNA ist gut als Informationsspeicher geeignet. Welche der folgenden Aussagen ist korrekt?

A. Die Wasserstoffbrückenbindungen, die sie zusammenhalten, sind sehr stabil
B. Die Basen binden immer an ihre passenden Partner
C. Die Basensequenz hat keinen besonderen Einfluss auf die Molekülstruktur
D. Die Molekülform spiegelt die gespeicherte Information wider

10. Struktur und Funktion von Molekülen
Welche Eigenschaft der Nukleinsäuren macht das Kopieren der genetischen Information so einfach?

A. Die Wasserstoffbrückenbindungen sind einfach aufzubrechen
B. Die Bindung der Basen zueinander ist komplementär
C. Die Basensequenz kodiert für Information
D. Die Form des Moleküls wird durch die enthaltene Information bestimmt

11. Evolution

Im Laufe der Evolution gehen oft bestimmte Strukturen (z.B. ein funktionierendes Auge) verloren, weil...

A. die Struktur nicht länger aktiv gebraucht wird
B. Mutationen sich anhäufen, die ihre Funktion zerstören
C. die Struktur andere Merkmale und Funktionen beeinträchtigt
D. die Kosten zur Aufrechterhaltung der Struktur höher sind als der Vorteil, den sie bringt

12. Struktur und Funktion von Molekülen
Um zu entscheiden, ob ein Molekül eine Membran durchdringen kann, muss man berücksichtigen,...

A. welche spezifischen Lipide in der Membran vorhanden sind
B. in welchem Masse das Molekül wasserlöslich ist
C. ob das Molekül aktiv von der Lipidschicht abgestossen wird
D. ob das Molekül für die Zelle schädlich ist

13. Genetik
Nehmen wir an, ein Allel eines Gens ist schädlich, wenn es heterozygot oder homozygot vorliegt. Innerhalb weniger Generationen erhöht sich die Häufigkeit des Allels. Weshalb?

A. Das Allel liegt in der Nähe eines vorteilhaften Allels eines anderen Gens
B. Das Allel hat andere Vorteile, die man nicht anhand des Fortpflanzungserfolgs messen kann
C. Das Allel ist resistent gegen Mutationen
D. Das Allel kodiert für ein lebenswichtiges Protein

14. Genetik
Was ist gemeint, wenn man von einem „dominanten Merkmal“ eines diploiden Organismus spricht?

A. Das Merkmal ist stärker als die rezessive Form des Merkmals
B. Das Merkmal entsteht aufgrund von mehr bzw. aktiverem Genprodukt als bei der rezessiven Form
C. Das Merkmal ist vorhanden, sobald das Allel dafür in mindestens einer Ausführung vorhanden ist
D. Das Allel für das entsprechende Merkmal inaktiviert die Genprodukte des rezessiven Allels

15. Energetik und Interaktionen
Wie kann sichergestellt werden, dass ein Molekül seinen richtigen Partner bindet und falsche Interaktionen vermieden werden?

A. Die beiden Bindungspartner senden Signale zueinander aus
B. Die Bindungspartner haben Sensoren, mit denen sie falsche Bindungen überprüfen
C. Durch die richtige Bindung wird ein niedrigerer Energiezustand erreicht als durch die falsche Bindung
D. Richtig gebundene Moleküle passen wie Puzzleteile perfekt zueinander

16. Energetik und Interaktionen
Wenn zwei Moleküle aneinander gebunden haben, wie können sie dann wieder getrennt werden?

A. Die Struktur eines der Moleküle muss durch eine chemische Reaktion verändert werden
B. Sie könnten durch Zusammenstöße mit anderen Molekülen getrennt werden
C. Der Komplex muss abgebaut werden
D. Sie müssen an ein weiteres Molekül binden

17. Struktur und Funktion von Molekülen
Lipide bilden Doppelschichten, weil...

A. sie keine Bindung mit Wassermolekülen eingehen können
B. sie nicht mit anderen Molekülen interagieren können
C. sie spezifisch an andere Lipidmoleküle binden können
D. Teile der Lipidmoleküle mit Wasser interagieren können

18. Genetik
Eine Mutation führt zu einem dominanten Merkmal. Was kann man über den Effekt der Mutation aussagen?

A. Die Mutation führt zu einem überaktiven Genprodukt
B. Die Mutation führt zu einem normalen Genprodukt, das sich jedoch mehr als üblich anreichert
C. Die Mutation führt zu einem Genprodukt mit einer neuen Funktion
D. Der Effekt der Mutation hängt von der Art des Genprodukts und der Mutation ab

19. Genetik
Wie ähnlich ist Ihre genetische Information der Ihrer Eltern?

A. Für jedes Gen besitzen Sie ein Allel der Mutter und eines des Vaters
B. Ihre Gene sind die gleichen wie diejenigen, die Ihre Eltern von deren Eltern geerbt haben
C. Sie besitzen die gleiche Information wie jeder Elternteil, nur halb so viel
D. Je nachdem, wieviel „crossing over" stattgefunden hat, besitzen Sie viel der genetischen Information eines und wenig des anderen Elternteils

20. Diffusion und Drift
Stellen Sie sich ein ADP-Molekül in einer Bakterienzelle vor. Wie könnte dieses Molekül zu einer ATP-Synthase gelangen, so dass es zu einem ATP Molekül ergänzt werden kann?

A. Die ATP-Synthase würde sich das ADP Molekül einfach greifen
B. Aufgrund seiner Elektronegativität würde es von der ATP-Synthase angezogen
C. Es würde aktiv an die richtige Stelle gepumpt werden
D. Das Molekül gelangt durch zufällige Bewegungen zur ATP-Synthase

21. Evolution
Sie verfolgen die Häufigkeit eines bestimmten Allels eines Gens in einer Population von asexuellen Organismen. Im Laufe der Zeit bemerken Sie, dass dieses Allel aus der Population verschwindet. Das Verschwinden beruht vermutlich auf...

A. genetischer Drift
B. dem Effekt des Gens auf den Fortpflanzungserfolg
C. der Mutation des Gens
D. der Tatsache, dass das Überleben zufällig ist

22. Struktur und Funktion von Molekülen
Stellen Sie sich einen Organismus vor, der homozygot für ein bestimmtes Gen ist. Wie könnte ein Phänotyp durch das Fehlen des Gens auf einem der homologen Chromosomen entstehen?

A. Wenn das Gen für ein multifunktionelles Protein kodiert
B. Wenn eine Kopie des Gens nicht ausreichend Genprodukt herstellen kann
C. Wenn das fehlende Allel dominant war
D. Wenn das Gen für einen Transkriptionsfaktor kodiert

23. Diffusion und Drift
Sexuelle Fortpflanzung führt in kleinen Populationen zu genetischer Drift, weil...

A. das Finden eines Männchens zufällig ist
B. nicht alle Allele von den Eltern an die Kinder weitergegeben werden
C. sie mit einem Anstieg der Mutationsrate verbunden ist
D. sie neue Kombinationen von Allelen zulässt

24. Diffusion und Drift
Was verbindet genetische Drift und die Diffusion von Molekülen?

A. Beides sind die Ergebnisse gerichteter Bewegungen
B. Beide beinhalten das Überwinden einer Barriere
C. Bei beiden spielen zufällige Ereignisse ohne Rücksicht auf das Endergebnis eine Rolle
D. Nichts. Genetische Drift ist zufällig, während Diffusion auf molekularer Ebene normalerweise gerichtet ist

EVAMAR

Two questions derived from the EVAMAR evaluation (questions 3 and 12) related to genetics replaced two BCI questions [2]. In 2001, a national evaluation of the educational reform of gymnasium studies (“Evaluation der Reform”, EVAMAR), introduced in 1995 in Switzerland, was done by Franz Eberle and his group at the University of Zürich (Eberle et al. 2011). The general aim was to evaluate if this reform offers the possibility to obtain sufficient knowledge (or maturity) for higher studies. More than 3800 students around Switzerland have done different tests about first language knowledge (German, French and Italian), mathematics and biology between May and July 2007. Questions in biology were written according to the

description of the specific knowledge students should have in biology at the end of the degree secondary II ("Schweizerische Maturitätskommission", 2008) and the content of the most popular biology books used in Switzerland. Here, those EVAMAR questions aimed at evaluating students' knowledge and their consistency with some standardized Swiss criteria.

Results

Students' Performance Comparisons

The significant differences between groups of participants are shown in Figure S1. There was no significant difference in the distribution of individual students' scores between the two cohorts (Kruskal-Wallis, $\chi^2(df = 1) = 0.153$, p-value = 0.696, alpha < 0.05) (Figure S1-A). The point in the semester when the BCI was administered did not affect students' BCI scores. More precisely, the performances of participants who have completed the BCI in June, after instruction, were not significantly higher than students who have completed the questionnaire earlier in the semester (essentially before instruction) (Figure S1-B and S1-C). A Wilcoxon Signed-Ranks Test indicated that only BCI mean scores of Group 9 (participated on May 12th, 2014) was statistically significantly (p-value = 0.021, alpha < 0.05) higher than Group 2 (participated on March 19th, 2014).

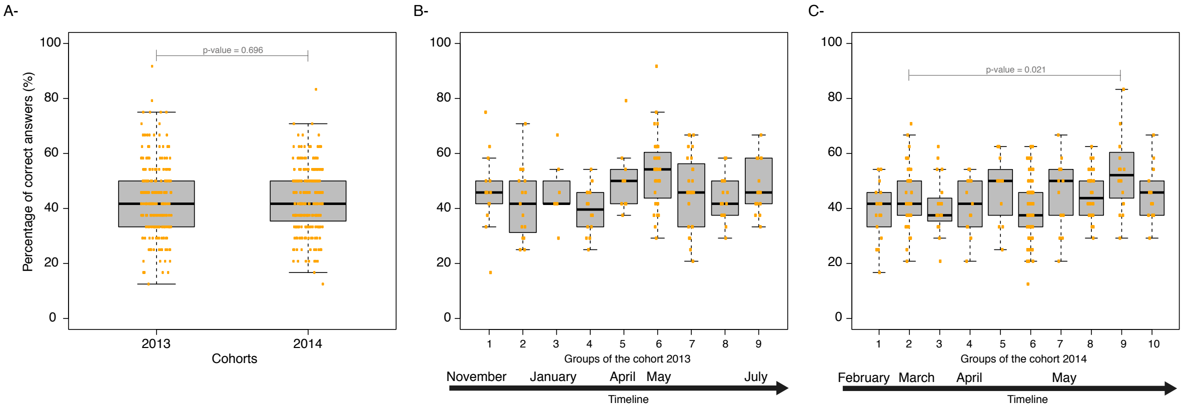

Fig. A. Percent of correct answers. A- Boxplot for the percent of correct answers on the BCI for two cohorts of Gymnasium students (211 participants in 2013 and 264 participants in 2014). B- and C- Boxplots for the percent of correct answers on the BCI for all groups of the cohort 2013 and the cohort 2014, respectively. The yellow dots are corresponding to an individual BCI score.

To evaluate whether students' knowledge is consistent with standardized Swiss criteria, we included two questions from the EVAMAR study (Eberle et al., 2011) [2] related to genetics (questions 3 and 12) into the BCI. The results for those questions correspond to those obtained by the research group of EVAMAR [2] (F. Eberle and M. Oepke personal communication). Consequently, the participants' knowledge was consistent with some standardized Swiss criteria. However, for confidentiality reasons, those questions cannot be shown in the current report.

Results on all BCI questions

1- Many types of house plants droop when they have not been watered and quickly "straighten up" after watering. The reason that they change shape after watering is because ...

Answer	Percentage answering (%)
a- Water reacts with, and stiffens, their cell walls.	2.9
b- Water is used to generate energy that moves the plant.	8.2
c- Water changes the concentration of salts within the plant.	17.3
**d- Water enters and expands their cells.**	**71.8**
NA- No answer	0.2

2- In which way are plants and animals different in how they obtain energy?

Answer	Percentage answering (%)
a- Animals use ATP; plants do not.	9.7
**b- Plants capture energy from sunlight; animals capture chemical energy.**	**76.8**
c- Plants store energy in sugar molecules	9.7
d- Animals can synthesize sugars from simpler molecules; plants cannot.	3.4
NA- No answer	0.4

3- EVAMAR question

4- How can a catastrophic global event influence evolutionary change?

Answers	Percentage answering (%)
a- Undesirable versions of genes are removed.	3.4
b- New genes are generated.	6.9
**c- Only some species may survive the event.**	**85.1**
d- There are short term effects that disappear over time.	3.8
NA- No answer	0.8

5- Natural selection produces evolutionary change by...

Answers	Percentage answering (%)
**a- changing the frequency of various versions of genes.**	**36.6**
b- reducing the number of new mutations.	5.9
c- producing genes needed for new environments.	40.0
d- reducing the effects of detrimental versions of genes.	17.3
NA- No answer	0.2

6- If two parents display distinct forms of a trait and all their offspring (of which there are hundreds) display the same new form of the trait, you would be justified in concluding that ...

Answers	Percentage answering (%)
a- both parents were heterozygous for the gene that controls the trait.	21.3
**b- both parents were homozygous for the gene that controls the trait.**	39.4
c- one parent was heterozygous, the other was homozygous for the gene that controls the trait.	29.5
d- a recombination event has occurred in one or both parents.	8.8
NA- No answer	1.1

7- You are doing experiments to test whether a specific type of acupuncture works. This type of acupuncture holds that specific needle insertion points influence specific parts of the body. As part of your experimental design, you randomize your treatments so that some people get acupuncture needles inserted into the "correct" sites and others into "incorrect" sites. What is the point of inserting needles into incorrect places?

Answers	Percentage answering (%)
**a- It serves as a negative control.**	**72.4**
b- It serves as a positive control.	12.0
c- It controls for whether the person can feel the needle.	3.6
d- It controls for whether needles are necessary	11.2
NA- No answer	0.8

8- As part of your experiments on the scientific validity of this particular type of acupuncture, it would be important to ...

Answers	Percentage answering (%)
a- test only people who believe in acupuncture.	2.3
b- test only people without opinions, pro or con, about acupuncture.	30.9
c- have the study performed by researchers who believe in this form of acupuncture.	0.6
**d- determine whether placing needles in different places produces different results.**	**65.9**
NA- No answer	0.2

9-What makes DNA a good place to store information?

Answers	Percentage answering (%)
a- The hydrogen bonds that hold it together are very stable and difficult to break.	15.4
b- The bases always bind to their correct partner.	70.7
**c- The sequence of bases does not greatly influence the structure of the molecule.**	**6.1**
d- The overall shape of the molecule reflects the information stored in it.	6.7
NA- No answer	1.1

10- What is it about nucleic acids that makes copying genetic information straightforward?

Answers	Percentage answering (%)
a- Hydrogen bonds are easily broken.	10.5
**b- The binding of bases to one another is specific.**	**60.0**
c- The sequence of bases encodes information.	25.1
d- The shape of the molecule is determined by the information it contains.	3.8
NA- No answer	0.6

11- It is often the case that a structure (such as a functional eye) is lost during the course of evolution. This is because ...

Answers	Percentage answering (%)
a- It is no longer actively used.	33.7
b- Mutations accumulate that disrupt its function.	21.5
c- It interferes with other traits and functions.	2.5
**d- The cost to maintain it is not justified by the benefits it brings.**	**40.8**
NA- No answer	1.5

12- EVAMAR question

13- An allele exists that is harmful (unfavorable) when either homozygous or heterozygous. Over the course of a few generations the frequency of this allele increases. Which is a possible explanation? The allele ...

Answers	Percentage answering (%)
**a- is located close to a favorable allele of another gene.**	**24.6**
b- has benefits that cannot be measured in terms of reproductive fitness.	17.7
c- is resistant to change by mutation.	23.4
d- encodes an essential protein.	30.1
NA- No answer	4.2

14- In a diploid organism, what do we mean when we say that a trait is dominant?

Answers	Percentage answering (%)
a- It is stronger than a recessive form of the trait.	39.6
b- It is due to more, or a more active gene product than is the recessive trait.	7.6
**c- The trait associated with the allele is present whenever the allele is present.**	**43.4**
d- The allele associated with the trait inactivates the products of recessive alleles.	7.6
NA- No answer	1.9

15- How does a molecule bind to its correct partner and avoid “incorrect” interactions?

Answers	Percentage answering (%)
a- The two molecules send signals to each other.	2.3
b- The molecules have sensors that check for "incorrect" bindings.	5.5
**c- Correct binding results in lower energy than incorrect binding.**	**30.1**
d- Correctly bound molecules fit perfectly, like puzzle pieces.	61.1
NA- No answer	1.1

16- Once two molecules bind to one another, how could they come back apart again?

Answers	Percentage answering (%)
a- A chemical reaction must change the structure of one of the molecules.	64.0
**b- Collisions with other molecules could knock them apart.**	**15.4**
c- The complex will need to be degraded.	10.7
d- They would have to bind to yet another molecule.	9.7
NA- No answer	0.2

17- Lipids can form structures like micelles and bilayers because of ...

Answers	Percentage answering (%)
a- their inability to bond with water molecules.	35.6
b- their inability to interact with other molecules.	4.2
c- their ability to bind specifically to other lipid molecules.	15.8
**d- the ability of parts of lipid molecules to interact strongly with water.**	**42.3**
NA- No answer	2.1

18- A mutation leads to a dominant trait; what can you conclude about the mutation's effect?

Answers	Percentage answering (%)
a- It results in an overactive gene product.	8.4

b- It results in a normal gene product that accumulates to higher levels than normal.	15.2
c- It results in a gene product with a new function.	25.7
**d- It depends upon the nature of the gene product and the mutation.**	**47.6**
NA- No answer	3.2

19- How similar is your genetic information to that of your parents?

Answers	Percentage answering (%)
**a- For each gene, one of your alleles is from one parent and the other is from the other parent.**	**57.3**
b- You have a set of genes similar to those your parents inherited from their parents.	2.5
c- You contain the same genetic information as each of your parents, just half as much.	11.6
d- Depending on how much crossing over happens, you could have a lot of one parent's genetic information and little of the other parent's genetic information.	27.4
NA- No answer	1.3

20- Imagine an ADP molecule inside a bacterial cell. Which best describes how it would manage to "find" an ATP synthase so that it could become an ATP molecule?

Answers	Percentage answering (%)
a- The ATP synthase would grab it.	8.6
b- Its electronegativity would attract it to the ATP synthase.	38.7
c- It would be actively pumped to the right area.	23.8
**d- Random movements would bring it to the ATP synthase.**	**24.0**
NA- No answer	4.8

21- You follow the frequency of a particular version of a gene in a population of asexual organisms. Over time, you find that this version of the gene disappears from the population. Its disappearance is presumably due to ...

Answers	Percentage answering (%)
a- genetic drift.	28.6
**b- its effects on reproductive success.**	**35.6**
c- its mutation.	27.2
d- the randomness of survival.	6.5
NA- No answer	2.1

22- Consider a diploid organism that is homozygous for a particular gene. How might the deletion of this gene from one of the two chromosomes produce a phenotype?

Answers	Percentage answering (%)
a- If the gene encodes a multifunctional protein.	15.4

**b- If one copy of the gene did not produce enough gene product.**	**14.1**
c- If the deleted allele was dominant.	40.6
d- If the gene encoded a transcription factor.	22.1
NA- No answer	7.8

23- Sexual reproduction leads to genetic drift because ...

Answers	Percentage answering (%)
a- there is randomness associated with finding a mate.	5.5
**b- not all alleles are passed from parent to offspring.**	**19.8**
c- it is associated with an increase in mutation rate.	24.0
d- it produces new combinations of alleles.	46.5
NA- No answer	4.2

24- How is genetic drift like molecular diffusion?

Answers	Percentage answering (%)
a- Both are the result of directed movements.	14.1
b- Both involve passing through a barrier.	12.2
**c- Both involve random events without regard to ultimate outcome.**	**24.6**
d- They are not alike. Genetic drift is random; diffusion typically has a direction.	45.1
NA- No answer	4.0

References

1. Klymkowsky MW, Underwood SM, Garvin-Doxas K. Biological Concepts Instrument (BCI): A diagnostic tool for revealing student thinking. arXiv.org. 2010.

2. Eberle F, Gehrer K, Jaggi B, Kottonau J, Oepke M, Pflüger M. Evaluation de la réforme de la maturité 1995 (EVAMAR). Secrétariat d'Etat à l'éducation et à la recherche SER CS, editor. 2011 Dec 30;1–424.
