# Supplementary material for: Diagnostic of students' misconceptions using the Biological Concepts Instrument (BCI): A method for conducting an educational needs assessment
Source: PLoS One. 2017 May 11;12(5):e0176906. doi: 10.1371/journal.pone.0176906 (PMC5426623; doi:10.1371/journal.pone.0176906)
Supplement: S1 Dataset — (PDF) [file pone.0176906.s002.pdf]

Table 1: Answer choices of students (cohorts 2013 and 2014) on all BCI questions

Group	Student	Score	Q1	Q2	Q3	Q4	Q5	Q6	Q7	Q8	Q9	Q10	Q11	Q12	Q13	Q14	Q15	Q16	Q17	Q18	Q19	Q20	Q21	Q22	Q23	Q24
2013_1	121245	12	4	2	4	3	1	3	1	2	1	3	4	3	2	3	4	2	3	3	1	3	1	1	2	1
2013_1	260857	11	4	2	4	3	1	3	1	2	2	2	1	2	0	3	2	1	3	4	1	0	2	1	1	4
2013_1	070565	4	4	1	4	3	3	1	4	2	2	3	2	1	2	1	4	3	1	4	4	3	3	3	4	4
2013_1	260960	9	3	2	4	3	3	2	1	2	2	2	1	2	1	1	4	1	4	3	1	2	1	3	4	2
2013_1	060169	6	2	2	4	3	3	2	4	4	2	2	1	2	4	4	4	1	1	2	3	2	3	3	1	4
2013_1	100264	10	3	2	4	3	4	2	1	4	2	4	2	2	3	3	4	1	1	4	1	3	3	3	2	4
2013_1	270362	6	1	2	4	3	4	0	4	2	2	3	4	1	4	1	4	1	1	0	1	0	2	0	0	0
2013_1	170962	12	4	2	4	3	3	3	1	4	2	2	1	3	2	2	3	1	1	4	1	3	2	3	0	0
2013_1	100262	9	4	2	4	3	3	2	1	4	2	2	4	2	4	4	4	1	1	3	3	3	3	4	3	2
2013_1	061156	13	4	2	4	3	2	2	1	4	0	2	1	4	1	4	4	4	4	4	1	4	3	4	4	4
2013_1	150858	6	2	2	2	3	1	3	3	2	2	3	1	2	3	1	1	1	4	4	1	3	1	4	4	4
2013_1	270461	14	3	2	4	3	3	1	1	4	2	2	4	3	2	3	3	1	4	4	1	1	1	0	4	3
2013_1	071254	9	4	2	4	3	3	3	1	4	1	3	4	2	3	1	4	1	3	4	3	4	4	4	4	1
2013_1	240260	11	4	2	4	3	3	3	2	4	2	2	4	3	1	3	4	1	3	4	0	3	1	0	4	4
2013_1	261167	9	4	2	4	3	1	3	3	4	2	3	1	2	1	1	4	2	1	4	4	3	3	3	1	4
2013_1	230254	5	3	4	2	3	1	1	1	4	2	3	1	2	4	3	4	1	2	3	4	2	4	4	3	4
2013_1	050162	11	4	2	4	3	4	1	1	4	2	1	1	2	4	1	3	1	1	4	1	4	2	3	4	4
2013_1	260760	7	4	1	2	3	3	1	1	2	2	2	1	2	4	1	4	2	1	3	1	4	3	1	4	4
2013_1	030361	10	4	2	4	3	3	3	1	4	3	3	1	3	2	1	4	1	1	4	1	3	3	1	4	4
2013_1	231163	13	4	2	4	3	1	4	1	4	1	2	4	3	4	3	4	1	1	4	1	2	4	3	4	4
2013_1	040766	8	4	1	4	3	3	3	1	4	2	3	1	2	4	3	4	1	1	4	4	2	2	3	4	4
2013_1	120263	11	4	4	2	3	1	3	1	4	2	3	4	3	1	1	3	1	1	2	1	3	4	4	2	1
2013_1	250964	10	4	2	4	3	3	2	1	4	4	3	1	2	2	1	4	1	4	4	3	2	2	1	3	2
2013_1	191159	11	1	2	4	3	1	2	1	4	2	2	1	3	4	3	4	3	4	2	3	2	3	1	1	4
2013_1	150766	10	1	2	4	1	3	1	1	4	1	2	4	4	3	1	4	2	4	4	1	3	3	1	4	1
2013_1	140468	6	0	2	2	3	1	3	1	0	0	3	0	0	0	1	3	2	1	1	4	3	0	3	3	4
2013_1	160968	8	3	2	4	3	3	3	1	4	2	3	1	2	4	1	4	1	1	4	4	4	3	4	2	1
2013_1	060761	9	3	2	4	3	3	2	1	4	2	1	1	2	2	3	4	3	1	1	4	4	3	3	2	1
2013_1	150566	18	4	2	4	3	1	2	1	4	2	2	4	3	2	3	4	1	1	4	1	4	2	3	2	3
2013_1	080552	16	4	2	4	3	1	2	1	4	2	2	1	2	4	4	3	2	1	4	1	3	2	3	2	3
2013_1	010451	7	1	2	2	3	3	2	4	2	1	3	2	2	4	2	2	3	1	3	1	4	2	2	3	1
2013_1	230755	13	4	2	4	3	1	2	1	4	2	2	1	2	4	1	2	1	2	4	1	2	2	4	2	4
2013_1	120471	9	4	2	4	3	3	2	2	1	2	3	2	1	4	1	1	1	4	4	4	3	3	3	2	3
2013_1	050664	8	2	2	2	3	3	3	2	3	4	3	4	1	4	2	3	1	4	3	1	4	3	4	2	4
2013_1	171262	7	4	1	4	1	3	0	3	4	1	3	4	2	0	0	0	0	1	4	1	0	2	0	3	0
2013_1	241262	14	4	2	2	3	1	2	1	4	4	2	4	2	1	4	2	3	4	4	4	3	2	3	4	3
2013_1	010264	12	4	2	4	3	1	1	1	4	2	2	4	4	2	1	4	1	4	1	1	2	2	4	4	4
2013_2	230469	9	1	2	4	3	1	1	2	4	2	3	1	2	1	3	4	4	1	3	1	1	1	2	4	1
2013_2	170268	9	4	2	4	1	2	3	2	4	2	3	4	2	2	1	3	3	3	3	1	4	1	3	3	3
2013_2	020666	11	4	2	4	3	1	3	4	4	2	3	4	4	4	1	3	2	1	2	1	2	2	4	1	2
2013_2	061160	14	4	3	4	3	1	2	2	2	3	2	4	3	1	4	4	1	4	4	1	4	1	3	4	1
2013_2	211263	18	4	3	4	3	1	2	1	4	2	2	4	3	1	3	4	1	4	4	1	4	3	2	4	3
2013_2	080765	5	3	2	4	3	3	1	2	4	1	2	2	2	3	1	2	1	1	3	3	2	1	3	3	2
2013_2	040761	8	2	2	4	3	4	2	4	4	2	0	1	2	3	2	4	3	1	4	1	3	3	2	3	0
2013_2	130163	12	4	2	2	3	4	3	4	4	2	2	4	2	1	3	3	3	4	3	4	3	1	2	3	3
2013_2	101263	16	4	2	4	3	1	3	1	4	1	2	4	2	1	3	3	3	3	2	1	2	2	2	2	4
2013_2	310560	5	1	2	2	1	3	2	4	4	1	3	1	2	3	1	4	2	4	3	4	2	3	3	0	0
2013_2	011068	10	3	2	4	3	1	1	1	4	3	3	4	2	3	1	3	1	1	4	4	3	1	4	4	2
2013_2	270961	7	2	2	4	3	3	1	4	4	1	3	4	2	4	1	4	1	1	4	1	3	1	4	3	2
2013_2	180460	11	2	2	4	3	3	3	4	4	2	2	4	2	2	3	4	1	3	4	1	4	2	3	4	2
2013_2	281063	8	2	2	2	2	3	2	1	4	2	3	4	2	3	1	4	2	4	3	4	4	3	3	0	0
2013_3	160294	18	4	2	4	3	1	2	1	4	1	2	2	3	2	3	3	1	4	4	1	2	1	2	2	3
2013_3	120662	13	4	2	4	3	1	1	0	2	2	2	4	3	2	3	3	1	4	4	3	4	1	3	1	1
2013_3	100658	12	4	2	4	3	1	3	1	4	2	3	2	3	3	2	3	1	2	4	1	0	3	3	2	4

2013_3	120254	9	4	1	4	3	1	3	1	2	1	2	1	3	0	3	0	1	0	0	0	0	1	0	2	0
2013_3	040569	12	4	2	4	3	1	2	4	2	4	2	1	2	4	3	4	3	3	4	3	2	2	2	4	3
2013_3	160369	10	4	1	4	3	3	3	1	2	2	2	4	2	4	1	4	1	2	4	1	2	2	4	4	3
2013_3	120264	19	4	2	4	3	1	2	1	4	1	2	2	3	2	3	3	2	4	3	1	4	1	2	2	3
2013_3	280165	14	2	2	4	3	1	2	1	4	2	2	1	3	2	3	3	1	0	4	1	0	1	0	1	3
2013_3	010259	10	4	2	4	3	3	2	0	2	1	2	1	3	0	3	4	1	0	4	1	0	1	0	4	0
2013_3	020271	12	4	2	1	3	3	2	1	4	2	3	1	0	2	3	4	2	4	3	1	0	2	0	1	3
2013_3	160267	13	4	2	4	3	1	3	1	4	2	2	1	0	2	3	3	2	4	0	1	0	1	0	4	1
2013_3	180958	10	3	2	2	3	1	0	1	4	1	2	2	4	4	1	3	1	1	4	1	0	2	4	3	4
2013_3	090956	9	4	1	2	3	1	4	3	4	2	3	4	3	4	1	4	1	1	2	1	4	1	1	2	4
2013_4	020463	13	4	2	4	3	1	3	1	4	2	2	2	3	1	3	4	3	1	2	1	2	2	4	4	1
2013_4	110962	13	4	2	4	3	3	4	1	4	2	2	2	3	1	3	4	1	4	4	4	2	2	4	4	2
2013_4	290653	13	4	2	4	3	3	2	1	2	2	2	4	3	1	1	3	1	1	3	1	3	2	4	4	4
2013_4	000000	15	4	2	4	3	1	3	1	4	2	3	4	3	1	3	4	1	4	4	4	4	2	3	4	4
2013_4	241163	9	2	3	4	3	3	2	1	4	2	2	4	3	2	1	4	1	1	4	4	3	1	4	4	1
2013_4	190567	9	2	2	4	3	3	3	1	4	2	2	2	2	4	1	4	1	4	4	3	3	2	1	4	4
2013_4	100764	12	4	3	4	3	3	2	4	4	3	2	1	3	1	1	4	1	1	4	4	3	2	3	2	4
2013_4	120567	22	4	2	4	3	1	2	1	2	3	2	4	3	1	3	3	2	4	3	1	4	2	2	2	3
2013_4	240961	14	4	2	4	3	4	3	1	4	2	2	4	3	1	3	4	1	3	4	1	3	1	2	4	4
2013_4	240961	14	4	2	4	3	4	3	1	2	2	2	4	3	1	3	3	1	1	4	1	2	1	1	4	3
2013_4	110263	15	4	2	4	3	3	3	1	4	2	2	4	3	1	3	3	1	3	4	1	3	2	4	4	4
2013_4	150561	12	4	2	2	3	3	2	1	4	2	2	2	3	1	3	3	1	1	2	1	3	3	4	4	4
2013_4	021266	17	3	2	4	3	1	2	1	2	2	2	2	3	1	3	3	4	1	4	1	4	2	2	4	3
2013_4	260264	12	4	2	4	3	2	1	1	4	2	2	3	1	2	3	4	1	4	3	1	1	2	1	4	3
2013_4	020764	17	4	2	4	3	1	2	1	2	2	3	4	3	2	3	3	1	4	4	1	2	2	2	2	1
2013_4	111260	12	4	2	4	3	1	4	1	4	2	1	4	3	2	1	4	1	3	4	1	4	1	1	3	1
2013_4	071257	10	3	2	4	0	4	3	1	4	4	2	4	3	2	0	3	4	1	4	4	4	1	4	4	4
2013_4	020964	13	4	2	4	4	4	3	1	4	2	1	4	3	1	3	3	4	4	2	1	4	3	3	3	4
2013_4	040961	12	4	2	4	2	3	2	1	4	4	2	4	2	1	4	4	1	4	4	1	0	3	3	4	1
2013_4	040457	15	4	2	4	3	1	2	1	4	2	2	2	3	2	3	4	2	1	4	1	3	1	0	2	4
2013_4	060672	18	4	2	4	3	2	2	1	4	3	3	4	3	2	3	3	4	4	4	1	4	1	1	2	3
2013_4	310156	13	4	2	3	3	4	1	1	2	3	2	4	2	4	3	3	1	4	4	4	3	2	3	1	3
2013_4	230456	13	4	2	4	3	3	2	1	4	2	2	4	2	1	3	2	1	4	2	1	2	1	3	4	4
2013_4	160763	13	4	2	2	3	1	1	1	4	1	2	4	1	1	3	1	1	4	4	1	3	1	4	4	4
2013_4	010965	9	2	2	4	2	1	3	1	4	2	2	2	3	4	1	4	1	1	4	1	3	1	3	3	1
2013_4	210194	11	4	2	3	3	3	2	1	4	2	3	1	2	1	1	4	3	4	1	1	3	2	2	4	4
2013_4	070961	10	4	2	4	3	3	3	1	2	2	2	4	3	4	3	4	1	1	4	4	3	3	1	4	4
2013_4	310566	10	4	2	2	3	3	1	1	4	2	3	4	0	1	3	4	1	1	4	4	1	2	0	3	4
2013_4	210164	8	4	2	2	3	3	1	1	4	2	3	1	2	4	3	4	1	4	3	4	2	2	0	3	4
2013_4	100167	7	3	2	2	4	1	2	2	4	3	1	2	2	4	1	4	1	4	3	1	3	1	3	4	4
2013_4	060566	14	4	2	2	3	1	1	1	4	2	2	4	3	1	3	4	1	4	4	4	4	3	1	4	4
2013_4	010260	15	4	2	4	3	4	2	1	4	2	2	1	3	1	3	4	4	4	1	1	4	2	3	3	1
2013_5	271167	13	2	2	4	3	4	2	1	3	2	2	4	3	3	3	4	3	4	2	2	4	2	1	2	1
2013_5	030759	12	3	2	4	3	1	1	1	2	3	2	4	3	1	2	3	1	1	2	3	3	3	4	2	2
2013_5	190370	11	3	2	4	3	4	2	1	4	2	2	4	3	2	3	4	1	3	2	3	2	2	1	4	4
2013_5	250561	15	4	2	4	3	1	1	1	4	1	2	4	3	4	3	4	2	4	4	3	4	1	3	4	4
2013_5	060563	16	4	2	2	3	1	2	1	4	2	2	4	3	4	3	3	3	1	4	4	4	2	1	4	3
2013_5	180257	5	2	3	2	3	1	3	4	2	2	2	1	3	3	4	2	4	4	3	3	3	3	3	4	4
2013_5	160264	11	4	2	2	3	2	3	1	2	1	2	2	3	4	3	4	3	4	4	1	4	1	1	4	4
2013_5	050163	8	4	2	4	1	3	1	1	2	1	3	4	3	2	3	4	1	1	4	3	3	1	4	4	4
2013_5	130457	7	4	1	2	3	4	4	4	4	3	2	2	1	4	3	4	1	3	4	4	2	1	4	4	4
2013_5	040454	12	3	1	4	3	1	2	1	4	1	3	1	3	4	3	4	1	4	4	1	3	1	3	2	2
2013_5	220668	10	4	4	4	3	1	3	1	2	2	2	4	3	2	4	2	1	4	3	1	2	1	4	4	4
2013_5	120165	11	4	2	2	3	1	4	1	1	2	2	4	2	4	3	4	1	4	4	4	4	1	3	3	4
2013_5	310158	14	3	2	4	3	4	2	1	2	3	2	4	2	2	3	4	1	4	4	1	4	4	3	2	1
2013_5	150256	8	4	3	2	3	4	3	4	2	1	2	4	2	2	3	4	1	1	4	1	3	2	3	4	1

2013_5	120864	11	2	2	2	3	1	1	1	4	4	2	4	2	3	3	4	1	4	4	1	2	3	3	4	4
2013_5	210467	14	4	2	2	3	1	1	1	4	1	2	4	3	2	3	4	1	3	4	1	2	2	3	2	4
2013_5	151163	11	4	2	2	3	1	2	1	4	1	3	2	3	2	3	3	4	4	2	3	2	4	3	4	4
2013_5	301000	8	4	4	4	3	4	3	4	2	2	3	4	2	1	3	4	2	1	2	3	2	1	1	4	3
2013_5	120266	11	3	2	2	3	1	2	1	4	2	2	4	4	2	1	4	3	4	4	4	3	2	3	4	4
2013_5	100369	7	3	2	4	3	1	2	3	4	2	3	2	4	3	1	3	1	1	1	3	3	1	3	4	4
2013_5	250664	11	2	2	2	3	1	1	1	2	3	3	4	2	1	3	4	1	4	4	1	3	1	3	1	4
2013_6	221157	13	4	4	4	3	1	4	1	4	2	2	4	3	2	3	1	1	4	4	1	2	4	3	4	4
2013_6	180768	15	4	1	2	3	3	2	1	4	2	2	4	3	4	3	3	2	1	4	1	3	2	3	4	3
2013_6	180266	16	4	2	4	3	1	2	1	2	3	2	2	3	4	3	4	1	4	4	1	4	1	3	4	3
2013_6	160563	11	4	2	4	3	1	1	2	4	2	3	1	4	3	1	4	1	4	4	1	4	1	3	4	3
2013_6	111059	7	3	2	3	1	3	1	1	4	2	3	2	3	3	1	4	1	4	2	1	1	2	4	4	4
2013_6	101159	6	4	2	2	3	4	1	0	2	2	3	4	2	4	3	4	3	1	4	4	3	3	3	3	4
2013_6	050559	16	4	2	1	3	4	4	1	4	2	1	4	3	1	3	3	1	4	3	1	4	2	2	3	3
2013_7	081153	11	4	2	4	3	1	1	1	2	2	1	4	3	4	3	4	1	1	3	1	1	3	4	2	4
2013_7	220464	14	4	2	4	3	1	1	1	4	1	2	4	3	4	3	3	1	1	3	1	1	3	3	2	4
2013_7	280366	12	4	2	4	3	4	1	1	2	1	2	4	3	4	3	3	1	1	3	1	4	3	3	4	4
2013_7	121263	9	4	1	2	3	1	2	1	2	2	2	4	2	2	1	4	1	3	1	1	2	2	3	4	4
2013_7	071257	10	4	2	4	3	4	4	1	4	2	2	2	2	0	1	3	1	4	0	1	0	3	0	3	1
2013_7	180666	10	4	2	4	2	3	3	1	2	2	2	4	3	4	3	2	1	4	1	1	1	3	4	4	1
2013_7	061164	9	4	2	4	4	1	3	1	4	2	3	4	2	4	3	4	1	3	4	4	2	3	4	3	4
2013_7	261166	11	4	1	4	3	1	2	1	4	2	0	1	2	4	3	4	2	1	4	4	2	1	3	4	3
2013_7	070363	10	4	1	4	3	1	2	1	4	2	3	1	2	4	3	4	1	1	1	1	2	1	4	3	3
2013_7	040866	8	4	0	4	3	4	4	2	4	2	2	2	2	0	1	3	1	4	0	1	0	0	3	4	4
2013_7	060165	12	4	2	4	3	1	4	1	2	2	2	4	3	4	3	4	1	1	4	4	2	1	4	2	4
2013_7	180458	9	4	2	4	3	3	4	1	2	2	1	2	2	3	3	3	1	3	3	1	3	1	1	4	3
2013_7	011063	10	4	2	4	3	1	4	1	4	2	1	4	3	3	4	2	1	1	3	4	2	1	2	3	1
2013_7	171061	14	4	2	4	3	1	4	1	4	2	1	4	3	3	4	3	1	4	4	4	2	2	2	3	2
2013_7	211168	7	3	4	4	4	3	1	1	4	2	3	4	3	3	1	4	1	3	3	1	2	3	1	4	3
2013_7	200458	12	4	2	4	4	1	1	1	2	2	2	4	3	2	3	4	1	3	3	1	2	2	3	3	3
2013_8	010969	14	4	2	4	3	3	2	4	4	1	2	1	3	4	3	3	1	3	3	1	2	3	2	2	3
2013_8	281262	9	4	2	2	3	4	2	1	4	2	3	1	3	3	1	4	1	3	4	1	1	3	1	3	4
2013_8	280268	11	4	2	2	2	3	3	1	2	0	2	1	3	1	3	4	1	1	4	1	3	2	4	4	3
2013_8	300161	11	4	2	4	3	3	3	1	2	1	2	1	4	1	3	2	1	4	3	4	2	2	3	4	3
2013_8	040964	11	3	2	2	3	3	4	4	4	2	3	4	3	3	3	3	4	4	4	4	4	3	3	4	3
2013_8	000001	10	2	2	4	0	4	2	1	4	1	3	3	3	1	4	4	1	4	2	1	2	1	2	4	4
2013_8	250463	14	3	2	4	3	1	2	1	4	2	3	2	4	1	3	3	1	4	4	3	2	1	2	4	3
2013_8	060862	10	4	2	2	3	1	2	1	2	2	3	1	3	4	3	4	1	1	4	1	2	1	3	3	4
2013_8	020660	12	3	2	4	3	3	2	1	2	2	2	4	3	2	4	4	1	1	4	1	2	2	3	4	3
2013_8	150562	14	4	2	4	3	3	1	1	2	4	2	4	3	1	3	3	1	1	2	1	4	1	4	2	4
2013_8	130958	14	4	2	4	3	1	3	4	4	2	3	1	3	3	3	3	2	4	4	1	2	1	3	4	3
2013_8	050166	9	4	2	2	3	3	1	1	2	2	3	1	3	1	3	4	1	1	3	1	2	1	4	4	3
2013_8	000002	8	4	2	2	3	3	1	2	2	2	2	1	3	1	3	4	1	3	3	1	3	1	1	3	4
2013_8	151066	11	4	2	4	3	3	3	1	2	2	2	2	3	1	3	4	1	1	4	1	2	1	4	3	4
2013_8	310165	10	3	2	4	3	1	2	4	4	2	2	2	3	3	1	4	1	1	4	1	3	3	3	4	4
2013_8	050770	16	4	2	4	3	3	2	1	2	3	2	2	3	1	3	3	1	4	2	1	2	3	1	2	3
2013_9	290458	8	3	2	1	3	3	3	2	4	2	2	1	1	3	1	3	2	4	1	1	2	1	3	4	1
2013_9	291254	10	4	1	2	3	1	3	1	2	2	2	2	4	4	3	4	2	3	3	1	2	2	4	3	3
2013_9	150767	15	4	2	3	3	1	2	1	4	2	2	4	4	1	3	4	2	4	1	1	3	2	4	4	4
2013_9	190657	7	2	2	3	3	4	3	1	4	2	2	1	4	2	3	3	1	3	1	4	2	4	1	3	4
2013_9	091066	11	4	3	3	3	3	2	1	4	2	3	1	4	4	1	3	2	4	2	1	4	2	3	4	1
2013_9	121057	7	4	1	2	3	1	3	1	2	2	3	2	2	4	1	2	2	3	4	4	2	4	3	4	3
2013_9	060565	12	3	2	4	3	2	2	1	2	2	1	4	4	3	3	4	4	4	4	1	3	2	3	2	4
2013_9	029957	11	4	2	3	3	3	3	1	4	2	2	4	4	1	3	4	1	4	2	4	2	2	3	4	4
2013_9	160859	8	4	2	4	3	3	3	1	4	1	1	1	4	4	1	4	1	2	4	1	2	3	3	4	4
2013_9	110962	6	2	2	1	3	1	3	2	1	2	2	1	1	1	1	4	1	1	3	1	2	3	3	4	4

2013_9	110264	9	4	3	3	3	4	3	1	4	1	2	1	4	1	2	3	1	1	3	4	3	1	3	2	3
2013_9	230665	6	4	2	2	3	3	1	2	4	2	2	1	4	2	1	4	1	1	4	4	2	1	3	3	1
2013_9	090958	8	4	3	3	3	1	3	2	2	2	4	1	4	2	1	3	2	1	2	1	4	2	3	4	2
2013_9	211062	6	2	3	1	3	3	2	2	2	2	2	2	1	1	2	3	1	1	3	1	3	1	3	3	4
2013_9	270764	12	4	2	4	3	1	3	4	2	2	2	1	4	4	3	3	2	1	4	1	2	3	1	2	4
2013_10	290160	8	4	1	3	3	1	2	1	4	2	2	1	4	3	1	4	1	4	2	3	1	3	3	3	2
2013_10	130165	6	4	2	3	3	3	2	2	4	2	3	1	4	3	1	4	1	1	3	1	2	1	3	4	4
2013_10	201167	13	4	2	3	3	1	1	1	4	2	2	4	4	1	3	4	1	4	4	3	2	1	3	4	3
2013_10	091064	11	4	2	2	3	3	2	1	4	2	2	4	4	3	3	4	1	1	0	1	2	2	1	4	4
2013_10	160362	10	4	2	3	3	1	2	4	4	2	2	4	4	2	4	4	1	1	2	2	4	1	3	4	3
2013_10	171166	12	4	2	3	3	3	2	1	4	2	3	4	4	2	3	4	3	1	3	1	2	1	2	2	3
2013_10	011153	9	4	2	3	3	1	1	1	4	2	2	1	4	3	3	4	2	1	3	3	2	4	3	4	4
2013_10	290856	4	3	2	1	2	3	1	2	2	2	2	4	1	4	1	4	1	3	3	2	2	2	4	4	2
2013_10	031061	8	4	1	3	3	1	3	1	2	2	1	1	4	3	3	2	1	2	3	3	4	2	1	2	1
2013_10	100461	9	4	1	4	4	3	2	1	4	3	2	1	4	4	1	4	4	1	3	1	4	1	1	4	4
2013_10	050459	9	4	2	3	3	3	1	4	4	2	3	2	4	4	3	3	1	4	4	1	2	3	1	4	1
2013_10	110562	9	4	2	3	3	3	2	1	4	0	1	4	4	4	0	4	1	3	3	1	2	3	4	3	3
2013_10	080264	9	4	2	3	3	3	1	3	4	1	3	2	4	3	3	3	1	1	4	1	2	2	1	3	1
2013_10	150659	16	4	2	3	3	1	2	1	4	1	2	1	4	1	3	4	2	4	2	1	4	2	4	3	3
2013_11	150862	10	4	2	1	3	1	2	1	4	2	3	1	4	3	1	4	1	4	4	3	2	2	4	3	4
2013_11	181160	10	4	2	3	3	3	3	1	4	1	1	1	3	4	1	4	3	1	4	1	3	3	2	2	4
2013_11	250358	8	4	2	3	3	4	1	3	4	4	2	2	4	3	1	3	1	4	2	3	2	2	3	1	4
2013_11	250657	8	4	3	3	3	3	1	1	4	2	3	1	4	3	4	4	1	1	4	1	2	3	2	4	3
2013_11	140865	7	4	3	3	1	3	2	3	1	2	4	2	3	1	1	3	1	3	1	1	4	4	1	3	2
2013_11	310865	6	3	3	2	2	2	4	1	4	2	4	2	3	4	1	4	4	3	1	1	4	2	1	4	1
2013_11	190862	3	4	3	3	2	2	4	3	2	2	4	0	3	3	0	4	4	3	1	1	1	3	3	1	4
2013_11	240263	4	4	0	3	1	0	4	1	2	4	0	0	4	4	0	0	4	2	1	1	4	0	0	0	2
2013_12	231160	9	3	2	4	3	1	2	1	2	2	2	1	0	0	0	4	2	1	4	0	0	0	0	0	0
2013_12	020967	9	3	2	3	3	3	3	1	2	2	2	4	4	1	4	1	2	3	1	4	4	1	4	2	1
2013_12	220864	10	4	2	3	3	2	2	1	4	1	2	4	4	2	4	1	3	2	4	4	4	1	3	1	2
2013_12	280770	9	4	2	3	3	3	2	1	4	2	1	4	3	2	1	3	1	1	3	4	2	3	3	4	4
2013_12	130463	8	3	2	1	3	1	2	1	4	2	3	4	4	2	2	4	3	0	3	3	2	4	4	3	3
2013_12	300369	8	2	2	3	3	3	4	3	4	2	2	2	4	4	0	4	1	4	4	4	3	2	2	4	1
2013_12	170465	9	4	2	3	3	3	2	4	2	2	2	1	4	3	1	4	2	1	4	1	2	2	3	3	1
2013_12	260163	8	4	2	3	3	3	2	4	4	2	2	2	0	0	1	4	1	0	3	1	2	2	0	4	4
2013_12	060965	10	4	2	4	4	3	4	4	4	2	1	4	4	1	3	4	1	3	4	3	2	2	1	2	4
2013_12	090766	6	2	1	3	3	3	2	2	2	2	2	2	0	0	0	3	2	0	2	4	0	0	0	0	3
2013_12	101960	7	4	2	3	3	3	3	2	2	2	2	4	4	4	3	4	1	2	4	3	2	3	3	3	4
2013_12	241163	10	3	1	3	3	4	3	4	4	2	2	4	4	1	1	3	2	4	3	1	3	3	4	3	3
2013_12	100764	4	2	2	3	1	3	3	1	4	1	2	2	4	2	2	4	1	1	1	4	3	1	4	4	4
2013_12	020262	9	2	2	2	3	4	1	1	4	2	2	3	0	0	0	3	3	1	1	3	4	2	2	3	2
2013_12	080962	11	2	3	4	3	4	2	1	4	2	2	4	4	1	2	4	1	4	4	1	3	3	3	3	4
2013_12	220768	6	4	3	3	3	3	3	2	4	2	2	2	4	0	1	4	4	3	4	4	2	2	4	4	4
2013_12	260560	7	4	2	2	3	4	2	2	2	2	3	1	1	0	2	3	1	1	2	0	4	2	0	3	2
2013_12	150169	6	4	2	0	3	3	3	1	2	0	4	1	4	0	2	3	1	0	3	0	2	2	0	0	0
2014_1	051165	10	4	2	2	3	1	3	1	2	3	3	1	3	4	1	4	1	1	2	1	3	2	4	2	2
2014_1	310161	13	4	2	4	3	3	4	1	4	2	2	4	2	2	3	4	4	3	3	1	4	1	2	4	3
2014_1	030869	11	4	2	1	3	1	3	1	2	2	2	4	2	2	1	1	1	3	4	1	3	2	2	4	4
2014_1	151063	10	4	2	4	2	1	3	2	2	4	3	4	2	2	3	3	2	1	4	1	2	1	3	4	4
2014_1	200864	8	4	2	4	2	2	1	1	4	2	1	2	2	1	1	4	1	1	3	1	2	3	2	4	4
2014_1	160669	9	4	1	4	2	3	3	1	4	2	1	4	3	3	1	4	1	4	4	1	2	3	3	3	4
2014_1	021264	10	4	2	4	3	2	2	1	2	2	1	1	2	4	1	3	1	4	4	1	2	3	3	3	4
2014_1	101263	9	4	2	4	3	2	2	4	2	3	1	1	3	2	1	4	3	4	3	3	2	2	4	4	1
2014_1	250860	7	4	1	2	3	3	4	1	2	2	3	1	3	3	4	2	1	4	4	1	2	3	3	4	1
2014_1	010363	12	4	2	4	3	4	2	1	4	2	2	1	4	4	3	4	1	4	4	1	2	3	3	3	2
2014_1	101062	6	2	2	4	2	3	3	1	4	4	4	2	2	3	2	4	1	4	3	4	3	3	3	2	4

2014_1	120467	10	4	2	1	3	3	1	1	4	2	1	2	3	1	1	4	1	1	2	1	2	2	4	2	2
2014_1	130767	6	3	3	4	3	3	1	2	2	2	2	2	2	2	1	3	3	4	3	3	2	2	4	4	4
2014_1	050567	4	4	2	4	3	4	4	4	2	2	1	2	2	3	1	4	1	1	2	4	0	1	3	4	2
2014_1	200962	11	4	2	2	3	4	2	2	4	2	2	4	2	4	3	3	1	1	2	1	2	3	4	4	3
2014_1	050265	9	3	2	1	3	4	2	1	2	1	2	1	2	4	3	4	3	1	4	1	2	1	2	4	4
2014_1	150263	13	4	3	4	3	3	2	1	4	2	2	4	3	4	1	4	2	3	4	1	2	2	3	4	4
2014_2	100663	14	4	1	2	3	1	2	1	4	2	2	4	3	4	3	4	1	1	0	1	4	2	4	2	4
2014_2	231067	9	4	2	2	3	1	1	1	4	2	2	1	3	2	1	4	1	3	3	1	1	1	3	3	4
2014_2	050660	8	2	2	2	3	3	2	1	4	2	2	1	3	3	1	4	1	1	2	3	2	1	4	3	3
2014_2	070759	8	4	2	2	3	3	3	2	4	2	2	1	2	1	1	4	1	1	3	1	3	3	4	2	4
2014_2	130165	10	4	2	4	2	1	1	1	2	2	2	4	2	4	3	3	1	1	0	1	3	3	4	4	2
2014_2	191159	9	4	1	2	4	4	1	4	4	2	1	1	3	1	3	4	2	1	4	3	2	2	2	4	1
2014_2	231056	8	2	2	4	3	3	2	1	4	4	3	1	3	3	1	4	3	3	2	1	3	1	1	4	1
2014_2	060256	7	2	2	4	4	3	1	4	2	4	3	1	1	3	1	4	1	2	2	1	4	3	2	2	3
2014_2	050867	11	4	2	2	3	4	2	1	4	2	1	4	1	3	4	4	1	4	3	1	2	2	3	4	3
2014_2	041167	8	4	2	2	3	1	3	2	4	2	2	2	4	4	1	0	1	3	1	1	2	1	3	4	3
2014_2	250865	14	2	2	2	3	1	3	1	2	4	2	4	2	4	3	3	1	4	4	1	2	2	3	2	3
2014_2	231161	10	4	2	2	3	1	3	1	2	2	2	2	3	1	1	4	1	4	3	1	2	3	1	4	4
2014_2	201261	10	4	3	2	3	2	2	1	4	2	2	4	2	2	1	3	4	1	4	1	0	0	0	0	0
2014_2	031262	17	4	3	4	3	1	2	4	4	2	2	4	3	1	3	4	1	4	4	1	4	3	3	2	3
2014_2	060964	7	4	3	2	3	1	1	2	4	2	3	2	1	3	1	4	1	4	2	1	3	1	2	4	2
2014_2	260868	7	3	2	2	3	3	3	1	2	1	2	1	1	1	1	2	1	4	3	1	1	0	3	4	1
2014_2	NA	13	4	2	4	3	4	3	1	4	2	2	4	2	1	3	4	1	4	0	1	0	2	0	4	0
2014_2	250363	5	4	2	2	3	3	3	2	2	3	3	2	1	3	4	1	1	4	1	4	2	3	3	4	2
2014_2	020768	12	4	2	2	3	4	2	1	4	2	2	1	3	2	1	4	3	1	3	1	0	2	2	4	3
2014_2	151064	10	4	2	4	3	1	2	1	1	2	2	4	4	4	1	4	1	4	3	4	3	1	4	4	4
2014_2	230158	13	4	3	4	3	4	2	1	4	2	2	4	3	4	3	4	4	4	3	1	3	2	3	4	1
2014_2	150959	9	4	2	2	3	1	2	1	2	2	2	4	2	4	1	4	4	3	0	4	4	4	3	4	4
2014_2	281066	15	4	2	4	3	1	2	1	4	2	2	4	2	4	3	4	1	4	4	4	4	2	0	4	1
2014_2	100760	12	3	3	4	3	3	2	1	2	2	2	4	2	2	3	3	1	4	3	1	2	2	0	2	4
2014_2	170261	9	3	2	4	2	1	2	1	4	2	3	2	2	4	1	4	1	4	4	4	2	1	1	2	2
2014_2	010250	12	3	2	0	3	3	2	1	4	2	2	4	3	4	3	4	1	4	4	1	3	3	3	4	2
2014_2	200263	9	4	2	2	3	4	1	1	4	2	2	4	4	3	2	4	4	1	1	3	1	2	3	4	3
2014_2	150766	11	4	2	4	3	3	2	1	2	1	1	1	1	1	3	4	1	4	4	1	2	3	3	3	2
2014_2	240360	14	3	2	2	3	2	3	1	4	3	2	1	3	3	3	4	1	4	4	1	4	1	1	2	3
2014_2	020752	11	4	1	2	3	3	2	1	4	2	2	1	2	4	3	4	4	1	4	1	3	2	3	2	4
2014_2	021063	10	4	2	4	3	3	1	2	4	2	2	4	2	3	3	2	1	3	4	1	3	1	4	3	1
2014_2	030859	12	3	2	4	3	4	2	1	2	4	3	2	3	4	1	4	2	4	4	1	2	3	2	2	4
2014_2	030869	12	3	2	4	3	1	3	1	4	2	2	4	2	1	3	3	1	1	3	1	1	1	4	4	4
2014_2	260265	16	4	2	3	3	1	2	1	4	3	2	4	3	4	3	4	1	4	4	1	4	3	1	1	1
2014_2	011258	12	1	2	4	3	1	3	1	2	2	2	1	3	2	3	0	4	4	4	4	0	2	2	0	1
2014_2	190370	10	4	2	2	3	1	2	1	4	2	3	1	2	1	1	4	1	1	1	4	2	2	2	4	4
2014_2	100468	6	4	3	4	3	1	1	2	2	2	1	2	2	4	1	3	1	1	1	4	3	4	2	0	0
2014_2	280264	9	4	2	4	2	3	2	1	2	2	3	1	2	3	4	3	2	4	3	1	2	0	1	0	0
2014_2	280864	10	1	2	4	1	4	2	1	4	2	2	4	3	4	2	4	4	4	2	3	4	1	4	4	1
2014_2	080864	12	3	2	4	3	3	3	1	4	2	2	1	2	4	3	3	1	4	4	1	4	1	4	3	4
2014_2	NA	9	4	1	2	3	4	3	4	4	2	1	1	3	0	3	3	1	1	3	1	4	3	3	3	3
2014_2	030463	12	4	2	2	3	4	3	1	4	2	2	4	2	1	1	4	1	1	4	4	2	2	0	2	3
2014_2	090662	13	4	1	4	3	1	2	1	4	2	2	1	4	0	3	4	2	4	2	3	4	1	0	1	3
2014_2	010364	9	4	2	2	3	3	3	1	2	1	2	1	2	4	1	4	1	4	4	1	4	3	3	4	4
2014_2	151256	8	4	2	2	3	3	2	1	2	2	2	4	3	4	4	4	4	1	0	0	0	0	0	0	0
2014_3	180471	13	4	2	4	3	1	3	1	2	4	1	4	3	3	3	3	1	1	4	1	2	2	3	4	4
2014_3	010462	10	3	1	4	4	3	0	4	2	3	3	4	3	1	3	3	2	1	2	1	2	2	1	4	2
2014_3	230163	8	4	1	4	3	4	1	3	2	2	1	1	2	3	3	3	2	4	2	3	3	3	3	2	4
2014_3	200467	9	4	1	1	3	3	2	1	2	2	2	2	2	1	1	2	3	4	4	1	3	3	3	4	2
2014_3	201163	9	4	2	4	3	1	3	1	2	1	4	3	4	1	1	4	1	3	4	3	1	2	3	4	4

2014_3	080964	14	4	2	2	3	3	2	1	4	1	2	1	3	1	3	1	2	2	4	3	2	2	3	1	3
2014_3	010867	7	2	2	2	3	3	2	2	4	2	4	2	2	1	2	4	1	1	4	1	2	1	3	4	1
2014_3	261056	13	4	2	4	3	3	2	1	4	1	2	1	3	1	3	4	4	1	3	1	2	3	3	4	3
2014_3	010256	15	4	2	4	3	4	1	1	4	2	2	4	2	2	3	4	1	1	4	1	4	4	2	2	3
2014_3	171263	9	4	2	4	3	2	2	1	1	2	2	2	4	1	2	4	3	3	3	1	3	3	3	4	4
2014_3	181267	9	4	2	2	2	4	2	1	2	3	3	1	2	1	3	4	1	1	1	4	3	2	2	3	4
2014_3	130262	13	4	2	4	3	1	3	1	2	4	4	1	3	1	1	3	1	1	4	3	3	2	2	4	3
2014_3	090266	9	4	3	2	3	1	3	4	1	2	2	2	4	3	1	3	2	0	2	2	4	2	3	1	3
2014_3	011062	11	4	2	4	3	4	2	2	4	2	1	2	2	4	1	3	1	4	3	1	2	2	3	4	3
2014_3	300470	8	4	4	4	2	1	3	1	3	4	2	2	1	3	4	2	3	4	2	4	4	4	3	2	4
2014_3	270464	10	3	2	4	3	3	2	1	2	4	4	3	4	3	3	2	1	4	3	3	4	2	2	4	4
2014_3	141258	5	3	1	2	3	3	2	1	2	2	4	1	2	4	3	4	1	1	2	4	3	2	4	1	2
2014_3	190273	8	4	2	2	1	3	2	4	2	3	4	4	4	3	1	3	3	1	3	3	1	2	3	4	3
2014_3	030259	10	4	2	4	2	2	2	1	4	4	1	1	3	2	4	3	1	3	4	2	3	1	3	2	4
2014_3	181161	10	4	2	4	2	2	2	1	2	4	2	3	2	3	2	3	1	4	3	1	1	1	3	1	3
2014_4	261171	12	4	2	4	3	1	2	1	2	2	2	4	3	2	1	4	1	1	3	1	2	3	2	3	4
2014_4	290964	7	4	2	4	1	3	3	1	2	1	3	1	1	1	1	4	3	3	3	1	3	3	2	3	1
2014_4	040266	11	4	3	4	2	3	3	1	4	2	1	4	3	2	1	3	1	4	4	1	3	3	3	1	3
2014_4	160358	9	4	2	4	3	3	1	1	4	2	1	1	3	3	1	3	1	1	3	4	2	1	0	2	4
2014_4	190463	12	4	2	4	3	1	2	1	4	1	3	2	3	3	2	3	1	3	3	4	2	2	4	2	2
2014_4	170659	12	4	2	4	3	3	3	1	4	2	2	0	3	4	1	4	4	1	4	1	4	4	4	2	4
2014_4	300365	10	3	2	4	3	1	1	1	4	4	2	1	3	4	1	3	1	1	3	4	2	2	4	4	1
2014_4	091162	13	4	2	4	3	4	1	1	4	2	2	4	3	1	3	4	1	1	4	4	3	2	4	4	1
2014_4	220766	12	4	2	4	3	3	3	1	4	2	2	1	3	3	2	4	4	3	4	1	4	4	4	2	4
2014_4	051066	9	4	2	4	1	4	3	1	2	2	2	2	3	2	1	2	1	3	4	4	4	1	3	2	4
2014_4	190159	13	4	2	2	3	1	3	1	1	2	2	1	2	2	3	3	1	4	4	1	4	4	4	2	4
2014_4	210260	8	3	3	4	3	3	3	4	1	2	3	4	3	4	3	3	1	4	3	4	3	4	4	2	4
2014_4	NA	5	3	1	3	3	1	3	2	4	2	3	1	4	2	3	1	1	1	1	1	1	3	1	4	4
2014_4	260854	8	4	2	2	3	4	1	2	4	2	1	2	1	4	3	3	1	2	2	1	4	3	1	4	2
2014_4	260664	7	4	2	2	3	4	2	2	2	1	2	4	3	2	4	4	3	2	3	2	1	1	4	1	2
2014_5	120773	14	4	2	2	3	3	2	1	4	2	3	4	3	4	3	3	4	4	4	1	2	3	1	3	3
2014_5	141167	13	4	2	4	3	3	2	4	4	2	2	4	3	3	1	3	1	4	4	3	3	4	3	4	3
2014_5	151264	11	4	1	4	2	2	3	1	4	1	2	4	2	1	3	4	1	1	4	1	2	2	4	3	4
2014_5	111063	8	4	2	2	3	2	3	4	4	2	3	2	2	3	1	3	1	1	3	1	3	2	2	4	4
2014_5	060262	13	4	2	4	3	2	3	1	4	2	2	4	4	4	3	4	1	4	4	4	4	3	1	4	3
2014_5	140965	10	4	3	1	3	3	4	1	2	2	2	3	1	4	3	3	1	4	4	1	4	1	3	4	2
2014_5	290862	13	4	2	4	3	1	2	2	2	2	2	4	3	1	2	4	1	4	3	1	4	1	0	0	0
2014_5	260867	15	4	2	4	3	2	3	1	4	2	2	4	3	4	3	3	1	1	4	1	4	2	4	4	4
2014_5	040363	10	4	2	2	3	1	1	1	2	2	3	4	3	3	3	4	1	4	3	1	1	4	4	4	2
2014_5	140264	11	4	2	4	3	3	1	1	4	1	3	1	1	3	3	2	1	4	3	4	4	1	2	3	3
2014_6	221267	12	4	3	4	3	1	1	1	4	2	2	4	2	4	3	4	1	3	4	1	4	4	3	0	4
2014_6	311064	8	2	2	2	3	1	4	1	4	2	4	0	2	4	1	4	1	3	4	1	3	2	0	3	4
2014_6	281168	12	3	2	4	1	1	1	1	4	2	2	4	3	4	3	3	3	3	3	1	3	1	3	4	3
2014_6	250263	14	3	2	4	3	1	2	1	2	2	2	4	3	4	3	4	2	4	4	1	2	3	3	3	2
2014_6	221155	12	3	2	4	3	1	2	1	4	1	3	1	3	4	3	3	3	4	3	1	3	1	3	4	4
2014_6	151059	15	4	2	4	3	1	3	1	2	1	2	4	3	4	3	4	1	4	4	1	4	2	4	4	4
2014_6	181264	8	3	1	4	3	1	1	1	4	2	3	1	3	4	1	4	1	1	2	1	3	2	3	3	2
2014_6	020257	6	3	4	2	3	3	3	1	4	2	3	2	1	1	1	4	1	4	2	1	3	1	3	4	2
2014_6	070369	12	3	2	4	3	1	1	1	4	2	2	2	3	2	3	4	1	4	3	1	1	3	2	4	4
2014_6	060864	10	4	2	4	2	1	3	1	2	2	2	4	2	0	3	4	1	1	0	1	4	1	0	3	1
2014_6	030959	15	3	2	4	3	1	2	1	4	2	2	4	3	0	3	4	1	4	4	3	4	2	4	4	4
2014_6	170765	12	3	2	4	3	1	2	1	4	2	2	4	3	1	2	4	1	4	1	3	0	0	0	0	0
2014_7	070856	10	4	2	4	3	3	4	1	4	2	2	2	2	3	1	4	3	4	4	4	3	3	1	4	3
2014_7	230569	9	1	2	4	3	3	2	1	4	2	2	1	2	3	1	1	4	4	4	4	2	3	1	3	1
2014_7	150263	7	4	3	4	4	3	1	1	2	2	2	1	4	3	1	3	1	1	2	1	2	1	4	4	3
2014_7	030475	5	3	3	3	3	3	1	1	4	2	2	2	2	1	1	4	3	1	3	4	2	1	4	3	4

2014_7	200666	6	3	3	4	3	3	1	1	4	2	2	1	2	3	1	4	1	1	2	4	2	2	3	1	4
2014_7	210855	10	4	3	4	3	3	2	1	4	2	2	2	2	3	3	2	1	4	4	3	3	3	4	4	4
2014_7	120464	8	2	2	3	3	4	2	2	4	1	3	2	1	4	2	4	1	4	4	1	2	3	1	2	2
2014_7	070664	6	4	4	4	3	2	4	1	4	2	3	2	2	4	1	4	1	1	2	4	2	1	4	2	4
2014_7	070367	9	4	3	4	3	1	2	3	4	2	1	2	3	4	2	2	1	3	4	2	2	3	3	2	4
2014_7	020966	6	4	4	4	3	4	4	1	4	2	2	2	2	4	1	4	1	1	1	4	2	3	1	3	2
2014_7	200770	6	4	2	4	3	4	2	1	2	2	3	2	2	3	2	4	1	1	3	4	2	3	1	3	4
2014_7	260866	14	4	2	4	3	3	2	1	4	2	2	4	2	2	1	3	4	3	4	4	1	2	2	4	3
2014_7	250470	13	4	2	4	3	3	1	1	4	2	2	4	1	1	1	3	1	1	4	4	2	2	2	4	2
2014_7	080860	10	4	2	4	3	4	2	1	4	4	2	2	2	3	4	2	3	1	3	2	1	2	4	2	2
2014_7	080662	10	4	2	4	3	1	2	1	2	2	2	1	2	3	4	3	2	1	1	4	3	1	3	3	4
2014_7	200364	12	4	2	4	3	1	2	1	4	2	2	1	4	3	4	3	3	1	1	4	3	1	2	2	4
2014_7	280464	8	2	2	4	3	1	4	3	4	1	3	4	1	4	1	4	1	1	1	4	2	2	1	2	4
2014_7	NA	11	4	3	2	3	3	3	1	4	2	2	4	2	3	3	4	1	4	4	1	2	2	3	1	4
2014_7	110571	8	4	2	4	2	3	4	1	2	2	3	1	3	4	4	4	1	3	4	1	3	2	1	3	1
2014_7	NA	11	4	2	4	3	3	3	1	2	2	2	4	3	4	1	4	2	3	2	1	2	3	3	2	4
2014_7	100664	9	4	2	4	3	3	3	1	2	2	2	4	3	4	1	4	2	3	3	2	2	1	1	3	2
2014_7	060472	11	4	2	2	3	3	2	1	4	2	3	4	1	1	3	4	1	3	3	4	4	2	1	3	1
2014_7	220177	9	4	2	4	3	3	3	1	2	2	2	4	3	4	1	4	2	3	2	2	2	1	3	3	4
2014_7	090161	9	4	2	4	3	3	3	1	2	2	2	4	3	4	1	4	2	3	3	2	2	1	3	3	4
2014_7	241163	10	4	2	1	3	3	2	1	4	2	3	4	4	4	1	3	3	1	4	4	2	2	4	4	2
2014_7	140369	7	4	2	2	3	4	2	4	2	2	2	1	2	4	1	4	1	1	3	1	1	2	3	3	4
2014_7	160661	9	4	2	2	3	4	2	1	2	2	2	4	2	4	1	4	1	1	3	1	1	2	3	3	4
2014_7	141062	7	4	2	2	3	4	2	4	2	2	2	1	2	4	1	4	1	1	3	1	1	2	3	3	4
2014_7	030265	5	2	2	2	2	1	4	2	4	1	2	3	2	4	1	4	1	1	3	4	1	4	3	2	4
2014_7	010364	5	4	2	2	3	1	3	2	2	1	3	2	2	4	1	4	4	2	3	1	2	3	3	4	1
2014_7	160364	9	4	2	2	3	2	3	1	4	2	3	1	3	4	1	4	4	2	4	1	2	3	3	2	4
2014_7	150476	6	4	2	4	3	4	4	2	4	4	4	2	3	3	1	4	1	1	3	4	2	3	3	3	4
2014_7	070567	10	4	2	4	3	3	3	1	4	1	3	1	3	3	1	4	1	4	4	1	2	3	4	3	1
2014_7	311055	11	4	2	4	3	2	1	1	4	4	2	1	3	1	1	4	1	3	2	1	2	3	3	2	1
2014_7	300177	8	4	2	4	3	3	2	1	4	2	1	1	2	1	1	4	3	3	3	3	2	1	3	4	4
2014_7	181063	6	3	4	4	2	4	3	4	4	1	2	3	3	4	2	4	1	4	3	4	2	2	4	4	2
2014_7	150863	9	2	2	4	4	4	1	1	4	2	3	1	2	4	1	4	1	4	4	1	2	2	2	4	1
2014_7	121270	10	2	2	4	3	3	2	1	4	1	2	1	4	3	1	4	1	1	4	1	1	2	3	4	4
2014_7	110366	8	3	2	4	3	3	2	3	4	1	2	2	4	4	1	4	1	4	3	3	2	2	3	4	1
2014_7	130562	6	3	1	4	3	3	1	2	2	4	1	2	3	4	1	4	2	4	4	4	2	3	3	4	2
2014_7	100565	10	3	2	4	3	3	2	1	4	2	1	1	2	3	4	4	2	4	1	1	2	2	3	3	2
2014_7	280762	7	1	3	4	4	3	2	3	4	2	1	1	3	3	1	4	1	4	4	4	2	1	2	4	2
2014_7	240265	7	2	1	4	3	4	1	2	4	2	1	1	3	3	1	4	1	4	3	1	2	2	3	4	4
2014_7	101163	7	4	1	4	4	4	1	1	2	2	1	4	2	2	3	4	1	3	2	3	1	2	1	3	3
2014_7	300363	8	4	1	4	3	3	3	1	2	2	1	4	2	3	1	3	1	1	2	4	1	2	1	2	4
2014_7	180662	8	4	1	4	3	3	2	4	2	2	1	1	3	4	1	4	2	1	2	4	3	2	1	2	4
2014_7	161164	10	4	4	4	3	4	3	4	4	2	2	1	4	2	3	4	1	4	4	4	4	1	3	2	4
2014_7	010556	9	4	3	4	0	4	2	2	4	2	2	4	2	2	1	4	1	4	0	1	2	3	0	2	2
2014_7	090868	11	1	2	4	3	3	1	1	4	1	2	1	2	1	1	3	2	4	4	4	2	1	4	3	4
2014_7	171165	11	1	2	4	3	3	3	1	4	1	2	1	2	4	1	2	1	4	4	1	2	2	2	3	1
2014_7	300362	9	4	2	4	4	4	3	1	4	2	1	1	3	1	1	4	1	3	4	1	2	3	4	4	4
2014_7	050264	3	1	1	3	1	2	3	2	4	1	4	2	2	4	1	4	1	2	3	4	2	4	4	2	3
2014_7	230868	11	4	2	4	3	3	2	1	4	2	1	1	2	1	1	4	2	1	4	1	3	3	3	4	1
2014_7	120671	7	4	2	4	4	3	3	1	4	2	3	1	1	3	1	4	1	4	2	1	3	3	4	1	1
2014_7	250464	7	4	3	4	3	3	1	2	4	2	2	1	3	3	1	4	3	3	3	1	2	3	4	4	4
2014_7	030866	9	4	2	4	3	4	2	2	2	2	2	3	2	3	3	3	4	1	2	3	1	3	4	4	3
2014_7	280553	12	4	2	4	3	3	1	1	4	2	2	2	2	1	4	4	3	4	4	1	2	3	3	2	4
2014_7	130963	12	4	2	4	3	3	1	4	2	2	2	2	2	1	1	4	1	3	4	1	4	2	1	2	3
2014_7	231066	12	4	2	4	3	3	1	4	2	2	2	2	2	1	1	4	1	3	4	1	4	2	1	2	3
2014_7	100768	10	4	2	4	2	1	2	2	1	2	2	2	2	4	1	3	4	3	4	3	4	4	1	3	3

2014_7	181066	8	4	2	4	2	4	2	2	4	2	2	2	2	3	3	4	1	3	4	4	1	1	3	3	4
2014_7	170462	12	4	2	4	3	1	2	1	4	2	2	4	2	4	3	4	1	2	2	1	2	3	3	4	1
2014_7	060867	14	4	2	4	3	1	2	1	4	2	2	4	4	4	3	3	1	2	1	4	2	2	1	3	3
2014_7	100866	15	4	3	4	3	1	3	1	2	3	2	2	3	1	3	4	2	3	4	1	4	2	3	3	4
2014_7	090970	11	4	2	4	3	4	1	1	4	2	2	2	2	3	1	4	1	4	4	1	2	2	3	3	4
2014_7	150358	8	4	3	4	3	3	3	2	4	2	2	4	2	3	1	4	4	1	4	1	2	1	3	4	2
2014_7	030667	14	4	2	4	3	4	2	1	4	2	2	1	2	1	1	3	4	1	4	1	4	2	3	3	2
2014_7	140274	14	4	2	4	3	4	2	1	4	2	2	1	2	1	1	3	4	1	4	1	4	2	3	3	4
2014_7	310764	11	3	2	4	3	4	2	1	2	2	1	2	2	1	1	3	2	1	4	1	1	3	2	4	2
2014_8	270364	13	4	2	2	3	1	2	1	4	2	3	4	3	2	1	4	1	1	4	4	2	2	2	4	3
2014_8	290767	7	3	3	4	4	3	2	2	4	2	2	2	1	3	1	3	2	3	4	4	3	3	4	4	2
2014_8	241165	7	4	3	2	3	1	3	4	4	2	2	1	1	3	1	4	4	1	3	1	0	2	4	1	4
2014_8	230267	11	3	1	4	3	3	2	1	4	2	2	1	1	3	3	4	1	4	3	1	4	2	3	4	4
2014_8	010663	9	4	2	4	3	2	4	0	4	2	2	4	2	3	3	4	1	4	3	4	2	3	0	0	0
2014_8	130471	16	4	2	4	3	1	2	1	4	2	2	4	3	2	4	4	1	4	4	1	4	3	4	4	3
2014_8	151064	9	4	2	2	3	3	3	1	2	2	3	2	2	4	3	3	1	0	4	4	4	4	0	3	3
2014_8	170465	5	3	4	4	3	3	1	4	2	2	1	1	2	3	3	4	1	4	3	4	2	3	3	2	1
2014_8	090859	14	3	2	4	3	1	1	1	2	3	2	1	3	1	3	3	4	4	4	4	3	1	4	4	3
2014_8	281263	13	4	2	4	3	1	3	4	4	2	2	4	3	2	3	4	1	3	4	4	2	2	1	2	4
2014_8	261254	13	4	2	3	3	1	3	1	4	2	2	4	3	2	3	4	1	1	4	1	2	1	3	2	4
2014_8	101058	12	4	2	4	3	1	1	1	4	2	3	4	3	3	3	4	1	4	2	1	2	1	3	4	2
2014_8	230964	12	4	2	4	3	3	1	2	4	2	3	1	3	1	1	3	1	4	1	4	4	1	3	2	3
2014_9	160259	8	4	4	4	2	3	3	1	2	2	3	4	1	2	1	4	2	4	2	3	3	1	2	3	3
2014_9	120570	9	4	2	4	2	4	1	1	4	2	2	1	3	2	3	4	4	3	2	3	3	1	1	4	3
2014_9	300164	9	4	1	4	3	3	2	1	4	1	3	1	4	3	1	4	1	4	4	4	1	3	1	2	4
2014_9	151163	12	4	2	2	3	3	2	4	4	2	2	4	1	3	1	4	4	1	4	4	4	2	2	2	4
2014_9	171060	10	4	4	4	3	1	0	1	4	2	3	4	1	3	3	3	2	3	3	4	3	3	3	3	4
2014_9	240765	11	4	2	4	3	4	2	1	2	1	3	1	2	2	1	4	4	1	4	1	4	2	1	4	3
2014_9	220364	8	4	1	4	3	3	3	1	4	2	2	4	4	2	2	4	1	4	3	4	3	1	3	3	2
2014_9	081066	12	4	2	4	3	3	1	1	4	4	2	4	2	1	3	4	1	1	3	2	4	1	3	4	3
2014_9	060363	12	4	2	4	3	3	1	1	4	2	2	3	1	1	2	4	4	1	4	4	2	2	2	2	4
2014_9	280769	11	4	2	4	3	3	4	1	4	2	2	2	1	1	2	4	3	4	4	4	2	4	2	4	4
2014_9	NA	10	4	2	3	3	4	3	2	4	1	2	2	1	4	3	4	2	4	4	4	3	1	2	4	1
2014_9	030163	13	4	2	4	3	3	3	1	4	1	2	4	1	3	3	4	2	4	1	1	2	2	3	3	4
2014_9	090762	9	3	2	4	3	1	3	1	2	2	3	1	3	3	2	4	2	4	4	4	3	1	1	4	4
2014_9	160666	9	4	2	4	3	2	1	4	2	2	2	4	3	1	1	4	1	4	2	4	2	1	4	4	4
2014_9	090569	10	3	2	1	3	3	4	1	4	2	3	4	3	1	1	3	1	0	3	3	2	3	2	4	3
2014_9	230861	10	4	2	4	2	3	2	2	4	2	3	1	3	4	1	4	2	4	4	4	2	1	3	4	3
2014_9	110160	10	3	2	4	2	1	4	3	4	2	2	1	3	3	1	4	2	2	4	4	1	1	2	4	3
2014_9	280868	12	4	2	4	3	3	1	1	4	1	3	4	3	3	1	3	2	1	4	1	2	3	1	4	4
2014_9	220865	12	4	2	3	3	2	4	1	4	2	2	4	3	3	2	3	2	1	2	4	4	3	3	4	3
2014_9	140865	13	4	1	2	3	4	3	1	4	2	2	4	4	3	2	3	2	4	1	1	4	2	3	3	3
2014_9	070658	12	4	1	2	3	1	4	1	2	2	2	4	4	1	2	3	2	4	4	4	4	3	4	3	4
2014_9	200360	7	4	2	3	2	3	3	1	4	1	2	1	2	4	1	4	1	4	2	4	2	2	3	3	4
2014_9	110664	15	4	2	4	3	3	4	1	2	1	2	4	3	2	3	3	1	4	4	1	4	3	3	3	3
2014_9	260263	9	3	2	4	2	3	2	1	2	2	2	2	3	4	1	4	1	4	3	1	2	1	3	4	3
2014_9	260766	14	4	2	4	3	4	1	1	4	3	2	1	3	1	1	3	2	4	2	4	3	2	3	3	1
2014_9	151266	12	4	2	4	3	1	2	1	2	1	2	1	1	3	1	3	1	1	3	1	1	2	1	3	3
2014_9	271054	9	4	3	4	3	3	2	4	4	2	2	2	1	4	1	4	3	4	4	4	1	1	2	4	4
2014_9	260465	14	3	2	4	3	1	2	1	4	1	2	2	4	1	2	3	2	4	2	1	4	1	3	4	4
2014_9	181070	13	3	2	3	3	3	1	1	4	2	2	1	3	1	3	3	1	4	4	1	3	2	3	4	4
2014_9	221160	12	4	2	4	3	3	1	1	4	2	2	4	1	4	2	3	1	1	4	4	1	2	3	3	3
2014_9	010862	9	3	2	4	3	3	1	1	4	4	2	2	0	4	3	4	1	4	2	1	2	3	1	4	4
2014_9	250168	9	3	2	2	3	1	3	1	4	2	2	1	1	4	4	3	3	4	0	1	2	1	4	3	4
2014_9	060762	13	4	2	4	3	1	3	1	4	2	2	1	3	1	3	4	3	4	1	1	3	1	3	4	4
2014_9	290368	8	4	2	2	3	3	3	2	4	2	3	1	1	1	1	4	3	4	1	1	3	2	4	3	4

2014_10	150163	17	4	2	4	3	1	3	1	4	3	3	4	4	1	3	4	1	4	4	1	4	2	2	4	4
2014_10	170659	12	4	2	4	3	1	3	1	4	4	1	1	3	4	3	3	1	1	3	1	3	2	3	3	4
2014_10	220174	15	4	2	4	3	1	2	1	4	3	2	0	3	3	3	4	1	3	4	1	2	3	4	4	3
2014_10	130965	10	4	3	4	3	4	2	1	2	2	4	4	2	4	1	4	4	4	4	1	4	3	4	3	4
2014_10	290862	14	4	2	4	4	1	1	1	4	2	2	1	3	2	3	3	3	4	4	1	3	2	3	4	4
2014_10	171063	20	4	3	4	3	1	2	1	4	3	2	4	3	2	3	3	3	4	4	1	4	2	2	4	3
2014_10	140668	12	4	2	1	3	3	2	1	4	2	2	4	3	1	4	4	1	4	2	4	2	2	4	4	4
2014_10	060663	9	3	2	2	3	1	3	1	2	2	2	4	2	3	4	3	1	4	3	4	4	1	4	3	4
2014_10	140564	13	4	2	4	3	1	2	1	4	2	2	4	3	3	3	4	1	4	1	4	2	3	0	4	4
2014_10	110363	7	4	4	2	1	1	1	1	4	4	2	4	2	4	3	4	1	1	3	3	1	3	1	4	4
2014_10	181065	12	4	1	2	3	3	2	3	4	2	2	4	3	1	4	4	1	1	4	1	4	2	3	4	4
2014_10	071264	13	3	2	2	3	3	2	1	4	2	2	3	3	2	3	3	1	3	4	1	4	1	1	3	3
2014_10	231163	9	4	2	2	3	3	3	4	2	2	2	1	2	1	1	3	4	4	4	4	4	1	3	4	4
2014_10	230565	11	4	3	2	3	4	2	1	1	2	3	4	3	1	3	4	1	1	1	1	2	2	3	1	3
2014_10	230566	15	4	2	2	3	1	1	1	4	4	2	4	2	2	3	3	2	4	4	1	3	3	2	4	1
2014_10	111064	13	4	2	4	3	4	4	1	4	2	2	1	3	1	1	4	1	4	4	1	2	4	2	3	4
2014_11	010460	13	2	2	4	3	1	2	1	2	1	2	2	3	4	3	4	2	4	2	4	1	2	0	0	3
2014_11	140859	14	1	2	4	3	1	3	1	4	1	2	0	3	4	3	3	1	4	2	4	4	2	1	3	3
2014_11	080368	7	2	2	2	3	3	4	1	4	2	4	2	4	0	1	4	1	1	4	1	2	4	0	2	1
2014_11	221266	9	3	2	4	3	1	3	2	4	2	3	1	3	2	3	4	3	2	4	1	3	4	3	3	4
2014_11	130564	11	3	2	2	3	1	1	1	2	1	1	4	3	0	3	4	1	1	0	1	4	1	2	4	3
2014_11	090867	12	4	2	2	3	1	2	1	4	1	2	2	3	3	1	3	1	1	4	1	2	1	3	4	4
2014_11	100657	16	4	2	4	3	1	3	1	4	2	3	1	3	1	3	3	2	4	4	4	4	3	3	0	3
2014_11	110761	9	4	2	4	3	1	3	1	4	2	3	1	3	2	1	4	1	4	2	3	3	1	3	4	4
2014_11	310765	9	4	2	4	0	1	3	1	4	2	3	1	3	3	1	4	1	4	3	4	2	3	4	2	4
2014_11	231263	11	3	2	2	3	3	2	2	4	2	2	4	2	3	3	2	1	4	3	1	2	2	4	2	4
2014_11	021066	10	4	2	4	3	1	4	1	4	2	3	1	3	3	3	4	1	4	3	4	3	3	4	0	0
2014_11	060968	11	3	2	2	3	1	1	1	4	2	2	2	2	4	3	4	1	4	2	1	4	3	3	2	4
2014_11	280361	10	3	2	2	3	3	1	1	4	2	3	2	1	2	3	3	1	4	3	4	4	2	3	4	3
			Q1	Q2	Q3	Q4	Q5	Q6	Q7	Q8	Q9	Q10	Q11	Q12	Q13	Q14	Q15	Q16	Q17	Q18	Q19	Q20	Q21	Q22	Q23	Q24
		1	2.9	9.7	3.4	3.4	36.6	21.3	72.4	2.3	15.4	10.5	33.7	9.5	24.6	39.6	2.3	64.0	35.6	8.4	57.3	8.6	28.6	15.4	5.5	14.1
		2	8.2	76.8	24.2	6.9	5.9	39.4	12.0	30.9	70.7	60.0	21.5	31.4	17.7	7.6	5.5	15.4	4.2	15.2	2.5	38.7	35.6	14.1	19.8	12.2
		3	17.3	9.7	9.9	85.1	40.0	29.5	3.6	0.6	6.1	25.1	2.5	41.3	23.4	43.4	30.1	10.7	15.8	25.7	11.6	23.8	27.2	40.6	24.0	24.6
		4	71.4	3.4	62.1	3.8	17.3	8.8	11.2	65.9	6.7	3.8	40.8	16.0	30.1	7.6	61.1	9.7	42.3	47.6	27.4	24.0	6.5	22.1	46.5	45.1
		0	0.2	0.4	0.4	0.8	0.2	1.1	0.8	0.2	1.1	0.6	1.5	1.9	4.2	1.9	1.1	0.2	2.1	3.2	1.3	4.8	2.1	7.8	4.2	4.0

Table 2: Performance of students (cohort 2013) to select the best answer on all BCI questions

Group	Student ID	Q1	Q2	Q3	Q4	Q5	Q6	Q7	Q8	Q9	Q10	Q11	Q12	Q13	Q14	Q15	Q16	Q17	Q18	Q19	Q20	Q21	Q22	Q23	Q24	Score
2013_1	121245	1	1	1	1	1	0	1	0	0	0	1	1	0	1	0	1	0	0	1	0	0	0	1	0	50.0
2013_1	260857	1	1	1	1	1	0	1	0	0	1	0	0	0	1	0	0	0	1	1	0	1	0	0	0	45.8
2013_1	070565	1	0	1	1	0	0	0	0	0	0	0	0	0	0	0	0	0	1	0	0	0	0	0	0	16.7
2013_1	260960	0	1	1	1	0	1	1	0	0	1	0	0	1	0	0	0	1	0	1	0	0	0	0	0	37.5
2013_1	060169	0	1	1	1	0	1	0	1	0	1	0	0	0	0	0	0	0	0	0	0	0	0	0	0	25.0
2013_1	100264	0	1	1	1	0	1	1	1	0	0	0	0	0	1	0	0	0	1	1	0	0	0	1	0	41.7
2013_1	270362	0	1	1	1	0	0	0	0	0	0	1	0	0	0	0	0	0	0	1	0	1	0	0	0	25.0
2013_1	170962	1	1	1	1	0	0	1	1	0	1	0	1	0	0	1	0	0	1	1	0	1	0	0	0	50.0
2013_1	100262	1	1	1	1	0	1	1	1	0	1	1	0	0	0	0	0	0	0	0	0	0	0	0	0	37.5
2013_1	061156	1	1	1	1	0	1	1	1	0	1	0	0	1	0	0	0	1	1	1	1	0	0	0	0	54.2
2013_1	150858	0	1	0	1	1	0	0	0	0	0	0	0	0	0	0	0	1	1	1	0	0	0	0	0	25.0
2013_1	270461	0	1	1	1	0	0	1	1	0	1	1	1	0	1	1	0	1	1	1	0	0	0	0	1	58.3
2013_1	071254	1	1	1	1	0	0	1	1	0	0	1	0	0	0	0	0	0	1	0	1	0	0	0	0	37.5
2013_1	240260	1	1	1	1	0	0	0	1	0	1	1	1	1	1	0	0	0	1	0	0	0	0	0	0	45.8
2013_1	261167	1	1	1	1	1	0	0	1	0	0	0	0	1	0	0	1	0	1	0	0	0	0	0	0	37.5
2013_1	230254	0	0	0	1	1	0	1	1	0	0	0	0	0	1	0	0	0	0	0	0	0	0	0	0	20.8
2013_1	050162	1	1	1	1	0	0	1	1	0	0	0	0	0	0	1	0	0	1	1	1	1	0	0	0	45.8
2013_1	260760	1	0	0	1	0	0	1	0	0	1	0	0	0	0	0	1	0	0	1	1	0	0	0	0	29.2
2013_1	030361	1	1	1	1	0	0	1	1	1	0	0	1	0	0	0	0	0	1	1	0	0	0	0	0	41.7
2013_1	231163	1	1	1	1	1	0	1	1	0	1	1	1	0	1	0	0	0	1	1	0	0	0	0	0	54.2
2013_1	040766	1	0	1	1	0	0	1	1	0	0	0	0	0	1	0	0	0	1	0	0	1	0	0	0	33.3
2013_1	120263	1	0	0	1	1	0	1	1	0	0	1	1	1	0	1	0	0	0	1	0	0	0	1	0	45.8
2013_1	250964	1	1	1	1	0	1	1	1	0	0	0	0	0	0	0	0	1	1	0	0	1	0	0	0	41.7
2013_1	191159	0	1	1	1	1	1	1	1	0	1	0	1	0	1	0	0	1	0	0	0	0	0	0	0	45.8
2013_1	150766	0	1	1	0	0	0	1	1	0	1	1	0	0	0	0	1	1	1	1	0	0	0	0	0	41.7
2013_1	140468	0	1	0	1	1	0	1	0	0	0	0	0	0	0	1	1	0	0	0	0	0	0	0	0	25.0
2013_1	160968	0	1	1	1	0	0	1	1	0	0	0	0	0	0	0	0	0	1	0	1	0	0	1	0	33.3
2013_1	060761	0	1	1	1	0	1	1	1	0	0	0	0	0	1	0	0	0	0	0	1	0	0	1	0	37.5
2013_1	150566	1	1	1	1	1	1	1	1	0	1	1	1	0	1	0	0	0	1	1	1	1	0	1	1	75.0
2013_1	080552	1	1	1	1	1	1	1	1	0	1	0	0	0	0	1	1	0	1	1	0	1	0	1	1	66.7
2013_1	010451	0	1	0	1	0	1	0	0	0	0	0	0	0	0	0	0	0	0	1	1	1	1	0	0	29.2
2013_1	230755	1	1	1	1	1	1	1	1	0	1	0	0	0	0	0	0	0	1	1	0	1	0	1	0	54.2
2013_1	120471	1	1	1	1	0	1	0	0	0	0	0	0	0	0	0	0	1	1	0	0	0	0	1	1	37.5
2013_1	050664	0	1	0	1	0	0	0	0	0	0	1	0	0	0	1	0	1	0	1	1	0	0	1	0	33.3
2013_1	171262	1	0	1	0	0	0	0	1	0	0	1	0	0	0	0	0	0	1	1	0	1	0	0	0	29.2
2013_1	241262	1	1	0	1	1	1	1	1	0	1	1	0	1	0	0	0	1	1	0	0	1	0	0	1	58.3
2013_1	010264	1	1	1	1	1	0	1	1	0	1	1	0	0	0	0	0	1	0	1	0	1	0	0	0	50.0
2013_2	230469	0	1	1	1	1	0	0	1	0	0	0	0	1	1	0	0	0	0	1	0	0	1	0	0	37.5
2013_2	170268	1	1	1	0	0	0	0	1	0	0	1	0	0	0	1	0	0	0	1	1	0	0	0	1	37.5
2013_2	020666	1	1	1	1	1	0	0	1	0	0	1	0	0	0	1	1	0	0	1	0	1	0	0	0	45.8
2013_2	061160	1	0	1	1	1	1	0	0	1	1	1	1	1	0	0	0	1	1	1	1	0	0	0	0	58.3
2013_2	211263	1	0	1	1	1	1	1	1	0	1	1	1	1	1	0	0	1	1	1	1	0	1	0	1	75.0
2013_2	080765	0	1	1	1	0	0	0	1	0	1	0	0	0	0	0	0	0	0	0	0	0	0	0	0	20.8
2013_2	040761	0	1	1	1	0	1	0	1	0	0	0	0	0	0	0	0	0	1	1	0	0	1	0	0	33.3
2013_2	130163	1	1	0	1	0	0	0	1	0	1	1	0	1	1	1	0	1	0	0	0	0	1	0	1	50.0
2013_2	101263	1	1	1	1	1	0	1	1	0	1	1	0	1	1	1	0	0	0	1	0	1	1	1	0	66.7
2013_2	310560	0	1	0	0	0	1	0	1	0	0	0	0	0	0	0	1	1	0	0	0	0	0	0	0	20.8
2013_2	011068	0	1	1	1	1	0	1	1	1	0	1	0	0	0	1	0	0	1	0	0	0	0	0	0	41.7
2013_2	270961	0	1	1	1	0	0	0	1	0	0	1	0	0	0	0	0	0	1	1	0	0	0	0	0	29.2
2013_2	180460	0	1	1	1	0	0	0	1	0	1	1	0	0	1	0	0	0	1	1	1	1	0	0	0	45.8
2013_2	281063	0	1	0	0	0	1	1	1	0	0	1	0	0	0	0	1	1	0	0	1	0	0	0	0	33.3
2013_3	160294	1	1	1	1	1	1	1	1	0	1	0	1	0	1	1	0	1	1	1	0	0	1	1	1	75.0
2013_3	120662	1	1	1	1	1	0	0	0	0	1	1	1	0	1	1	0	1	1	0	1	0	0	0	0	54.2
2013_3	100658	1	1	1	1	1	0	1	1	0	0	0	1	0	0	1	0	0	1	1	0	0	0	1	0	50.0
2013_3	120254	1	0	1	1	1	0	1	0	0	1	0	1	0	1	0	0	0	0	0	0	0	0	1	0	37.5
2013_3	040569	1	1	1	1	1	1	0	0	0	1	0	0	0	1	0	0	0	1	0	0	1	1	0	1	50.0
2013_3	160369	1	0	1	1	0	0	1	0	0	1	1	0	0	0	0	0	0	1	1	0	1	0	0	1	41.7
2013_3	120264	1	1	1	1	1	1	1	1	0	1	0	1	0	1	1	1	1	0	1	1	0	1	1	1	79.2

2013_3	280165	0	1	1	1	1	1	1	1	0	1	0	1	0	1	1	0	0	1	1	0	0	0	0	1	58.3
2013_3	010259	1	1	1	1	0	1	0	0	0	1	0	1	0	1	0	0	0	1	1	0	0	0	0	0	41.7
2013_3	020271	1	1	0	1	0	1	1	1	0	0	0	0	0	1	0	1	1	0	1	0	1	0	0	1	50.0
2013_3	160267	1	1	1	1	1	0	1	1	0	1	0	0	0	1	1	1	1	0	1	0	0	0	0	0	54.2
2013_3	180958	0	1	0	1	1	0	1	1	0	1	0	0	0	0	1	0	0	1	1	0	1	0	0	0	41.7
2013_3	090956	1	0	0	1	1	0	0	1	0	0	1	1	0	0	0	0	0	0	1	1	0	0	1	0	37.5
2013_4	020463	1	1	1	1	1	0	1	1	0	1	0	1	1	1	0	0	0	0	1	0	1	0	0	0	54.2
2013_4	110962	1	1	1	1	0	0	1	1	0	1	0	1	1	1	0	0	1	1	0	0	1	0	0	0	54.2
2013_4	290653	1	1	1	1	0	1	1	0	0	1	1	1	1	0	1	0	0	0	1	0	1	0	0	0	54.2
2013_4	000000	1	1	1	1	1	0	1	1	0	0	1	1	1	1	0	0	1	1	0	1	1	0	0	0	62.5
2013_4	241163	0	0	1	1	0	1	1	1	0	1	1	1	0	0	0	0	0	1	0	0	0	0	0	0	37.5
2013_4	190567	0	1	1	1	0	0	1	1	0	1	0	0	0	0	0	0	1	1	0	0	1	0	0	0	37.5
2013_4	100764	1	0	1	1	0	1	0	1	1	1	0	1	1	0	0	0	0	1	0	0	1	0	1	0	50.0
2013_4	120567	1	1	1	1	1	1	1	0	1	1	1	1	1	1	1	1	1	0	1	1	1	1	1	1	91.7
2013_4	240961	1	1	1	1	0	0	1	1	0	1	1	1	1	1	0	0	0	1	1	0	0	1	0	0	58.3
2013_4	240961	1	1	1	1	0	0	1	0	0	1	1	1	1	1	1	0	0	1	1	0	0	0	0	1	58.3
2013_4	110263	1	1	1	1	0	0	1	1	0	1	1	1	1	1	1	0	0	1	1	0	1	0	0	0	62.5
2013_4	150561	1	1	0	1	0	1	1	1	0	1	0	1	1	1	1	0	0	0	1	0	0	0	0	0	50.0
2013_4	021266	0	1	1	1	1	1	1	0	0	1	0	1	1	1	1	0	0	1	1	1	1	1	0	1	70.8
2013_4	260264	1	1	1	1	0	0	1	1	0	1	0	0	0	1	0	0	1	0	1	0	1	0	0	1	50.0
2013_4	020764	1	1	1	1	1	1	1	0	0	0	1	1	0	1	1	0	1	1	1	0	1	1	1	0	70.8
2013_4	111260	1	1	1	1	1	0	1	1	0	0	1	1	0	0	0	0	0	1	1	1	0	0	0	0	50.0
2013_4	071257	0	1	1	0	0	0	1	1	0	1	1	1	0	0	1	0	0	1	0	1	0	0	0	0	41.7
2013_4	020964	1	1	1	0	0	0	1	1	0	0	1	1	1	1	1	0	1	0	1	1	0	0	0	0	54.2
2013_4	040961	1	1	1	0	0	1	1	1	0	1	1	0	1	0	0	0	1	1	1	0	0	0	0	0	50.0
2013_4	040457	1	1	1	1	1	1	1	1	0	1	0	1	0	1	0	1	0	1	1	0	0	0	1	0	62.5
2013_4	060672	1	1	1	1	0	1	1	1	1	0	1	1	0	1	1	0	1	1	1	1	0	0	1	1	75.0
2013_4	310156	1	1	0	1	0	0	1	0	1	1	1	0	0	1	1	0	1	1	0	0	1	0	0	1	54.2
2013_4	230456	1	1	1	1	0	1	1	1	0	1	1	0	1	1	0	0	1	0	1	0	0	0	0	0	54.2
2013_4	160763	1	1	0	1	1	0	1	1	0	1	1	0	1	1	0	0	1	1	1	0	0	0	0	0	54.2
2013_4	010965	0	1	1	0	1	0	1	1	0	1	0	1	0	0	0	0	0	1	1	0	0	0	0	0	37.5
2013_4	210194	1	1	0	1	0	1	1	1	0	0	0	0	1	0	0	0	1	0	1	0	1	1	0	0	45.8
2013_4	070961	1	1	1	1	0	0	1	0	0	1	1	1	0	1	0	0	0	1	0	0	0	0	0	0	41.7
2013_4	310566	1	1	0	1	0	0	1	1	0	0	1	0	1	1	0	0	0	1	0	0	1	0	0	0	41.7
2013_4	210164	1	1	0	1	0	0	1	1	0	0	0	0	0	1	0	0	1	0	0	0	1	0	0	0	33.3
2013_4	100167	0	1	0	0	1	1	0	1	1	0	0	0	0	0	0	0	1	0	1	0	0	0	0	0	29.2
2013_4	060566	1	1	0	1	1	0	1	1	0	1	1	1	1	1	0	0	1	1	0	1	0	0	0	0	58.3
2013_4	010260	1	1	1	1	0	1	1	1	0	1	0	1	1	1	0	0	1	0	1	1	1	0	0	0	62.5
2013_5	271167	0	1	1	1	0	1	1	0	0	1	1	1	0	1	0	0	1	0	0	1	1	0	1	0	54.2
2013_5	030759	0	1	1	1	1	0	1	0	1	1	1	1	1	0	1	0	0	0	0	0	0	0	1	0	50.0
2013_5	190370	0	1	1	1	0	1	1	1	0	1	1	1	0	1	0	0	0	0	0	0	1	0	0	0	45.8
2013_5	250561	1	1	1	1	1	0	1	1	0	1	1	1	0	1	0	1	1	1	0	1	0	0	0	0	62.5
2013_5	060563	1	1	0	1	1	1	1	1	0	1	1	1	0	1	1	0	0	1	0	1	1	0	0	1	66.7
2013_5	180257	0	0	0	1	1	0	0	0	0	1	0	1	0	0	0	0	1	0	0	0	0	0	0	0	20.8
2013_5	160264	1	1	0	1	0	0	1	0	0	1	0	1	0	1	0	0	1	1	1	1	0	0	0	0	45.8
2013_5	050163	1	1	1	0	0	0	1	0	0	0	1	1	0	1	0	0	0	1	0	0	0	0	0	0	33.3
2013_5	130457	1	0	0	1	0	0	0	1	1	1	0	0	0	1	0	0	0	1	0	0	0	0	0	0	29.2
2013_5	040454	0	0	1	1	1	1	1	1	0	0	0	1	0	1	0	0	1	1	1	0	0	0	1	0	50.0
2013_5	220668	1	0	1	1	1	0	1	0	0	1	1	1	0	0	0	0	1	0	1	0	0	0	0	0	41.7
2013_5	120165	1	1	0	1	1	0	1	0	0	1	1	0	0	1	0	0	1	1	0	1	0	0	0	0	45.8
2013_5	310158	0	1	1	1	0	1	1	0	1	1	1	0	0	1	0	0	1	1	1	1	0	0	1	0	58.3
2013_5	150256	1	0	0	1	0	0	0	0	0	1	1	0	0	1	0	0	0	1	1	0	1	0	0	0	33.3
2013_5	120864	0	1	0	1	1	0	1	1	0	1	1	0	0	1	0	0	1	1	1	0	0	0	0	0	45.8
2013_5	210467	1	1	0	1	1	0	1	1	0	1	1	1	0	1	0	0	0	1	1	0	1	0	1	0	58.3
2013_5	151163	1	1	0	1	1	1	1	1	0	0	0	1	0	1	1	0	1	0	0	0	0	0	0	0	45.8
2013_5	301000	1	0	1	1	0	0	0	0	0	0	1	0	1	1	0	1	0	0	0	0	0	0	0	1	33.3
2013_5	120266	0	1	0	1	1	1	1	1	0	1	1	0	0	0	0	0	1	1	0	0	1	0	0	0	45.8
2013_5	100369	0	1	1	1	1	1	0	1	0	0	0	0	0	0	1	0	0	0	0	0	0	0	0	0	29.2
2013_5	250664	0	1	0	1	1	0	1	0	1	0	1	0	1	1	0	0	1	1	1	0	0	0	0	0	45.8
2013_6	221157	1	0	1	1	1	0	1	1	0	1	1	1	0	1	0	0	1	1	1	0	0	0	0	0	54.2

2013_6	180768	1	0	0	1	0	1	1	1	0	1	1	1	0	1	1	1	0	1	1	0	1	0	0	1	62.5
2013_6	180266	1	1	1	1	1	1	1	0	1	1	0	1	0	1	0	0	1	1	1	1	0	0	0	1	66.7
2013_6	160563	1	1	1	1	1	0	0	1	0	0	0	0	0	0	0	0	1	1	1	1	0	0	0	1	45.8
2013_6	111059	0	1	0	0	0	0	1	1	0	0	0	1	0	0	0	0	1	0	1	0	1	0	0	0	29.2
2013_6	101159	1	1	0	1	0	0	0	0	0	0	1	0	0	1	0	0	0	1	0	0	0	0	0	0	25.0
2013_6	050559	1	1	0	1	0	0	1	1	0	0	1	1	1	1	1	0	1	0	1	1	1	1	0	1	66.7
2013_7	081153	1	1	1	1	1	0	1	0	0	0	1	1	0	1	0	0	0	0	1	0	0	0	1	0	45.8
2013_7	220464	1	1	1	1	1	0	1	1	0	1	1	1	0	1	1	0	0	0	1	0	0	0	1	0	58.3
2013_7	280366	1	1	1	1	0	0	1	0	0	1	1	1	0	1	1	0	0	0	1	1	0	0	0	0	50.0
2013_7	121263	1	0	0	1	1	1	1	0	0	1	1	0	0	0	0	0	0	0	1	0	1	0	0	0	37.5
2013_7	071257	1	1	1	1	0	0	1	1	0	1	0	0	0	0	1	0	1	0	1	0	0	0	0	0	41.7
2013_7	180666	1	1	1	0	0	0	1	0	0	1	1	1	0	1	0	0	1	0	1	0	0	0	0	0	41.7
2013_7	061164	1	1	1	0	1	0	1	1	0	0	1	0	0	1	0	0	0	1	0	0	0	0	0	0	37.5
2013_7	261166	1	0	1	1	1	1	1	1	0	0	0	0	0	1	0	1	0	1	0	0	0	0	0	1	45.8
2013_7	070363	1	0	1	1	1	1	1	1	0	0	0	0	0	1	0	0	0	0	1	0	0	0	0	1	41.7
2013_7	040866	1	0	1	1	0	0	0	1	0	1	0	0	0	0	1	0	1	0	1	0	0	0	0	0	33.3
2013_7	060165	1	1	1	1	1	0	1	0	0	1	1	1	0	1	0	0	0	1	0	0	0	0	1	0	50.0
2013_7	180458	1	1	1	1	0	0	1	0	0	0	0	0	0	1	1	0	0	0	1	0	0	0	0	1	37.5
2013_7	011063	1	1	1	1	1	0	1	1	0	0	1	1	0	0	0	0	0	0	0	0	0	1	0	0	41.7
2013_7	171061	1	1	1	1	1	0	1	1	0	0	1	1	0	0	1	0	1	1	0	0	1	1	0	0	58.3
2013_7	211168	0	0	1	0	0	0	1	1	0	0	1	1	0	0	0	0	0	0	1	0	0	0	0	1	29.2
2013_7	200458	1	1	1	0	1	0	1	0	0	1	1	1	0	1	0	0	0	0	1	0	1	0	0	1	50.0
2013_8	010969	1	1	1	1	0	1	0	1	0	1	0	1	0	1	1	0	0	0	1	0	0	1	1	1	58.3
2013_8	281262	1	1	0	1	0	1	1	1	0	0	0	1	0	0	0	0	0	1	1	0	0	0	0	0	37.5
2013_8	280268	1	1	0	0	0	0	1	0	0	1	0	1	1	1	0	0	0	1	1	0	1	0	0	1	45.8
2013_8	300161	1	1	1	1	0	0	1	0	0	1	0	0	1	1	0	0	1	0	0	0	1	0	0	1	45.8
2013_8	040964	0	1	0	1	0	0	0	1	0	0	1	1	0	1	1	0	1	1	0	1	0	0	0	1	45.8
2013_8	000001	0	1	1	0	0	1	1	1	0	0	0	1	1	0	0	0	1	0	1	0	0	1	0	0	41.7
2013_8	250463	0	1	1	1	1	1	1	1	0	0	0	0	1	1	1	0	1	1	0	0	0	1	0	1	58.3
2013_8	060862	1	1	0	1	1	1	1	0	0	0	0	1	0	1	0	0	0	1	1	0	0	0	0	0	41.7
2013_8	020660	0	1	1	1	0	1	1	0	0	1	1	1	0	0	0	0	0	1	1	0	1	0	0	1	50.0
2013_8	150562	1	1	1	1	0	0	1	0	0	1	1	1	1	1	1	0	0	0	1	1	0	0	1	0	58.3
2013_8	130958	1	1	1	1	1	0	0	1	0	0	0	1	0	1	1	1	1	1	1	0	0	0	0	1	58.3
2013_8	050166	1	1	0	1	0	0	1	0	0	0	0	1	1	1	0	0	0	0	1	0	0	0	0	1	37.5
2013_8	000002	1	1	0	1	0	0	0	0	0	1	0	1	1	1	0	0	0	0	1	0	0	0	0	0	33.3
2013_8	151066	1	1	1	1	0	0	1	0	0	1	0	1	1	1	0	0	0	1	1	0	0	0	0	0	45.8
2013_8	310165	0	1	1	1	1	1	0	1	0	1	0	1	0	0	0	0	0	1	1	0	0	0	0	0	41.7
2013_8	050770	1	1	1	1	0	1	1	0	1	1	0	1	1	1	1	0	1	0	1	0	0	0	1	1	66.7
2013_9	290458	0	1	0	1	0	0	0	1	0	1	0	0	0	0	1	1	1	0	1	0	0	0	0	0	33.3
2013_9	291254	1	0	0	1	1	0	1	0	0	1	0	1	0	1	0	1	0	0	1	0	1	0	0	1	45.8
2013_9	150767	1	1	1	1	1	1	1	1	0	1	1	1	1	1	0	1	1	0	1	0	1	0	0	0	70.8
2013_9	190657	0	1	1	1	0	0	1	1	0	1	0	1	0	1	1	0	0	0	0	0	0	0	0	0	37.5
2013_9	091066	1	0	1	1	0	1	1	1	0	0	0	1	0	0	1	1	1	0	1	1	1	0	0	0	54.2
2013_9	121057	1	0	0	1	1	0	1	0	0	0	0	0	0	0	0	1	0	1	0	0	0	0	0	1	29.2
2013_9	060565	0	1	0	1	0	1	1	0	0	0	1	1	0	1	0	0	1	1	1	0	1	0	1	0	50.0
2013_9	029957	1	1	1	1	0	0	1	1	0	1	1	1	1	1	0	0	1	0	0	0	1	0	0	0	54.2
2013_9	160859	1	0	1	1	0	0	1	1	0	1	0	1	1	0	1	0	0	0	0	0	0	0	1	1	45.8
2013_9	110962	1	1	0	1	0	0	0	1	0	1	0	1	0	0	0	0	0	1	0	0	0	0	0	0	29.2
2013_9	110264	0	1	0	1	1	0	0	0	0	1	0	0	1	0	0	0	0	0	1	0	0	0	0	0	25.0
2013_9	230665	1	1	0	1	0	0	1	1	0	0	0	1	0	0	0	0	0	1	1	0	0	0	0	0	33.3
2013_9	090958	0	0	0	1	0	1	0	0	0	1	0	0	1	0	1	0	0	0	1	0	0	0	0	0	25.0
2013_9	211062	1	0	1	1	1	0	0	0	0	0	0	1	0	0	1	1	0	0	1	1	1	0	0	0	41.7
2013_9	270764	1	1	0	1	1	0	0	0	0	1	0	1	0	1	1	1	0	1	1	0	0	0	1	0	50.0
2013_10	290160	1	0	1	1	1	1	1	1	0	1	0	1	0	0	0	0	1	0	0	0	0	0	0	0	41.7
2013_10	130165	1	1	1	1	0	1	0	1	0	0	0	1	0	0	0	0	0	0	1	0	0	0	0	0	33.3
2013_10	201167	1	1	1	1	1	0	1	1	0	1	1	1	1	1	0	0	1	1	0	0	0	0	0	1	62.5
2013_10	091064	1	1	0	1	0	1	1	1	0	1	1	1	0	1	0	0	0	0	1	0	1	0	0	0	50.0
2013_10	160362	1	1	1	1	1	1	0	1	0	1	1	1	0	0	0	0	0	0	0	1	0	0	0	1	50.0
2013_10	171166	1	1	1	1	0	1	1	1	0	0	1	1	0	1	0	0	0	0	1	0	0	1	1	1	58.3
2013_10	011153	1	1	1	1	1	0	1	1	0	1	0	1	0	1	0	1	0	0	0	0	0	0	0	0	45.8

2013_10	290856	0	1	0	0	0	0	0	0	0	1	1	0	0	0	0	0	0	0	0	0	1	0	0	0	16.7
2013_10	031061	1	1	1	1	0	0	0	1	0	0	0	1	0	1	1	0	1	1	1	0	0	0	0	0	45.8
2013_10	100461	1	0	0	0	0	1	1	1	1	1	0	1	0	0	0	0	0	0	1	1	0	0	0	0	37.5
2013_10	050459	1	0	1	1	1	0	1	0	0	0	0	1	0	1	0	0	0	0	0	1	1	0	1	0	41.7
2013_10	110562	1	1	1	1	1	1	1	1	0	1	0	1	1	1	0	1	1	0	1	1	1	0	0	1	75.0
2013_10	080264	1	1	1	1	0	0	0	1	0	0	0	1	0	1	1	0	0	1	1	0	1	0	0	0	45.8
2013_10	150659	1	1	1	1	0	1	1	1	0	0	1	1	0	0	0	0	0	0	1	0	0	0	0	1	45.8
2013_11	150862	1	1	0	1	1	1	1	1	0	0	0	1	0	0	0	0	1	1	0	0	1	0	0	0	45.8
2013_11	181160	1	1	1	1	0	0	1	1	0	0	0	0	0	0	0	0	0	1	1	0	0	1	1	0	41.7
2013_11	250358	1	1	1	1	0	0	0	1	0	1	0	1	0	0	1	0	1	0	0	0	1	0	0	0	41.7
2013_11	250657	1	0	1	1	0	0	1	1	0	0	0	1	0	0	0	0	0	1	1	0	0	1	0	1	41.7
2013_11	140865	1	0	1	1	0	0	1	0	0	1	0	0	0	0	1	1	0	1	1	0	0	0	0	1	41.7
2013_11	310865	0	0	1	0	0	1	1	1	0	1	1	1	0	0	1	1	1	1	1	0	0	0	0	1	54.2
2013_11	190862	1	0	1	1	1	1	1	1	0	0	0	1	1	1	1	1	0	1	1	1	0	0	0	1	66.7
2013_11	240263	1	0	0	1	0	0	1	1	0	0	0	1	0	0	0	0	1	0	1	0	0	0	0	0	29.2
2013_12	231160	0	1	0	1	1	1	1	0	0	1	0	0	0	0	0	1	0	1	0	0	0	0	0	0	33.3
2013_12	020967	0	1	1	1	0	0	1	0	0	1	1	1	1	0	0	1	0	0	0	1	0	0	1	0	45.8
2013_12	220864	1	1	1	1	0	1	1	1	0	1	1	1	0	0	0	0	0	1	0	1	0	0	0	0	50.0
2013_12	280770	1	1	1	1	0	1	1	1	0	0	1	0	0	0	1	0	0	0	0	0	0	0	0	0	37.5
2013_12	130463	0	1	0	1	1	1	1	1	0	0	1	1	0	0	0	0	0	0	0	0	0	0	0	1	37.5
2013_12	300369	0	1	1	1	0	0	0	1	0	1	0	1	0	0	0	0	1	1	0	0	1	1	0	0	41.7
2013_12	170465	1	1	1	1	0	1	0	0	0	1	0	1	0	0	0	1	0	1	1	0	1	0	0	0	45.8
2013_12	260163	1	1	1	1	0	1	1	1	0	1	0	0	0	0	0	0	0	0	1	0	1	0	0	0	41.7
2013_12	060965	1	1	0	0	0	0	1	1	0	0	1	1	1	1	0	0	0	1	0	0	1	0	1	0	45.8
2013_12	090766	0	0	1	1	0	1	0	0	0	1	0	0	0	0	1	1	0	0	0	0	0	0	0	1	29.2
2013_12	101960	1	1	1	1	0	0	1	0	0	1	1	1	0	1	0	0	0	1	0	0	0	0	0	0	41.7
2013_12	241163	0	0	1	1	0	0	1	1	0	1	1	1	1	0	1	1	1	0	1	0	0	0	0	1	54.2
2013_12	100764	0	1	1	0	0	0	1	1	0	1	0	1	0	0	0	0	0	0	0	0	0	0	0	0	25.0
2013_12	020262	0	1	0	1	0	0	1	1	0	1	0	0	0	0	1	0	0	0	0	1	1	1	0	0	37.5
2013_12	080962	0	0	0	1	0	1	1	1	0	1	1	1	1	0	0	0	1	1	1	0	0	0	0	0	45.8
2013_12	220768	1	0	1	1	0	0	0	1	0	1	0	1	0	0	0	0	0	1	0	0	1	0	0	0	33.3
2013_12	260560	1	1	0	1	0	1	0	0	0	0	0	0	0	0	1	0	0	0	0	1	1	0	0	0	29.2
2013_12	150169	1	1	0	1	0	0	1	0	0	0	0	1	0	0	1	0	0	0	0	0	1	0	0	0	29.2
		70.1	79.1	69.2	89.1	41.2	39.8	73.5	66.8	7.1	59.2	46.0	58.3	26.1	50.7	31.8	17.1	38.4	52.6	60.7	23.2	34.1	11.8	19.0	26.1	

Table 3: Performance of students (cohort 2014) to select the best answer on all BCI questions

Group	Student ID	Q1	Q2	Q3	Q4	Q5	Q6	Q7	Q8	Q9	Q10	Q11	Q12	Q13	Q14	Q15	Q16	Q17	Q18	Q19	Q20	Q21	Q22	Q23	Q24	Score
2014_1	051165	1	1	0	1	1	0	1	0	1	0	0	1	0	0	0	0	0	0	1	0	1	0	1	0	41.7
2014_1	310161	1	1	1	1	0	0	1	1	0	1	1	0	0	1	0	0	0	0	1	1	0	1	0	1	54.2
2014_1	030869	1	1	0	1	1	0	1	0	0	1	1	0	0	0	0	0	0	1	1	0	1	1	0	0	45.8
2014_1	151063	1	1	1	0	1	0	0	0	0	0	1	0	0	1	1	1	0	1	1	0	0	0	0	0	41.7
2014_1	200864	1	1	1	0	0	0	1	1	0	0	0	0	1	0	0	0	0	0	1	0	0	1	0	0	33.3
2014_1	160669	1	0	1	0	0	0	1	1	0	0	1	1	0	0	0	0	1	1	1	0	0	0	0	0	37.5
2014_1	021264	1	1	1	1	0	1	1	0	0	0	0	0	0	0	1	0	1	1	1	0	0	0	0	0	41.7
2014_1	101263	1	1	1	1	0	1	0	0	1	0	0	1	0	0	0	0	1	0	0	0	1	0	0	0	37.5
2014_1	250860	1	0	0	1	0	0	1	0	0	0	0	1	0	0	0	0	1	1	1	0	0	0	0	0	29.2
2014_1	010363	1	1	1	1	0	1	1	1	0	1	0	0	0	1	0	0	1	1	1	0	0	0	0	0	50.0
2014_1	101062	0	1	1	0	0	0	1	1	0	0	0	0	0	0	0	0	1	0	0	0	0	0	1	0	25.0
2014_1	120467	1	1	0	1	0	0	1	1	0	0	0	1	1	0	0	0	0	0	1	0	1	0	1	0	41.7
2014_1	130767	0	0	1	1	0	0	0	0	0	1	0	0	0	0	1	0	1	0	0	0	1	0	0	0	25.0
2014_1	050567	1	1	1	1	0	0	0	0	0	0	0	0	0	0	0	0	0	0	0	0	0	0	0	0	16.7
2014_1	200962	1	1	0	1	0	1	0	1	0	1	1	0	0	1	1	0	0	0	1	0	0	0	0	1	45.8
2014_1	050265	0	1	0	1	0	1	1	0	0	1	0	0	0	1	0	0	0	1	1	0	0	1	0	0	37.5
2014_1	150263	1	0	1	1	0	1	1	1	0	1	1	1	0	0	0	1	0	1	1	0	1	0	0	0	54.2
2014_2	100663	1	0	0	1	1	1	1	1	0	1	1	1	0	1	0	0	0	0	1	1	1	0	1	0	58.3
2014_2	231067	1	1	0	1	1	0	1	1	0	1	0	1	0	0	0	0	0	0	1	0	0	0	0	0	37.5
2014_2	050660	0	1	0	1	0	1	1	1	0	1	0	1	0	0	0	0	0	0	0	0	0	0	0	1	33.3
2014_2	070759	1	1	0	1	0	0	0	1	0	1	0	0	1	0	0	0	0	0	1	0	0	0	1	0	33.3
2014_2	130165	1	1	1	0	1	0	1	0	0	1	1	0	0	1	1	0	0	0	1	0	0	0	0	0	41.7
2014_2	191159	1	0	0	0	0	0	0	1	0	0	0	1	1	1	0	1	0	1	0	0	1	1	0	0	37.5
2014_2	231056	0	1	1	1	0	1	1	1	0	0	0	1	0	0	0	0	0	0	1	0	0	0	0	0	33.3
2014_2	060256	0	1	1	0	0	0	0	0	0	0	0	0	0	0	0	0	0	0	1	1	0	1	1	1	29.2
2014_2	050867	1	1	0	1	0	1	1	1	0	0	1	0	0	0	0	0	1	0	1	0	1	0	0	1	45.8
2014_2	041167	1	1	0	1	1	0	0	1	0	1	0	0	0	0	0	0	0	0	1	0	0	0	0	1	33.3
2014_2	250865	0	1	0	1	1	0	1	0	0	1	1	0	0	1	1	0	1	1	1	0	1	0	1	1	58.3
2014_2	231161	1	1	0	1	1	0	1	0	0	1	0	1	1	0	0	0	1	0	1	0	0	0	0	0	41.7
2014_2	201261	1	0	0	1	0	1	1	1	0	1	1	0	0	0	1	0	0	1	1	0	0	0	0	0	41.7
2014_2	031262	1	0	1	1	1	1	0	1	0	1	1	1	1	1	0	0	1	1	1	1	0	0	1	1	70.8
2014_2	060964	1	0	0	1	1	0	0	1	0	0	0	0	0	0	0	0	1	0	1	0	0	1	0	0	29.2
2014_2	260868	0	1	0	1	0	0	1	0	0	1	0	0	1	0	0	0	1	0	1	0	0	0	0	0	29.2
2014_2	NA	1	1	1	1	0	0	1	1	0	1	1	0	1	1	0	0	1	0	1	0	1	0	0	0	54.2
2014_2	250363	1	1	0	1	0	0	0	0	1	0	0	0	0	0	0	0	1	0	0	0	0	0	0	0	20.8
2014_2	020768	1	1	0	1	0	1	1	1	0	1	0	1	0	0	0	0	0	0	1	0	1	1	0	1	50.0
2014_2	151064	1	1	1	1	1	1	1	0	0	1	1	0	0	0	0	0	1	0	0	0	0	0	0	0	41.7
2014_2	230158	1	0	1	1	0	1	1	1	0	1	1	1	0	1	0	0	1	0	1	0	1	0	0	0	54.2
2014_2	150959	1	1	0	1	1	1	1	0	0	1	1	0	0	0	0	0	0	0	0	1	0	0	0	0	37.5
2014_2	281066	1	1	1	1	1	1	1	1	0	1	1	0	0	1	0	0	1	1	0	1	1	0	0	0	62.5
2014_2	100760	0	0	1	1	0	1	1	0	0	1	1	0	0	1	1	0	1	0	1	0	1	0	1	0	50.0
2014_2	170261	0	1	1	0	1	1	1	1	0	0	0	0	0	0	0	0	1	1	0	0	0	0	1	0	37.5
2014_2	010250	0	1	0	1	0	1	1	1	0	1	1	1	0	1	0	0	1	1	1	0	0	0	0	0	50.0
2014_2	200263	1	1	0	1	0	0	1	1	0	1	1	0	0	0	0	0	0	0	0	0	1	0	0	1	37.5
2014_2	150766	1	1	1	1	0	1	1	0	0	0	0	0	1	1	0	0	1	1	1	0	0	0	0	0	45.8
2014_2	240360	0	1	0	1	0	0	1	1	1	1	0	1	0	1	0	0	1	1	1	1	0	0	1	1	58.3
2014_2	020752	1	0	0	1	0	1	1	1	0	1	0	0	0	1	0	0	0	1	1	0	1	0	1	0	45.8
2014_2	021063	1	1	1	1	0	0	0	1	0	1	1	0	0	1	0	0	0	1	1	0	0	0	0	0	41.7
2014_2	030859	0	1	1	1	0	1	1	0	0	0	0	1	0	0	0	1	1	1	1	0	0	1	1	0	50.0
2014_2	030869	0	1	1	1	1	0	1	1	0	1	1	0	1	1	1	0	0	0	1	0	0	0	0	0	50.0
2014_2	260265	1	1	0	1	1	1	1	1	1	1	1	1	0	1	0	0	1	1	1	1	0	0	0	0	66.7
2014_2	011258	0	1	1	1	1	0	1	0	0	1	0	1	0	1	0	0	1	1	0	0	1	1	0	0	50.0
2014_2	190370	1	1	0	1	1	1	1	1	0	0	0	0	1	0	0	0	0	0	0	0	1	1	0	0	41.7
2014_2	100468	1	0	1	1	1	0	0	0	0	0	0	0	0	0	1	0	0	0	0	0	0	1	0	0	25.0

2014_2	280264	1	1	1	0	0	1	1	0	0	0	0	0	0	0	1	1	1	0	1	0	0	0	0	0	37.5
2014_2	280864	0	1	1	0	0	1	1	1	0	1	1	1	0	0	0	0	1	0	0	1	0	0	0	0	41.7
2014_2	080864	0	1	1	1	0	0	1	1	0	1	0	0	0	1	1	0	1	1	1	1	0	0	0	0	50.0
2014_2	NA	1	0	0	1	0	0	0	1	0	0	0	1	0	1	1	0	0	0	1	1	0	0	0	1	37.5
2014_2	030463	1	1	0	1	0	0	1	1	0	1	1	0	1	0	0	0	0	1	0	0	1	0	1	1	50.0
2014_2	090662	1	0	1	1	1	1	1	1	0	1	0	0	0	1	0	1	1	0	0	1	0	0	0	1	54.2
2014_2	010364	1	1	0	1	0	0	1	0	0	1	0	0	0	0	0	0	1	1	1	1	0	0	0	0	37.5
2014_2	151256	1	1	0	1	0	1	1	0	0	1	1	1	0	0	0	0	0	0	0	0	0	0	0	0	33.3
2014_3	180471	1	1	1	1	1	0	1	0	0	0	1	1	0	1	1	0	0	1	1	0	1	0	0	0	54.2
2014_3	010462	0	0	1	0	0	0	0	0	1	0	1	1	1	1	1	1	0	0	1	0	1	0	0	0	41.7
2014_3	230163	1	0	1	1	0	0	0	0	0	0	0	0	0	1	1	1	1	0	0	0	0	0	1	0	33.3
2014_3	200467	1	0	0	1	0	1	1	0	0	1	0	0	1	0	0	0	1	1	1	0	0	0	0	0	37.5
2014_3	201163	1	1	1	1	1	0	1	0	0	0	0	0	1	0	0	0	0	1	0	0	1	0	0	0	37.5
2014_3	080964	1	1	0	1	0	1	1	1	0	1	0	1	1	1	0	1	0	1	0	0	1	0	0	1	58.3
2014_3	010867	0	1	0	1	0	1	0	1	0	0	0	0	1	0	0	0	0	1	1	0	0	0	0	0	29.2
2014_3	261056	1	1	1	1	0	1	1	1	0	1	0	1	1	1	0	0	0	0	1	0	0	0	0	1	54.2
2014_3	010256	1	1	1	1	0	0	1	1	0	1	1	0	0	1	0	0	0	1	1	1	0	1	1	1	62.5
2014_3	171263	1	1	1	1	0	1	1	0	0	1	0	0	1	0	0	0	0	0	1	0	0	0	0	0	37.5
2014_3	181267	1	1	0	0	0	1	1	0	1	0	0	0	1	1	0	0	0	0	0	0	1	1	0	0	37.5
2014_3	130262	1	1	1	1	1	0	1	0	0	0	0	1	1	0	1	0	0	1	0	0	1	1	0	1	54.2
2014_3	090266	1	0	0	1	1	0	0	0	0	1	0	0	0	0	1	1	0	0	0	1	1	0	0	1	37.5
2014_3	011062	1	1	1	1	0	1	0	1	0	0	0	0	0	0	1	0	1	0	1	0	1	0	0	1	45.8
2014_3	300470	1	0	1	0	1	0	1	0	0	1	0	0	0	0	0	0	1	0	0	1	0	0	1	0	33.3
2014_3	270464	0	1	1	1	0	1	1	0	0	0	0	0	0	1	0	0	1	0	0	1	1	1	0	0	41.7
2014_3	141258	0	0	0	1	0	1	1	0	0	0	0	0	0	1	0	0	0	0	0	0	1	0	0	0	20.8
2014_3	190273	1	1	0	0	0	1	0	0	1	0	1	0	0	0	1	0	0	0	0	0	1	0	0	1	33.3
2014_3	030259	1	1	1	0	0	1	1	1	0	0	0	1	0	0	1	0	0	1	0	0	0	0	1	0	41.7
2014_3	181161	1	1	1	0	0	1	1	0	0	1	0	0	0	0	1	0	1	0	1	0	0	0	0	1	41.7
2014_4	261171	1	1	1	1	1	1	1	0	0	1	1	1	0	0	0	0	0	0	1	0	0	1	0	0	50.0
2014_4	290964	1	1	1	0	0	0	1	0	0	0	0	0	1	0	0	0	0	0	1	0	0	1	0	0	29.2
2014_4	040266	1	0	1	0	0	0	1	1	0	0	1	1	0	0	1	0	1	1	1	0	0	0	0	1	45.8
2014_4	160358	1	1	1	1	0	0	1	1	0	0	0	1	0	0	1	0	0	0	0	0	0	0	1	0	37.5
2014_4	190463	1	1	1	1	1	1	1	1	0	0	0	1	0	0	1	0	0	0	0	0	1	0	1	0	50.0
2014_4	170659	1	1	1	1	0	0	1	1	0	1	0	1	0	0	0	0	0	1	1	1	0	0	1	0	50.0
2014_4	300365	0	1	1	1	1	0	1	1	0	1	0	1	0	0	1	0	0	0	0	0	1	0	0	0	41.7
2014_4	091162	1	1	1	1	0	0	1	1	0	1	1	1	1	1	0	0	0	1	0	0	1	0	0	0	54.2
2014_4	220766	1	1	1	1	0	0	1	1	0	1	0	1	0	0	0	0	0	1	1	1	0	0	1	0	50.0
2014_4	051066	1	1	1	0	0	0	1	0	0	1	0	1	0	0	0	0	0	1	0	1	0	0	1	0	37.5
2014_4	190159	1	1	0	1	1	0	1	0	0	1	0	0	0	1	1	0	1	1	1	1	0	0	1	0	54.2
2014_4	210260	0	0	1	1	0	0	0	0	0	0	1	1	0	1	1	0	1	0	0	0	0	0	1	0	33.3
2014_4	NA	0	0	0	1	1	0	0	1	0	0	0	0	0	1	0	0	0	0	1	0	0	0	0	0	20.8
2014_4	260854	1	1	0	1	0	0	0	1	0	0	0	0	0	1	1	0	0	0	1	1	0	0	0	0	33.3
2014_4	260664	1	1	0	1	0	1	0	0	0	1	1	1	0	0	0	0	0	0	0	0	0	0	0	0	29.2
2014_5	120773	1	1	0	1	0	1	1	1	0	0	1	1	0	1	1	0	1	1	1	0	0	0	0	1	58.3
2014_5	141167	1	1	1	1	0	1	0	1	0	1	1	1	0	0	1	0	1	1	0	0	0	0	0	1	54.2
2014_5	151264	1	0	1	0	0	0	1	1	0	1	1	0	1	1	0	0	0	1	1	0	1	0	0	0	45.8
2014_5	111063	1	1	0	1	0	0	0	1	0	0	0	0	0	0	1	0	0	0	1	0	1	1	0	0	33.3
2014_5	060262	1	1	1	1	0	0	1	1	0	1	1	0	0	1	0	0	1	1	0	1	0	0	0	1	54.2
2014_5	140965	1	0	0	1	0	0	1	0	0	1	0	0	0	1	1	0	1	1	1	1	0	0	0	0	41.7
2014_5	290862	1	1	1	1	1	1	0	0	0	1	1	1	1	0	0	0	1	0	1	1	0	0	0	0	54.2
2014_5	260867	1	1	1	1	0	0	1	1	0	1	1	1	0	1	1	0	0	1	1	1	1	0	0	0	62.5
2014_5	040363	1	1	0	1	1	0	1	0	0	0	1	1	0	1	0	0	1	0	1	0	0	0	0	0	41.7
2014_5	140264	1	1	1	1	0	0	1	1	0	0	0	0	0	1	0	0	1	0	0	1	0	1	0	1	45.8
2014_6	221267	1	0	1	1	1	0	1	1	0	1	1	0	0	1	0	0	0	1	1	1	0	0	0	0	50.0
2014_6	311064	0	1	0	1	1	0	1	1	0	0	0	0	0	0	0	0	0	1	1	0	1	0	0	0	33.3
2014_6	281168	0	1	1	0	1	0	1	1	0	1	1	1	0	1	1	0	0	0	1	0	0	0	0	1	50.0

2014_6	250263	0	1	1	1	1	1	1	0	0	1	1	1	0	1	0	1	1	1	1	0	0	0	0	0	58.3
2014_6	221155	0	1	1	1	1	1	1	1	0	0	0	1	0	1	1	0	1	0	1	0	0	0	0	0	50.0
2014_6	151059	1	1	1	1	1	0	1	0	0	1	1	1	0	1	0	0	1	1	1	1	1	0	0	0	62.5
2014_6	181264	0	0	1	1	1	0	1	1	0	0	0	1	0	0	0	0	0	0	1	0	1	0	0	0	33.3
2014_6	020257	0	0	0	1	0	0	1	1	0	0	0	0	1	0	0	0	1	0	1	0	0	0	0	0	25.0
2014_6	070369	0	1	1	1	1	0	1	1	0	1	0	1	0	1	0	0	1	0	1	0	0	1	0	0	50.0
2014_6	060864	1	1	1	0	1	0	1	0	0	1	1	0	0	1	0	0	0	0	1	1	0	0	0	0	41.7
2014_6	030959	0	1	1	1	1	1	1	1	0	1	1	1	0	1	0	0	1	1	0	1	1	0	0	0	62.5
2014_6	170765	0	1	1	1	1	1	1	1	0	1	1	1	1	0	0	0	1	0	0	0	0	0	0	0	50.0
2014_7	070856	1	1	1	1	0	0	1	1	0	1	0	0	0	0	0	0	1	1	0	0	0	0	0	1	41.7
2014_7	230569	0	1	1	1	0	1	1	1	0	1	0	0	0	0	0	0	1	1	0	0	0	0	0	0	37.5
2014_7	150263	1	0	1	0	0	0	1	0	0	1	0	0	0	0	1	0	0	0	1	0	0	0	0	1	29.2
2014_7	030475	0	0	0	1	0	0	1	1	0	1	0	0	1	0	0	0	0	0	0	0	0	0	0	0	20.8
2014_7	200666	0	0	1	1	0	0	1	1	0	1	0	0	0	0	0	0	0	0	0	0	1	0	0	0	25.0
2014_7	210855	1	0	1	1	0	1	1	1	0	1	0	0	0	1	0	0	1	1	0	0	0	0	0	0	41.7
2014_7	120464	0	1	0	1	0	1	0	1	0	0	0	0	0	0	0	0	1	1	1	0	0	0	1	0	33.3
2014_7	070664	1	0	1	1	0	0	1	1	0	0	0	0	0	0	0	0	0	0	0	0	0	0	1	0	25.0
2014_7	070367	1	0	1	1	1	1	0	1	0	0	0	1	0	0	0	0	0	1	0	0	0	0	1	0	37.5
2014_7	020966	1	0	1	1	0	0	1	1	0	1	0	0	0	0	0	0	0	0	0	0	0	0	0	0	25.0
2014_7	200770	1	1	1	1	0	1	1	0	0	0	0	0	0	0	0	0	0	0	0	0	0	0	0	0	25.0
2014_7	260866	1	1	1	1	0	1	1	1	0	1	1	0	0	0	1	0	0	1	0	0	1	1	0	1	58.3
2014_7	250470	1	1	1	1	0	0	1	1	0	1	1	0	1	0	1	0	0	1	0	0	1	1	0	0	54.2
2014_7	080860	1	1	1	1	0	1	1	1	0	1	0	0	0	0	0	0	0	0	0	0	1	0	1	0	41.7
2014_7	080662	1	1	1	1	1	1	1	0	0	1	0	0	0	0	1	1	0	0	0	0	0	0	0	0	41.7
2014_7	200364	1	1	1	1	1	1	1	1	0	1	0	0	0	0	1	0	0	0	0	0	0	1	1	0	50.0
2014_7	280464	0	1	1	1	1	0	0	1	0	0	1	0	0	0	0	0	0	0	0	0	1	0	1	0	33.3
2014_7	NA	1	0	0	1	0	0	1	1	0	1	1	0	0	1	0	0	1	1	1	0	1	0	0	0	45.8
2014_7	110571	1	1	1	0	0	0	1	0	0	0	0	1	0	0	0	0	0	1	1	0	1	0	0	0	33.3
2014_7	NA	1	1	1	1	0	0	1	0	0	1	1	1	0	0	0	1	0	0	1	0	0	0	1	0	45.8
2014_7	100664	1	1	1	1	0	0	1	0	0	1	1	1	0	0	0	1	0	0	0	0	0	0	0	0	37.5
2014_7	060472	1	1	0	1	0	1	1	1	0	0	1	0	1	1	0	0	0	0	0	1	1	0	0	0	45.8
2014_7	220177	1	1	1	1	0	0	1	0	0	1	1	1	0	0	0	1	0	0	0	0	0	0	0	0	37.5
2014_7	090161	1	1	1	1	0	0	1	0	0	1	1	1	0	0	0	1	0	0	0	0	0	0	0	0	37.5
2014_7	241163	1	1	0	1	0	1	1	1	0	0	1	0	0	0	1	0	0	1	0	0	1	0	0	0	41.7
2014_7	140369	1	1	0	1	0	1	0	0	0	1	0	0	0	0	0	0	0	0	1	0	1	0	0	0	29.2
2014_7	160661	1	1	0	1	0	1	1	0	0	1	1	0	0	0	0	0	0	0	1	0	1	0	0	0	37.5
2014_7	141062	1	1	0	1	0	1	0	0	0	1	0	0	0	0	0	0	0	0	1	0	1	0	0	0	29.2
2014_7	030265	0	1	0	0	1	0	0	1	0	1	0	0	0	0	0	0	0	0	0	0	0	0	1	0	20.8
2014_7	010364	1	1	0	1	1	0	0	0	0	0	0	0	0	0	0	0	0	0	1	0	0	0	0	0	20.8
2014_7	160364	1	1	0	1	0	0	1	1	0	0	0	1	0	0	0	0	0	1	1	0	0	0	1	0	37.5
2014_7	150476	1	1	1	1	0	0	0	1	0	0	0	1	0	0	0	0	0	0	0	0	0	0	0	0	25.0
2014_7	070567	1	1	1	1	0	0	1	1	0	0	0	1	0	0	0	0	1	1	1	0	0	0	0	0	41.7
2014_7	311055	1	1	1	1	0	0	1	1	0	1	0	1	1	0	0	0	0	0	1	0	0	0	1	0	45.8
2014_7	300177	1	1	1	1	0	1	1	1	0	0	0	0	1	0	0	0	0	0	0	0	0	0	0	0	33.3
2014_7	181063	0	0	1	0	0	0	0	1	0	1	0	1	0	0	0	0	1	0	0	0	1	0	0	0	25.0
2014_7	150863	0	1	1	0	0	0	1	1	0	0	0	0	0	0	0	0	1	1	1	0	1	1	0	0	37.5
2014_7	121270	0	1	1	1	0	1	1	1	0	1	0	0	0	0	0	0	0	1	1	0	1	0	0	0	41.7
2014_7	110366	0	1	1	1	0	1	0	1	0	1	0	0	0	0	0	0	1	0	0	0	1	0	0	0	33.3
2014_7	130562	0	0	1	1	0	0	0	0	0	0	0	1	0	0	0	1	1	1	0	0	0	0	0	0	25.0
2014_7	100565	0	1	1	1	0	1	1	1	0	0	0	0	0	0	0	1	1	0	1	0	1	0	0	0	41.7
2014_7	280762	0	0	1	0	0	1	0	1	0	0	0	1	0	0	0	0	1	1	0	0	0	1	0	0	29.2
2014_7	240265	0	0	1	1	0	0	0	1	0	0	0	1	0	0	0	0	1	0	1	0	1	0	0	0	29.2
2014_7	101163	1	0	1	0	0	0	1	0	0	0	1	0	0	1	0	0	0	0	0	0	1	0	0	1	29.2
2014_7	300363	1	0	1	1	0	0	1	0	0	0	1	0	0	0	1	0	0	0	0	0	1	0	1	0	33.3
2014_7	180662	1	0	1	1	0	1	0	0	0	0	0	1	0	0	0	1	0	0	0	0	1	0	1	0	33.3
2014_7	161164	1	0	1	1	0	0	0	1	0	1	0	0	0	1	0	0	1	1	0	1	0	0	1	0	41.7

2014_7	010556	1	0	1	0	0	1	0	1	0	1	1	0	0	0	0	0	1	0	1	0	0	0	1	0	37.5
2014_7	090868	0	1	1	1	0	0	1	1	0	1	0	0	1	0	1	1	1	1	0	0	0	0	0	0	45.8
2014_7	171165	0	1	1	1	0	0	1	1	0	1	0	0	0	0	0	0	1	1	1	0	1	1	0	0	45.8
2014_7	300362	1	1	1	0	0	0	1	1	0	0	0	1	1	0	0	0	0	1	1	0	0	0	0	0	37.5
2014_7	050264	0	0	0	0	0	0	0	1	0	0	0	0	0	0	0	0	0	0	0	0	0	0	1	1	12.5
2014_7	230868	1	1	1	1	0	1	1	1	0	0	0	0	1	0	0	1	0	1	1	0	0	0	0	0	45.8
2014_7	120671	1	1	1	0	0	0	1	1	0	0	0	0	0	0	0	0	1	0	1	0	0	0	0	0	29.2
2014_7	250464	1	0	1	1	0	0	0	1	0	1	0	1	0	0	0	0	0	0	1	0	0	0	0	0	29.2
2014_7	030866	1	1	1	1	0	1	0	0	0	1	0	0	0	1	1	0	0	0	0	0	0	0	0	1	37.5
2014_7	280553	1	1	1	1	0	0	1	1	0	1	0	0	1	0	0	0	1	1	1	0	0	0	1	0	50.0
2014_7	130963	1	1	1	1	0	0	0	0	0	1	0	0	1	0	0	0	0	1	1	1	1	0	1	1	50.0
2014_7	231066	1	1	1	1	0	0	0	0	0	1	0	0	1	0	0	0	0	1	1	1	1	0	1	1	50.0
2014_7	100768	1	1	1	0	1	1	0	0	0	1	0	0	0	0	1	0	0	1	0	1	0	0	0	1	41.7
2014_7	181066	1	1	1	0	0	1	0	1	0	1	0	0	0	1	0	0	0	1	0	0	0	0	0	0	33.3
2014_7	170462	1	1	1	1	1	1	1	1	0	1	1	0	0	1	0	0	0	0	1	0	0	0	0	0	50.0
2014_7	060867	1	1	1	1	1	1	1	1	0	1	1	0	0	1	1	0	0	0	0	0	1	0	0	1	58.3
2014_7	100866	1	0	1	1	1	0	1	0	1	1	0	1	1	1	0	1	0	1	1	1	1	0	0	0	62.5
2014_7	090970	1	1	1	1	0	0	1	1	0	1	0	0	0	0	0	0	1	1	1	0	1	0	0	0	45.8
2014_7	150358	1	0	1	1	0	0	0	1	0	1	1	0	0	0	0	0	0	1	1	0	0	0	0	0	33.3
2014_7	030667	1	1	1	1	0	1	1	1	0	1	0	0	1	0	1	0	0	1	1	1	1	0	0	0	58.3
2014_7	140274	1	1	1	1	0	1	1	1	0	1	0	0	1	0	1	0	0	1	1	1	1	0	0	0	58.3
2014_7	310764	0	1	1	1	0	1	1	0	0	0	0	0	1	0	1	1	0	1	1	0	0	1	0	0	45.8
2014_8	270364	1	1	0	1	1	1	1	1	0	0	1	1	0	0	0	0	0	1	0	0	1	1	0	1	54.2
2014_8	290767	0	0	1	0	0	1	0	1	0	1	0	0	0	0	1	1	0	1	0	0	0	0	0	0	29.2
2014_8	241165	1	0	0	1	1	0	0	1	0	1	0	0	0	0	0	0	0	0	1	0	1	0	0	0	29.2
2014_8	230267	0	0	1	1	0	1	1	1	0	1	0	0	0	1	0	0	1	0	1	1	1	0	0	0	45.8
2014_8	010663	1	1	1	1	0	0	0	1	0	1	1	0	0	1	0	0	1	0	0	0	0	0	0	0	37.5
2014_8	130471	1	1	1	1	1	1	1	1	0	1	1	1	0	0	0	0	1	1	1	1	0	0	0	1	66.7
2014_8	151064	1	1	0	1	0	0	1	0	0	0	0	0	0	1	1	0	0	1	0	1	0	0	0	1	37.5
2014_8	170465	0	0	1	1	0	0	0	0	0	0	0	0	0	1	0	0	1	0	0	0	0	0	1	0	20.8
2014_8	090859	0	1	1	1	1	0	1	0	1	1	0	1	1	1	1	0	1	1	0	0	0	0	0	1	58.3
2014_8	281263	1	1	1	1	1	0	0	1	0	1	1	1	0	1	0	0	0	1	0	0	1	0	1	0	54.2
2014_8	261254	1	1	0	1	1	0	1	1	0	1	1	1	0	1	0	0	0	1	1	0	0	0	1	0	54.2
2014_8	101058	1	1	1	1	1	0	1	1	0	0	1	1	0	1	0	0	1	0	1	0	0	0	0	0	50.0
2014_8	230964	1	1	1	1	0	0	0	1	0	0	0	1	1	0	1	0	1	0	0	1	0	0	1	1	50.0
2014_9	160259	1	0	1	0	0	0	1	0	0	0	1	0	0	0	0	1	1	0	0	0	0	1	0	1	33.3
2014_9	120570	1	1	1	0	0	0	1	1	0	1	0	1	0	1	0	0	0	0	0	0	0	0	0	1	37.5
2014_9	300164	1	0	1	1	0	1	1	1	0	0	0	0	0	0	0	0	1	1	0	0	0	0	1	0	37.5
2014_9	151163	1	1	0	1	0	1	0	1	0	1	1	0	0	0	0	0	0	1	0	1	1	1	1	0	50.0
2014_9	171060	1	0	1	1	1	0	1	1	0	0	1	0	0	1	1	1	0	0	0	0	0	0	0	0	41.7
2014_9	240765	1	1	1	1	0	1	1	0	0	0	0	0	0	0	0	0	0	1	1	1	1	0	0	1	45.8
2014_9	220364	1	0	1	1	0	0	1	1	0	1	1	0	0	0	0	0	1	0	0	0	0	0	0	0	33.3
2014_9	081066	1	1	1	1	0	0	1	1	0	1	1	0	1	1	0	0	0	0	0	1	0	0	0	1	50.0
2014_9	060363	1	1	1	1	0	0	1	1	0	1	0	0	1	0	0	0	0	1	0	0	1	1	1	0	50.0
2014_9	280769	1	1	1	1	0	0	1	1	0	1	0	0	1	0	0	0	1	1	0	0	0	1	0	0	45.8
2014_9	NA	1	1	0	1	0	0	0	1	0	1	0	0	0	1	0	1	1	1	0	0	0	1	0	0	41.7
2014_9	030163	1	1	1	1	0	0	1	1	0	1	1	0	0	1	0	1	1	0	1	0	1	0	0	0	54.2
2014_9	090762	0	1	1	1	1	0	1	0	0	0	0	1	0	0	0	1	1	1	0	0	0	0	0	0	37.5
2014_9	160666	1	1	1	1	0	0	0	0	0	1	1	1	1	0	0	0	1	0	0	0	0	0	0	0	37.5
2014_9	090569	0	1	0	1	0	0	1	1	0	0	1	1	1	0	1	0	0	0	0	0	0	1	0	1	41.7
2014_9	230861	1	1	1	0	0	1	0	1	0	0	0	1	0	0	0	1	1	1	0	0	0	0	0	1	41.7
2014_9	110160	0	1	1	0	1	0	0	1	0	1	0	1	0	0	0	1	0	1	0	0	0	1	0	1	41.7
2014_9	280868	1	1	1	1	0	0	1	1	0	0	1	1	0	0	1	1	0	1	1	0	0	0	0	0	50.0
2014_9	220865	1	1	0	1	0	0	1	1	0	1	1	1	0	0	1	1	0	0	0	1	0	0	0	1	50.0
2014_9	140865	1	0	0	1	0	0	1	1	0	1	1	0	0	0	1	1	1	0	1	1	1	0	0	1	54.2
2014_9	070658	1	0	0	1	1	0	1	0	0	1	1	0	1	0	1	1	1	1	0	1	0	0	0	0	50.0

2014_9	200360	1	1	0	0	0	0	1	1	0	1	0	0	0	0	0	0	1	0	0	0	1	0	0	0	29.2
2014_9	110664	1	1	1	1	0	0	1	0	0	1	1	1	0	1	1	0	1	1	1	1	0	0	0	1	62.5
2014_9	260263	0	1	1	0	0	1	1	0	0	1	0	1	0	0	0	0	1	0	1	0	0	0	0	1	37.5
2014_9	260766	1	1	1	1	0	0	1	1	1	1	0	1	1	0	1	1	1	0	0	0	1	0	0	0	58.3
2014_9	151266	1	1	1	1	1	1	1	0	0	1	0	0	0	0	1	0	0	0	1	0	1	0	0	1	50.0
2014_9	271054	1	0	1	1	0	1	0	1	0	1	0	0	0	0	0	0	1	1	0	0	0	1	0	0	37.5
2014_9	260465	0	1	1	1	1	1	1	1	0	1	0	0	1	0	1	1	1	0	1	1	0	0	0	0	58.3
2014_9	181070	0	1	0	1	0	0	1	1	0	1	0	1	1	1	1	0	1	1	1	0	1	0	0	0	54.2
2014_9	221160	1	1	1	1	0	0	1	1	0	1	1	0	0	0	1	0	0	1	0	0	1	0	0	1	50.0
2014_9	010862	0	1	1	1	0	0	1	1	0	1	0	0	0	1	0	0	1	0	1	0	0	0	0	0	37.5
2014_9	250168	0	1	0	1	1	0	1	1	0	1	0	0	0	0	1	0	1	0	1	0	0	0	0	0	37.5
2014_9	060762	1	1	1	1	1	0	1	1	0	1	0	1	1	1	0	0	1	0	1	0	0	0	0	0	54.2
2014_9	290368	1	1	0	1	0	0	0	1	0	0	0	0	1	0	0	0	1	0	1	0	1	0	0	0	33.3
2014_10	150163	1	1	1	1	1	0	1	1	1	0	1	0	1	1	0	0	1	1	1	1	1	1	0	0	70.8
2014_10	170659	1	1	1	1	1	0	1	1	0	0	0	1	0	1	1	0	0	0	1	0	1	0	0	0	50.0
2014_10	220174	1	1	1	1	1	1	1	1	1	1	0	1	0	1	0	0	0	1	1	0	0	0	0	1	62.5
2014_10	130965	1	0	1	1	0	1	1	0	0	0	1	0	0	0	0	0	1	1	1	1	0	0	0	0	41.7
2014_10	290862	1	1	1	0	1	0	1	1	0	1	0	1	0	1	1	0	1	1	1	0	1	0	0	0	58.3
2014_10	171063	1	0	1	1	1	1	1	1	1	1	1	1	0	1	1	0	1	1	1	1	1	1	0	1	83.3
2014_10	140668	1	1	0	1	0	1	1	1	0	1	1	1	1	0	0	0	1	0	0	0	1	0	0	0	50.0
2014_10	060663	0	1	0	1	1	0	1	0	0	1	1	0	0	0	1	0	1	0	0	1	0	0	0	0	37.5
2014_10	140564	1	1	1	1	1	1	1	1	0	1	1	1	0	1	0	0	1	0	0	0	0	0	0	0	54.2
2014_10	110363	1	0	0	0	1	0	1	1	0	1	1	0	0	1	0	0	0	0	0	0	0	0	0	0	29.2
2014_10	181065	1	0	0	1	0	1	0	1	0	1	1	1	1	0	0	0	0	1	1	1	1	0	0	0	50.0
2014_10	071264	0	1	0	1	0	1	1	1	0	1	0	1	0	1	1	0	0	1	1	1	0	0	0	1	54.2
2014_10	231163	1	1	0	1	0	0	0	0	0	1	0	0	1	0	1	0	1	1	0	1	0	0	0	0	37.5
2014_10	230565	1	0	0	1	0	1	1	0	0	0	1	1	1	1	0	0	0	0	1	0	1	0	0	1	45.8
2014_10	230566	1	1	0	1	1	0	1	1	0	1	1	0	0	1	1	1	1	1	1	0	0	1	0	0	62.5
2014_10	111064	1	1	1	1	0	0	1	1	0	1	0	1	1	0	0	0	1	1	1	0	0	1	0	0	54.2
2014_11	010460	0	1	1	1	1	1	1	0	0	1	0	1	0	1	0	1	1	0	0	0	1	0	0	1	54.2
2014_11	140859	0	1	1	1	1	0	1	1	0	1	0	1	0	1	1	0	1	0	0	1	1	0	0	1	58.3
2014_11	080368	0	1	0	1	0	0	1	1	0	0	0	0	0	0	0	0	0	1	1	0	0	0	1	0	29.2
2014_11	221266	0	1	1	1	1	0	0	1	0	0	0	1	0	1	0	0	0	1	1	0	0	0	0	0	37.5
2014_11	130564	0	1	0	1	1	0	1	0	0	0	1	1	0	1	0	0	0	0	1	1	0	1	0	1	45.8
2014_11	090867	1	1	0	1	1	1	1	1	0	1	0	1	0	0	1	0	0	1	1	0	0	0	0	0	50.0
2014_11	100657	1	1	1	1	1	0	1	1	0	0	0	1	1	1	1	1	1	1	0	1	0	0	0	1	66.7
2014_11	110761	1	1	1	1	1	0	1	1	0	0	0	1	0	0	0	0	1	0	0	0	0	0	0	0	37.5
2014_11	310765	1	1	1	0	1	0	1	1	0	0	0	1	0	0	0	0	1	0	0	0	0	0	1	0	37.5
2014_11	231263	0	1	0	1	0	1	0	1	0	1	1	0	0	1	0	0	1	0	1	0	1	0	1	0	45.8
2014_11	021066	1	1	1	1	1	0	1	1	0	0	0	1	0	1	0	0	1	0	0	0	0	0	0	0	41.7
2014_11	060968	0	1	0	1	1	0	1	1	0	1	0	0	0	1	0	0	1	0	1	1	0	0	1	0	45.8
2014_11	280361	0	1	0	1	0	0	1	1	0	0	0	0	0	1	1	0	1	0	0	1	1	0	0	1	41.7
		72.3	75.0	66.7	83.0	33.3	39.4	73.9	65.9	5.3	61.4	37.1	41.7	23.5	37.9	29.5	15.2	46.2	44.7	54.5	23.9	36.4	15.9	20.5	24.6	
